# ACSS2 controls PPARγ activity homeostasis to potentiate adipose-tissue plasticity

**DOI:** 10.1038/s41418-024-01262-0

**Published:** 2024-02-08

**Authors:** Nuo Chen, Ming Zhao, Nan Wu, Yaxin Guo, Baihui Cao, Bing Zhan, Yubin Li, Tian Zhou, Faliang Zhu, Chun Guo, Yongyu Shi, Qun Wang, Yan Li, Lining Zhang

**Affiliations:** 1 Department of Immunology, School of Basic Medical Science, Cheeloo College of Medicine, Shandong University, Jinan, China; 2 Department of Pathogen Biology, School of Basic Medical Science, Cheeloo College of Medicine, Shandong University, Jinan, China

**Keywords:** Metabolic disorders, Gene regulation, Molecular biology

## Abstract

The appropriate transcriptional activity of PPARγ is indispensable for controlling inflammation, tumor and obesity. Therefore, the identification of key switch that couples PPARγ activation with degradation to sustain its activity homeostasis is extremely important. Unexpectedly, we here show that acetyl-CoA synthetase short-chain family member 2 (ACSS2) critically controls PPARγ activity homeostasis via SIRT1 to enhance adipose plasticity via promoting white adipose tissues beiging and brown adipose tissues thermogenesis. Mechanistically, ACSS2 binds directly acetylated PPARγ in the presence of ligand and recruits SIRT1 and PRDM16 to activate UCP1 expression. In turn, SIRT1 triggers ACSS2 translocation from deacetylated PPARγ to P300 and thereafter induces PPARγ polyubiquitination and degradation. Interestingly, D-mannose rapidly activates ACSS2-PPARγ-UCP1 axis to resist high fat diet induced obesity in mice. We thus reveal a novel ACSS2 function in coupling PPARγ activation with degradation via SIRT1 and suggest D-mannose as a novel adipose plasticity regulator via ACSS2 to prevent obesity.

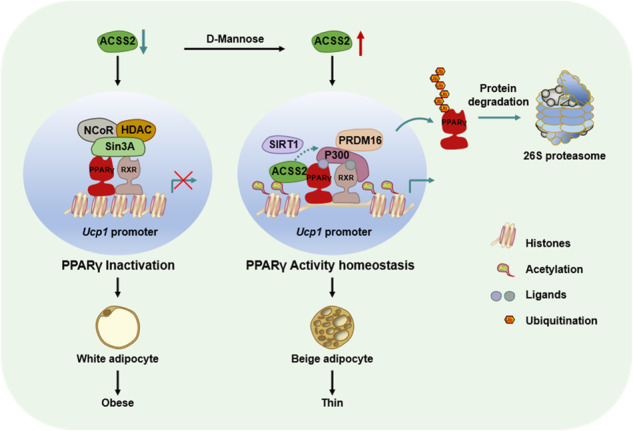

## Introduction

Obesity is currently the most prevalent chronic metabolic disorder and increases the risk of developing cancer and other diseases [1–4]. Excess fat accumulation in obesity impairs adipose tissues plasticity including metabolic, structural and phenotypic remodeling in response to various physiologic conditions, such as the adaptive thermogenesis in brown adipose tissues (BAT) and white adipose tissues (WAT) beiging [5]. Brown and beige adipocytes contain high mitochondrial density and expression of uncoupling protein (UCP1) and increase whole-body energy expenditure by over 100% in mice and by 40–80% in humans when fully active, showing that they possess high plasticity [6, 7]. Therefore, elevation in energy expenditure and adipose tissues plasticity by promoting WAT beiging and BAT thermogenesis is an effective strategy to treat obesity-related disorders.

Peroxisome proliferator-activated gamma (PPARγ) is a ligand-activated member of the nuclear hormone receptor superfamily and controls adipocyte differentiation and function, and thus becomes a classic target to treat obesity-related disorders [1, 8]. It contains an N-terminal domain involved in ligand-independent activation function (AF1), a DNA binding domain (DBD), and a C-terminal ligand-binding domain (LBD) including the ligand-dependent activation function 2 (AF2) [9]. In response to ligand, exchange of transcriptional activation complex including CBP/P300 and SIRT1, and transcriptional inhibition complex including NR corepressor (NCoR), silencing mediator for retinoid and thyroid receptors (SMRT) and histone deacetylases (HDACs) occurs to the heterodimer of PPARγ and retinoid X receptor (RXR) [10, 11]. The brown adipose-specific and thermogenetic markers, such as PRDM16 and UCP1, are classic PPARγ targets to promote WAT beiging and BAT adaptive thermogenesis [12–14]. Therefore, activation of PPARγ in adipocyte by the thiazolidinedione (TZD) class of insulin-sensitizing drugs (TZDs) can improve obesity and insulin sensitivity. However, fully activated PPARγ by TZDs leads to the drawback of increased adipogenesis and weight gain. Besides, PPARγ overactivation can induce its dominant-negative splice isoform PPARγΔ5 generation as well to impair its function and adipocyte differentiation [15]. In contrast, genetic studies in mouse models of heterozygous PPARγ deficiency demonstrate that the moderately decreased PPARγ in the absence of TZDs is as effective as PPARγ agonists in improving insulin sensitivity [16–19]. The polyubiquitination and degradation of PPARγ drived by its AF2 domain, as a ubiquitin-dependent degradation signal, is required for PPARγ activation [9]. Taken together, PPARγ transcriptional activity homeostasis is critical for its function. However, the molecule that determines PPARγ transition from activation to degradation in order to sustain its activity homeostasis remains vague.

The acetyl-CoA synthetase short-chain family member 2 (ACSS2) generates acetyl-CoA from acetate to support tumor growth, the biosynthesis of cholesterol, glucose and fatty acid, as well as ketogenesis and protein acetylation [20–23]. Importantly, the phosphorylation of ACSS2 by AMPK can enter into the nuclear and boost local histone acetylation of lysosome- and memory- associated genes [24, 25]. The acetyl-CoA from ACSS2 can also be utilized for liver lipogenesis with high fructose while in adipocyte, ACSS2 has little effect on adipocyte differentiation and formation [26, 27]. Intestinal ACSS2 promotes lipid absorption to facilitate lipid accumulation in AT [28]. However, its physiological role in regulating thermogenic adipose tissue’s function is yet to be determined.

D-Mannose, a C-2 epimer of glucose, is naturally present in many plants and fruits and has multiple benefits for human in suppressing immunopathology including autoimmune diabetes and airway inflammation and tumor growth [29–31]. Intracellular D-mannose can activate AMPK and enhance fatty acid oxidation (FAO) to promote acetyl-CoA generation. Unlike glucose, it can function as prebiotics to alter gut microbiota in obese mice [32]. Although D-mannose shares the same transporters with glucose, it only prevents glucose utilization and glycolysis, rather than influences glucose uptake, suggesting that it is a potent and rapid-acting regulator of glucose metabolism [30, 33]. Adipose tissues possesses a high capacity for utilizing glucose to regulate systemic glucose homeostasis and both the absence and excess of adipose tissues may cause severe impairment of glucose homeostasis and diabetes [34, 35]. Therefore, as a rapid-acting regulator of glucose metabolism, D-mannose function in adipose tissues needs investigation.

Here, we show that ACSS2 is a potent co-regulator of PPARγ and RXRα to enhance WAT beiging and BAT thermogenesis. Unexpectedly, ACSS2 recruits SIRT1 to deacetylate PPARγ and thereafter induces its ubiquitination and degradation to prevent PPARγ overactivation. Therefore, ACSS2 can tightly couple histone acetylation with PPARγ activity homeostasis via SIRT1. As ACSS2 to be a therapeutic target for obesity, we further discovered that oral D-mannose rapidly targeted adipose tissues and enhanced nuclear ACSS2 level by activating AMPK and enhanced energy expenditure to combat high fat diet (HFD)-induced obesity, liver lipid dysfunction and insulin resistance. We thus reveal a previously unrecognized ACSS2 function in adipose tissues plasticity via SIRT1-PPARγ axis and suggest D-mannose as a novel ACSS2 inducer to treat obesity-related disorders.

## Results

### Adipose ACSS2 promotes ingWAT beiging and BAT thermogenesis

**Fig. 1:**
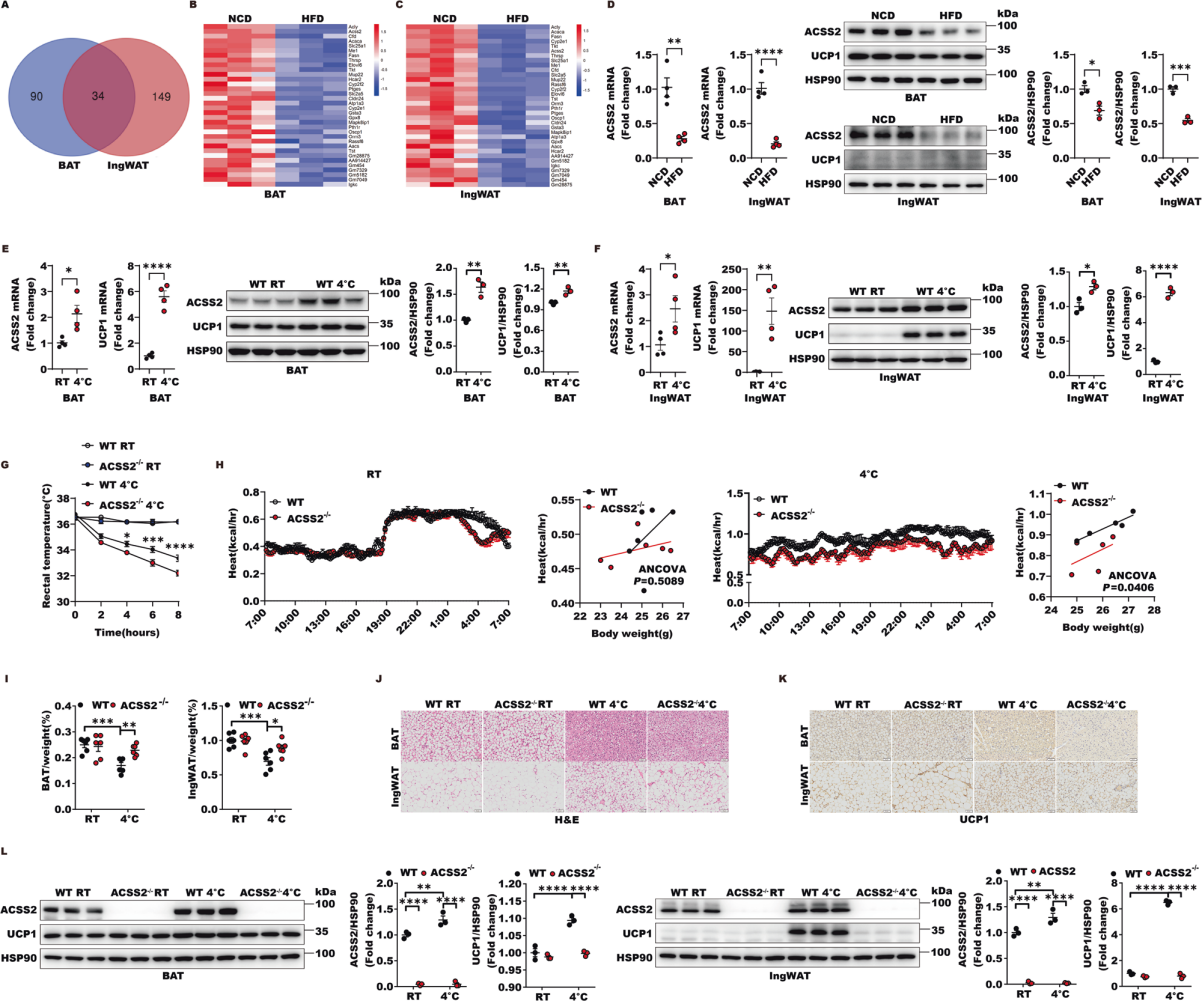
Adipose ACSS2 promotes WAT beiging and BAT thermogenesis. **A**–**D** Wild type male mice at 8 weeks of age were maintained on NCD (normal-chow-diet) or HFD (60% fat) for 24 weeks. **A** The Venn diagrams showed the down-regulated genes of BAT (90 genes), ingWAT (149 genes) individually and 34 genes including 27 genes encoding protein and 7 unknown functions genes were both down-regulated in BAT and ingWAT in HFD mice compared with those of NCD mice (*n* = 6 per group, two samples were mixed together). **B** Heat map of the 34 down-regulated genes of BAT in HFD mice versus NCD mice was shown (*n* = 6 per group, two samples were mixed together). **C** Heat map of the 34 down-regulated genes of ingWAT in HFD mice versus NCD mice was shown (*n* = 6 per group, two samples were mixed together). **D** The expression of ACSS2 and UCP1 in the BAT and ingWAT of the NCD or HFD mice (*n* = 3–4 per group) were detected. **E**, **F** 6–8-week-old wild type male mice were challenged with or without cold exposure at 4 °C for 16 h. The lysates from BAT (**E**) or ingWAT (**F**) were subjected to mRNA anaylsis or western blot with indicated antibodies. The quantitative analyses of ACSS2 and UCP1 were performed (*n* = 3–4 per group). **G** The rectal temperature of 6–8-week-old wild type or *Acss2*^−/−^ male mice exposed to cold (4 °C for 8 h) were shown (*n* = 5 per group). **H** The metabolic cage experiments were performed in 8–12-week-old *Acss2*^−/−^ male mice or wild type male mice with or without cold exposure. Heat production and regression-based analysis of absolute heat production against body weight of WT mice and *Acss2*^−/−^ mice were shown (*n* = 5–6 per group). **I** Relative weight of BAT and ingWAT (normalized to body weight) from 6–8-week-old wild type or *Acss2*^−/−^ male mice challenged with or without cold exposure at 4 °C for 16 h were shown (*n* = 6 per group). **J**, **K** Representative H&E (**J**) or immunohistochemistry (**K**) for UCP1 of BAT and ingWAT from 6–8-week-old wild type or *Acss2*^−/−^ male mice challenged with or without cold exposure at 4 °C for 16 h were shown. **L** Tissue lysates from BAT or ingWAT of WT or *Acss2*^−/−^ mice with or without cold exposure at 4 °C for 16 h were subject to western blot with indicated antibodies. The quantitative analyses of ACSS2 and UCP1 were performed. (*n* = 3 per group).

To identify the novel protein that regulates the adaptive thermogenesis of thermogenic fat, we screened the down-regulated genes in both interscapular BAT (BAT) and subcutaneous inguinal WAT (ingWAT) of obese mice compared with those of lean mice. Total 34 down-regulated genes were identified and the top ranking genes that are highly expressed in thermogenic fat were *Acly*, *Acss2*, *Acaca*, *Cyp2e1* and others (Fig. 1A–C, Tables S1–3). Among them, we noticed that the expression level of acetyl-CoA synthetase ACSS2 was highly present in adipose tissues, including WAT and BAT and more importantly, it was also obviously reduced in BAT and ingWAT of HFD-mice compared with those of lean mice (Figs. 1D, S1A). Exposure to cold activates BAT adaptive thermogenesis and WAT beiging. In this process, UCP1 is a critical thermogenic effector. Compared with the control mice (which were kept in room temperature, 22 °C), the expression levels of ACSS2 and UCP1 were both significantly up-regulated in BAT and ingWAT after 16 h of cold stimulation (Fig. 1E, F), suggesting that ACSS2 may play a role in adaptive thermogenesis. Therefore, we further evaluated the ability of ACSS2 in promoting thermogenic fat activation. *Acss2*^*−/−*^ mice and wild-type mice were placed at 4 °C for different time courses, and their temperature and energy expenditure were measured. Under the cold stimulation, we noticed that *Acss2*^*−/−*^ mice had a lower core body temperature than wild-type mice (Fig. 1G). Also, *Acss2*^*−/−*^ mice had lower rates of O2 consumption (Fig. S1B), heat production (Fig. 1H) and CO2 emission (Fig. S1C), but no significant differences in respiratory exchange ratio (RER) (Fig. S1D) or food intake (Fig. S1E) were observed. In response to the room temperature, their body temperatures, the rates of O2 consumption or CO2 emission, and the abilities of heat production showed no detectable differences (Fig. 1G, H, Fig. S1B, C). Moreover, cold stimulated *Acss2*^*−/−*^ mice displayed lesser morphological changes and weight reductions of BAT and ingWAT compared with the wild type mice (Fig. 1I, S1F). Hematoxylin and eosin (H&E) and immunohistochemistry staining of sections revealed less robust emergence of multilocular, beige-like adipocytes and lower intensity of UCP1 protein signals in *Acss2*^*−/−*^ mice following cold exposure (Fig. 1J, K). Meanwhile, the protein levels of UCP1 were significantly decreased in BAT and ingWAT in *Acss2*^*−/−*^ mice after cold stimulation (Fig. 1L). These differences between wild type and *Acss2*^*−/−*^ mice were not observed in the room temperature (Fig. 1J–L). These data suggested that ACSS2 influences thermogenic fat adaptive thermogenesis, including BAT thermogenesis and ingWAT beiging.

### ACSS2 binds PPARγ to transactivate *Ucp1*

**Fig. 2:**
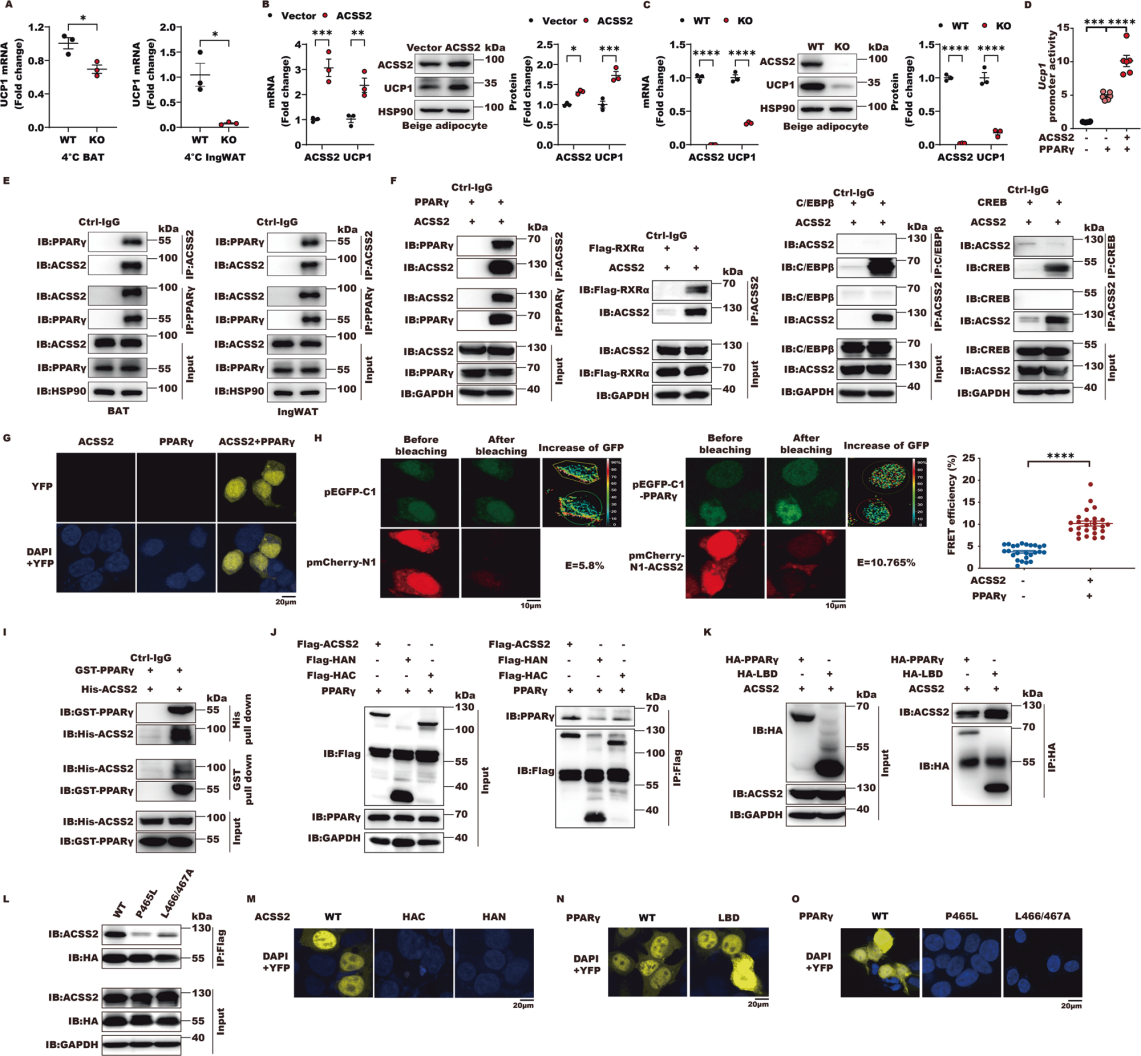
ACSS2 binds PPARγ to transactivate *Ucp1*. **A** 6–8-week-old wild type or *Acss2*^−/−^ male mice were challenged with or without cold exposure at 4 °C for 16 h. The mRNA levels of *Ucp1* in BAT and ingWAT were shown ( = 3 per group). **B** The differentiated beige adipocytes in vitro from the stromal vascular fraction (SVF) of the ingWAT in wild type mice were transfected with 2 μg ACSS2 or control plasmids for 24 h. The mRNA and protein levels of ACSS2 and UCP1 were evaluated by qRT-PCR and western blot (*n* = 3 biological replicates). **C** The mRNA and protein levels of ACSS2 and UCP1 were then evaluated in the differentiated beige adipocytes from wild type or *Asss2*^*−/−*^ mice by qRT-PCR and western blot (*n* = 3 biological replicates). **D** The luciferase activities of *Ucp1* promoter in the presence or absence of PPARγ and ACSS2 in HEK293T cells were examined (*n* = 6 biological replicates). **E** Representative immunoblot of endogenous immunoprecipitation (IP) in BAT and ingWAT (*n* = 3 biological replicates). **F** Representative immunoblot of exogenous IP of FLAG-tagged ACSS2 or HA-tagged PPARγ, FLAG-tagged RXRα, CEBPβ and CREB in HEK293T cells (*n* = 3 biological replicates). **G** Representative fluorescent images of HEK293T cells transfected with 2 μg plasmid encoding ACSS2 or PPARγ fused to the fluorescent protein fragments indicated in each panel. DAPI stain demonstrated nuclear locus. The intensity YFP signal indicates the amounts and localization of BiFC complex (ACSS2-PPARγ) (*n* = 3 biological replicates). **H** Representative FRET images of HEK293T cells transfected with 2 μg plasmid encoding ACSS2 or PPARγ fused to the EGFP and mCherry indicated in each panel. The images before and after acceptor-photobleaching were shown as well and the FRET efficiency in the nuclear was calculated from individual cells (*n* = 25). **I** Representative immunoblot of pull-down assay of purified recombination proteins His-ACSS2 and GST-PPARγ from BL21 (DE3) (*n* = 3 biological replicates). **J** Representative immunoblot of exogenous IP of FLAG-tagged full length ACSS2, HAN or HAC with HA tagged PPARγ in HEK293T cells (*n* = 3 biological replicates). **K** Representative immunoblot of exogenous IP of FLAG-tagged ACSS2 and HA-tagged PPARγ or its LBD in HEK293T cells (*n* = 3 biological replicates). **L** Representative immunoblot of exogenous IP of FLAG-tagged ACSS2 and HA-tagged PPARγ or its mutants in HEK293T cells (*n* = 3 biological replicates). **M** Representative BiFC images of HEK293T cells transfected with ACSS2 or its truncated mutants and PPARγ fused to the fluorescent protein fragments indicated in each panel (*n* = 3 biological replicates). **N** Representative BiFC images of HEK293T cells transfected with ACSS2 and PPARγ or its LBD fused to the fluorescent protein fragments indicated in each panel (*n* = 3 biological replicates). **O** Representative BiFC images of HEK293T cells transfected with ACSS2 and PPARγ or its mutants fused to the fluorescent protein fragments indicated in each panel (*n* = 3 biological replicates).

As an acetyl coenzyme A synthase, ACSS2 is crucial for histone acetylation and gene transcription and we observed that ACSS2 was positively correlated with UCP1. We hypothesized that ACSS2 may directly participate in activating *Ucp1* transcription. Firstly, we found that ACSS2 knockout led to a significant decrease in the mRNA levels of *Ucp1* in BAT and ingWAT under cold challenge (Fig. 2A) but not at room temperature (Fig. S2A). In the differentiated beige adipocytes from the stromal vascular fraction (SVF) in the ingWAT of mice under the β3-adrenoceptor agonist CL316243 treatments, which mimics the cold challenge to activate adipose tissues adaptive thermogenesis in mice, ACSS2 overexpression remarkably increased *Ucp1* mRNA levels while its knockout decreased expressions of Ucp1 (Fig. 2B, C), supporting that ACSS2 influences *Ucp1* transcription in adipocytes. PPARγ is known to activate *Ucp1* transcription and we further found that ACSS2 significantly enhanced PPARγ-mediated activation of *Ucp1* promoter (Fig. 2D), indicating that ACSS2 may cooperate with PPARγ to regulate *Ucp1* transcription. To support their synergy in *Ucp1* activation, endogenous ACSS2 was immunoprecipitated with PPARγ in BAT and ingWAT (Fig. 2E). In line with this finding, ACSS2 was able to immunoprecipitate with PPARs as well and its heterodimer component RXRα in cell lines, but had no interactions with other classical transcriptional activators involved in glucose, lipid and amino acid metabolism, including CEBPβ and CREB (Figs. 2F, S2B, C), which suggested that ACSS2 was specifically co-immunoprecipated with PPARs. Using bimolecular fluorescent complimentary (BiFC) assay with bFos and bJun as a positive pair, we found that there were direct interactions between ACSS2 and PPARs predominantly in the nuclear rather than cytoplasm (Figs. 2G, S2D), which was important to facilitate target gene histone acetylation and transcription. ACSS2-PPARγ physical interaction was further confirmed by fluorescence resonance energy transfer (FRET), which occurred only between donor and acceptor with a distance less than 10 nm, and pull-down assay in vitro (Fig. 2H, I). For ACSS2, both its N- (HAN) and C-terminal (HAC) were required for PPARγ binding (Fig. 2J, M). PPARγ LBD possessed the same ACSS2 binding ability with the full length, suggesting that LBD domain was the principal subregion for ACSS2 contacting (Fig. 2K, N). To support it, ligand binding deficient mutants P465L and L466/467 A of PPARγ blocked ACSS2 binding in the nucleus (Fig. 2L, O). Taken together, ACSS2 interacts with PPARγ to transactivate *Ucp1* and the full length ACSS2 is required for PPARγ LBD binding.

### ACSS2 is recruited to acetylated PPARγ in a ligand-dependent manner

**Fig. 3:**
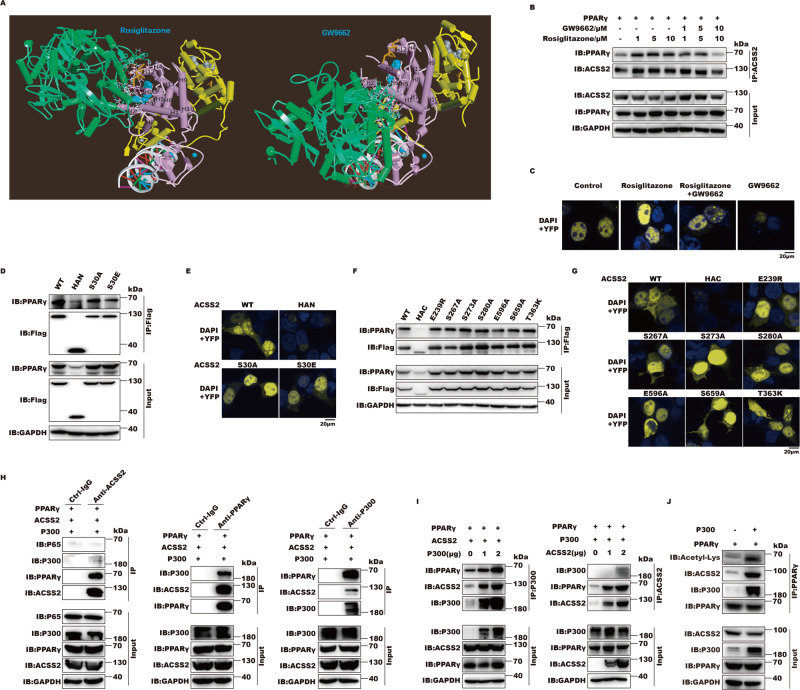
ACSS2 is recruited to acetylated PPARγ in a ligand-dependent manner. **A** ACSS2-PPARγ protein docking using *Z*-Dock server. Model represents the best among all the docking poses. Protein colored green is ACSS2 and protein colored purple is PPARγ; Protein colored yellow is RXRα; The white colored helix is PPRE elements DR1; Rosiglitazone (Left) and GW9662 (Right) are blue balls inside PPARγ; The blue balls around DNA are Zinc ion; The gray balls are 9-*cis*-retinoic acid for RxRα ligand. The brown sticks are NCoA2 Peptide around PPARγ and RXRα. **B** Representative immunoblot of exogenous IP of FLAG-tagged ACSS2 and HA-tagged PPARγ in response to rosiglitazone (0–10 μM) and GW9662 (0–10 μM) for 16 h in HEK293T cells (*n* = 3 biological replicates). **C** Representative BiFC fluorescent images of HEK293T cells transfected with 2 μg plasmid encoding ACSS2 or PPARγ fused to the fluorescent protein fragments indicated in each panel in response to 10 μM rosiglitazone and GW9662 alone or together for 16 h (*n* = 3 biological replicates). **D** Representative immunoblot of exogenous IP of FLAG-tagged ACSS2 or its *N*-terminal mutants with HA-tagged PPARγ in HEK293T cells (*n* = 3 biological replicates). **E** Representative BiFC fluorescent images of FLAG-tagged ACSS2 or its *N*-terminal mutants with HA-tagged PPARγ in HEK293T cells (*n* = 3 biological replicates). **F** Representative immunoblot of exogenous IP of FLAG-tagged ACSS2 or its C-terminal mutants with HA-tagged PPARγ in HEK293T cells (*n* = 3 biological replicates). **G** Representative BiFC fluorescent images of FLAG-tagged ACSS2 or its C-terminal mutants with HA-tagged PPARγ in HEK293T cells (*n* = 3 biological replicates). **H** Representative immunoblot of exogenous IP of FLAG-tagged ACSS2, HA-tagged PPARγ and HA-P300 in HEK293T cells (*n* = 3 biological replicates). **I** Representative immunoblot of exogenous IP of FLAG-tagged ACSS2, HA-tagged PPARγ and MYC-tagged P300 in HEK293T cells expressing PPARγ in response to increasing P300 and ACSS2 individually (*n* = 3 biological replicates). **J** Representative immunoblot of exogenous IP of HA-tagged PPARγ, P300, ACSS2 and acetylated proteins following transfection with PPARγ and P300 in HEK293T cells (*n* = 3 biological replicates).

Since ACSS2 can bind to PPARγ LBD, whether their interactions relied on ligand binding was further investigated. In line with our above observations, we found that ACSS2 bound PPARγ LBD and formed the triple complex with PPARγ and RXRα nuclear receptor on PPRE without influencing NCoA2 binding in the presence of rosiglitazone by molecular docking analysis. Conversely, GW9662 prevents ACSS2 loading to PPARγ-RXRα-DNA complex, where the predictive ACSS2-PPARγ-GW9662 binding would disrupt DNA structure so as not to form reasonable conformation (Fig. 3A). From the complex structure, ACSS2 rightly binds the edge of PPARγ-LBD pocket with rosiglitazone inside and contacts with helix2, 2’, 3, 5,6,11 and 12, whereas ACSS2 would interact with helix 1, 2 and 2’ and thus prevent PPARγ loading on PPRE DR1 in response to GW9662 (Fig. S3A, Table S4, 5). Co-IP and BiFC experiments further supported that rosiglitazone enhanced but GW9662 inhibited nuclear ACSS2-PPARγ interaction, suggesting that ACSS2 is induced to bind nuclear PPARγ by ligand and their interactions are dependent on ligand binding (Fig. 3B, C). As for ACSS2, its multiple phosphorylation mutations based on predicted ACSS2-PPARγ interaction face, including S30A and S30E in HAN and E239R, S267A, S273A, S280A, E596A, S659A and T363K in HAC did not affect the interaction with PPARγ (Fig. 3D–G, Table S4). Interestingly, ACSS2 nuclear localization deficient mutant S659A and T363K, which loses enzymatic activity to synthesize acetyl-CoA, still had ability to bind PPARγ. We speculated that ACSS2 serine 659 phosphorylation was not the only determinant for its nuclear localization and its enzymatic activity was indispensable for its function together with PPARγ rather than influencing PPARγ binding ability. Next, we wanted to identify ACSS2 function in regulating PPARγ activity. ACSS2 presented to specifically coimmunoprecipitate with PPARs and coactivator P300, but not NF-κB, which is also a co-regulator of P300, further supporting ACSS2 directly binds PPARs, rather than P300, to form ACSS2-PPARs-P300 complex (Figs. 3H, S3B, C). Furthermore, P300 promoted ACSS2 binding to PPARγ, but ACSS2 failed to recruit P300 to PPARγ in a dose dependent manner, implying that P300 is firstly induced to bind PPARγ to facilitate ACSS2 recruitment (Fig. 3I). Given that CBP/P300 was responsible for PPARγ acetylation in the presence of ligand, whether ACSS2 binds acetylated PPARγ was further investigated. We observed that P300 augmented ACSS2 binding to acetylated PPARγ, suggesting PPARγ acetylation favors ACSS2 recruitment (Fig. 3J). Collectively, ACSS2 is recruited to acetylated PPARγ by CBP/P300 in response to PPARγ ligand.

### ACSS2 regulates PPARγ transcriptional activity homeostasis by SIRT1

**Fig. 4:**
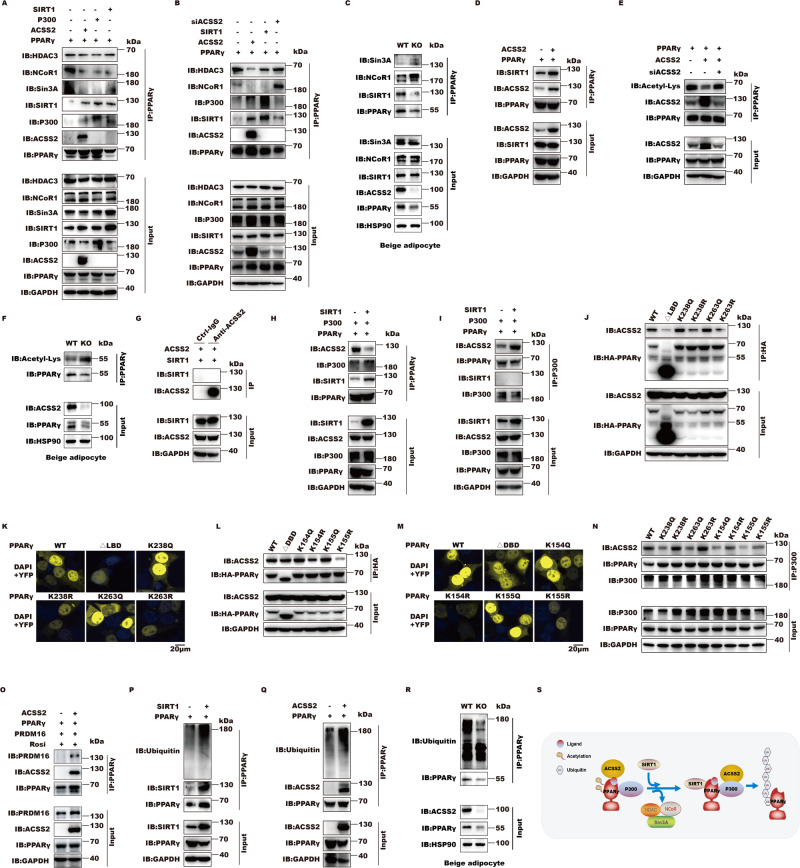
ACSS2 regulates PPARγ transcriptional activity by SIRT1. **A** Representative immunoblot of exogenous IP of HA-tagged PPARγ, HDAC3, Sin3A, NCoR1 and ACSS2 in HEK293T cells (*n* = 2 biological replicates). **B** Representative immunoblot of exogenous IP of HA-tagged PPARγ, HDAC3, NCoR1 and ACSS2 following transfection with PPARγ, ACSS2 and SIRT1 or small RNA interference against ACSS2 in HEK293T cells (*n* = 2 biological replicates). **C** Representative immunoblot of IP of PPARγ, Sirt1, Sin3A and NCoR1 in the differentiated beige adipocytes from wild type or *Asss2*^*−/−*^ mice (*n* = 2 biological replicates). **D** Representative immunoblot of exogenous IP of HA-tagged PPARγ, SIRT1 and ACSS2 in HEK293T cells (*n* = 2 biological replicates). **E** Representative immunoblot of exogenous IP of HA-tagged PPARγ, ACSS2 and acetylated proteins in HEK293T cells (*n* = 3 biological replicates). **F** Representative immunoblot of adipocyte endogenous PPARγ acetylations in the differentiated beige adipocytes from wild type or *Asss2*^*−/−*^ mice (*n* = 2 biological replicates). **G** Representative immunoblot of exogenous IP of ACSS2 and SIRT1 in HEK293T cells (*n* = 3 biological replicates). **H**, **I** Representative immunoblot of exogenous IP of HA-tagged PPARγ, SIRT1, P300 and ACSS2 in HEK293T cells (*n* = 3 biological replicates). **J**, **K** Representative immunoblot of exogenous IP of HA-tagged PPARγ (WT), its deletion mutant or acetylation mutants with ACSS2 in HEK293T cells (*n* = 2 biological replicates). **L**, **M** Representative BiFC images of HEK293T cells transfected with 2 μg plasmid encoding ACSS2 and PPARγ WT, LBD deletion mutant ΔLBD, DBD deletion mutant ΔDBD, K238Q, K238R, K263Q, K263R K154Q, K154R, K155Q or K155R mutants fused to the fluorescent protein fragments indicated in each panel (*n* = 2 biological replicates). **N** Representative immunoblot of exogenous IP of P300 and ACSS2 in HEK293T cells transfected with wild type PPARγ and its acetylation mutants (*n* = 2 biological replicates). **O** Representative immunoblot of exogenous IP of PPARγ, ACSS2 and PRDM16 in HEK293T cells (*n* = 2 biological replicates). **P** Representative immunoblot of exogenous IP of PPARγ, SIRT1 and ubiquitinated protein or (**Q**) PPARγ, ACSS2 and ubiquitinated protein in HEK293T cells (*n* = 2 biological replicates). **R** Representative immunoblot of adipocyte endogenous PPARγ ubiquitinations in the differentiated beige adipocytes from wild type or *Asss2*^*−/−*^ mice (*n* = 2 biological replicates). **S** Model of SIRT1 recruitment by ACSS2 to regulate PPARγ transcriptional activity homeostasis. P300-meidated ACSS2 recruitment to acetylated PPARγ can facilitate corepressors HDAC3, Sin3A and NCoR1 disassociation and SIRT1 loading. SIRT1 arrival deacetylated PPARγ, also trans-located ACSS2 from PPARγ to P300 to prevent its over-activation. Besides, SIRT1-mediated PPARγ deacetylation triggers its ubiquitination and degradation. In this scenario, ACSS2 controls PPARγ transcription activity through SIRT1.

ACSS2 is recruited to acetylated PPARγ by CBP/P300, so we next sought to identify what the output of this interaction is. We found that ACSS2 functioned like P300 and SIRT1 to prevent co-repressor HDAC, Sin3A and NCoR1 binding from PPARγ (Fig. 4A). Conversely, ACSS2 downregulation restored the interaction of HDAC and NCoR1 with PPARγ but abrogated P300 and SIRT1 loading (Fig. 4B). Next, we checked the binding abilities of SIRT1 and co-repressors (Sin3A, NCoR1) to PPARγ in the differentiated beige adipocytes from wild type or *Acss2*^−/−^ mouse by IP assays. As we expected, the recruitment of SIRT1 to PPARγ was significantly impaired while the disassociations of Sin3A and NCoR1 from PPARγ was prevented in response to ACSS2 deficiency, suggesting that adipose ACSS2 deletion prevents SIRT1 recruitment and co-repressors disassociation from PPARγ (Fig. 4C). SIRT1 has been reported to expel NCoR1 from PPARγ and deacetylate PPARγ [36]. We then observed that ACSS2 promoted SIRT1 to bind PPARγ and significantly reduced PPARγ acetylation (Fig. 4D, E). To further support this observation, the acetylation forms of PPARγ in the *Acss2*^*−/−*^ beige adipocytes was remarkably increased (Fig. 4F). During this process, ACSS2-SIRT1 interaction was not observed, suggesting that ACSS2-meidated PPARγ conformation change facilitates SIRT1 recruitment (Fig. 4G). However, SIRT1 loading in turn reduced PPARγ-ACSS2 but enhanced ACSS2-P300 interaction, suggesting that SIRT1 drives ACSS2 translocation from PPARγ to P300 (Fig. 4H, I). Surprisingly, we found that PPARγ LBD acetylation at K238 and K263 (Corresponding to lys268 and lys293 of PPARγ2) was required for ACSS2 loading while their mutations abolished ACSS2 binding (Figs. 4J, K, S4A). Other PPARγ DBD acetylation mutants, which were deacetylated by SIRT1 as well, including K154 and K155, influenced ACSS2 binding as well (Figs. 4L, M, S4A). Although these PPARγ acetylation defect mutants, including K154R, K155R, K238R and K263R, impaired ACSS2 binding, they augmented ACSS2-P300 interaction, which further supported SIRT1-mediated ACSS2 translocation from deacetylated PPARγ to P300 (Fig. 4N). However, P300 can acetylate PPARγ but SIRT1 performed PPARγ deacetylation. So how ACSS2 coordinates P300 and SIRT1 recruitment to PPARγ is further investigated. SIRT1-dependent deacetylation is required to recruit the BAT program coactivator PRDM16 to PPARγ, leading to selective induction of BAT genes like *Ucp1* [36]. We noticed that PRDM16 was recruited by ACSS2 to PPARγ (Fig. 4O), which supported that ACSS2 was able to transactivate *Ucp1*. Furthermore, both ACSS2 and SIRT1 recruitment induced subsequent non-acetylated PPARγ ubiquitination and degradation as well (Fig. 4P, Q). Consistent with our previous findings, adipose-specific ACSS2 deficiency eventually caused the decreased ubiquitination of PPARγ in the differentiated beige adipocytes (Fig. 4R, S). By analyzing the changes of PPARγ ubiquitination in response to the various E3 ligases targeted for PPARγ, which contain STUB1, Trim25, MDM2, Siah2, MKRN1, NEDD4 and Trim27, STUB1 and Trim25, might mediate ACSS2 promotion in PPARγ ubiquitination (Figure S4,B–H). These observations further supported that adipose ACSS2 deficiency can impair PPARγ activity homeostasis via SIRT1. From above observations, we conclude that ACSS2 tightly couples histone acetylation via CBP/P300 with PPARγ transcriptional activity through SIRT1, eventually conferring PPARγ transcriptional activity homeostasis and target gene proper transcription. During this process, the CBP/P300 and SIRT1 mediated PPARγ acetylation switch on K154, K155, K238 and K263 sequentially determines ACSS2-PPARγ formation and disassociation.

### Adipose-specific ACSS2 deletion impairs the adaptive thermogenesis of adipose tissues under the cold stimulation in mice

**Fig. 5:**
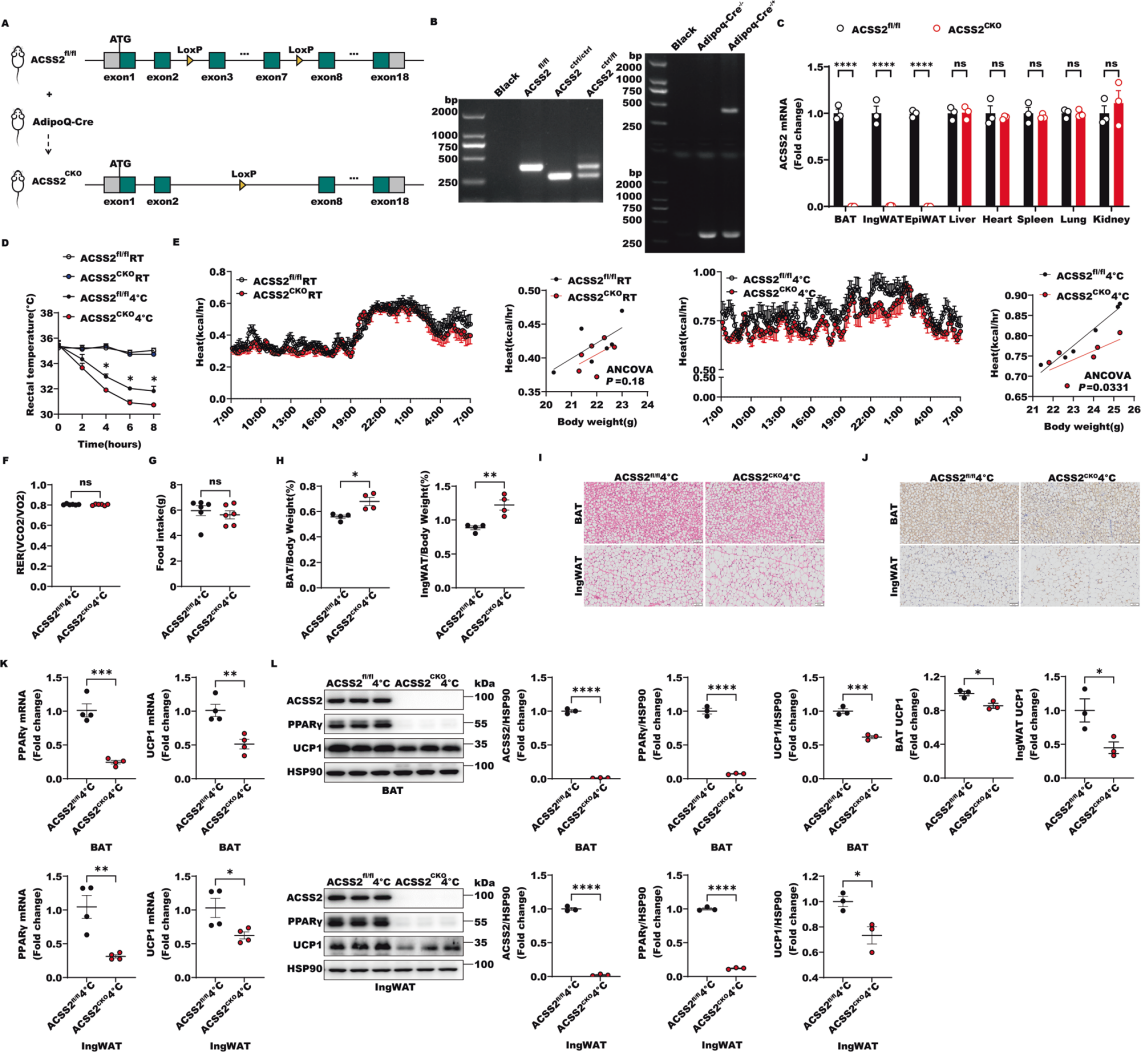
Adipose-specific ACSS2 deletion impairs the adaptive thermogenesis of adipose tissues under the cold stimulation in mice. **A** The adipose-specific *Acss2* knockout mice (*Acss2*^*floxp/floxp, cre+*^ (*Acss2*^*fl/fl*^, Adiponectin cre^+^), (*Acss2*^CKO^)) were generated by using the Cre/Loxp system by crossing *Acss2*^*floxp/floxp*^ and adiponectin-drived Cre-expressing mice. **B** The *Acss2*^CKO^ mice were genotyped using agarose gel electrophoresis. **C** The mRNA levels of *Acss2* in BAT, ingWAT, epiWAT, Liver, Heart, Spleen, Lung and Kidney from *Acss2*^*fl/fl*^ or *Acss2*^CKO^ mice were quantitatively analyzed (*n* = 3 per group). **D** The rectal temperature of 6–8-week-old *Acss2*^*fl/fl*^ or *Acss2*^CKO^ male mice exposed to cold (4 °C for 8 h) were shown (*n* = 3 per group). **E** Heat production and regression-based analysis of absolute heat production against body weight of *Acss2*^*fl/fl*^ or *Acss2*^CKO^ mice with or without cold exposure at 4 °C for 24 h were shown (*n* = 6 per group). **F** RER of *Acss2*^*fl/fl*^ or *Acss2*^CKO^ mice challenged with cold exposure at 4 °C for 24 h were shown (*n* = 6 per group). **G** Food intake of *Acss2*^*fl/fl*^ or *Acss2*^CKO^ mice challenged with cold exposure at 4 °C for 24 h were shown (*n* = 6 per group). **H** The quantitative analysis on the weight of BAT and ingWAT in mice from *Acss2*^*fl/fl*^ and *Acss2*^CKO^ challenged with cold exposure at 4 °C for 16 h was performed (*n* = 4 per group). **I**, **J** Representative H&E (**I**) or immunohistochemistry for UCP1 (**J**) of BAT and ingWAT from 6–8-week-old *Acss2*^*fl/fl*^ or *Acss2*^CKO^ male mice challenged with cold exposure at 4 °C for 16 h were shown, and the quantitative analyses of UCP1 were performed (*n* = 3 per group). **K** The mRNA levels of *Ppar*γ and *Ucp1* in BAT and ingWAT from *Acss2*^*fl/fl*^ or *Acss2*^CKO^ male mice challenged with cold exposure at 4 °C for 16 h were quantitatively analyzed (*n* = 4 per group). **L** The lysates of BAT or ingWAT in mice from *Acss2*^*fl/fl*^ or *Acss2*^CKO^ male mice challenged with cold exposure at 4 °C for 16 h were subjected to western blot with indicated antibodies. The levels of ACSS2, PPARγ and UCP1 were quantitatively analyzed (*n* = 3 per group).

As we found that ACSS2 controlled PPARγ activity via recruiting SIRT1, we then investigated the adipose-specific ACSS2 function in response to cold challenge in mice. We thus generated the adipose-specific *Acss2* knockout mice, *Acss2*^*floxp/floxp,cre+*^ (*Acss2*^*floxp/floxp*^, Adiponectin cre^+^) (*Acss2*^CKO^) using the Cre/Loxp system by crossing *Acss2*^*floxp/floxp*^ and adiponectin-drived Cre-expressing mice (Figs. 5A–C, K, L, S5A). We then placed the *Acss2*^CKO^ mice and their control mice, *Acss2*^*floxp/floxp*^ (*Acss2*^*fl/fl*^) at 4 °C for different time courses, and their temperature and energy expenditure were measured. Under the cold stimulation, we noticed that *Acss2*^CKO^ mice had a lower core body temperature, lower rates of O2 consumption, and decreased heat production (Figs. 5D, E, S5B) compared with the *Acss2*^*fl/fl*^ mice, but no significant differences in respiratory exchange ratio (RER) (Fig. 5F) or food intake (Fig. 5G) were detected. Similar to *Acss2*^−/−^ mice, *Acss2*^CKO^ mice had the similar body temperature, the rates of O2 consumption and the abilities of heat production to *Acss2*^*fl/fl*^ mice in the room temperature (Figs. 5D, E, S5B). Moreover, *Acss2*^CKO^ mice displayed lesser weight reductions of BAT and ingWAT under the cold stimulation compared with *Acss2*^*fl/fl*^ mice (Fig. 5H). Hematoxylin and eosin (H&E) and immunohistochemistry staining of the BAT and ingWAT sections from *Acss2*^CKO^ mice displayed reduced numbers of multilocular, beige-like adipocytes and lower intensity of UCP1 protein signals compared with those in *Acss2*^*fl/fl*^ mice following cold exposure (Fig. 5I, J). Meanwhile, the expressions of UCP1 and PPARγ in BAT and ingWAT from *Acss2*^CKO^ mice were significantly decreased in response to cold stimulation compared with those in *Acss2*^*fl/fl*^ mice (Fig. 5K, L). These data suggested that adipose-specific ACSS2 deficiency impairs thermogenic fat adaptive thermogenesis, including BAT thermogenesis and ingWAT beiging.

When we further injected the adeno-associated virus subtype 9 (AAV9) vectors encoding adiponectin-drived Cre recombinase to specifically knockdown adipose *Acss2* in *Acss2*^*fl/fl*^ mice, this AAV-mediated adipose-specific *Acss2* knockdown mice (*Acss2*^KD^) showed the similar thermogenetic impairments of BAT and ingWAT to *Acss2*^CKO^ mice in the maintain ability of body temperature (Fig. S6A, B), the rates of O2 consumption (Fig. S6C), heat production (Fig. S6D), respiratory exchange ratio (Fig. S6E), food intake (Fig. S6F) and weight reductions of BAT and ingWAT (Fig. S6G). The expressions of UCP1 and PPARγ in BAT and ingWAT from *Acss2*^KD^ mice were also reduced compared with those in the control mice following cold exposure by assays of hematoxylin and eosin (H&E), immunohistochemistry staining, qRT-PCR and western blot (Fig. S6H–K). These data supported that adipose-specific ACSS2 knockdown also caused the adaptive thermogenetic defects of BAT and ingWAT. In conclusion, adipose ACSS2 deficiency impairs PPARγ-UCP1 axis and suppresses the adaptive thermogenesis of BAT and ingWAT after the cold stimulation in mice.

### Adipose ACSS2 over-expression enhances PPARγ-UCP1 axis and the adipose tissues plasticity to combat HFD-induced obesity in mice

**Fig. 6:**
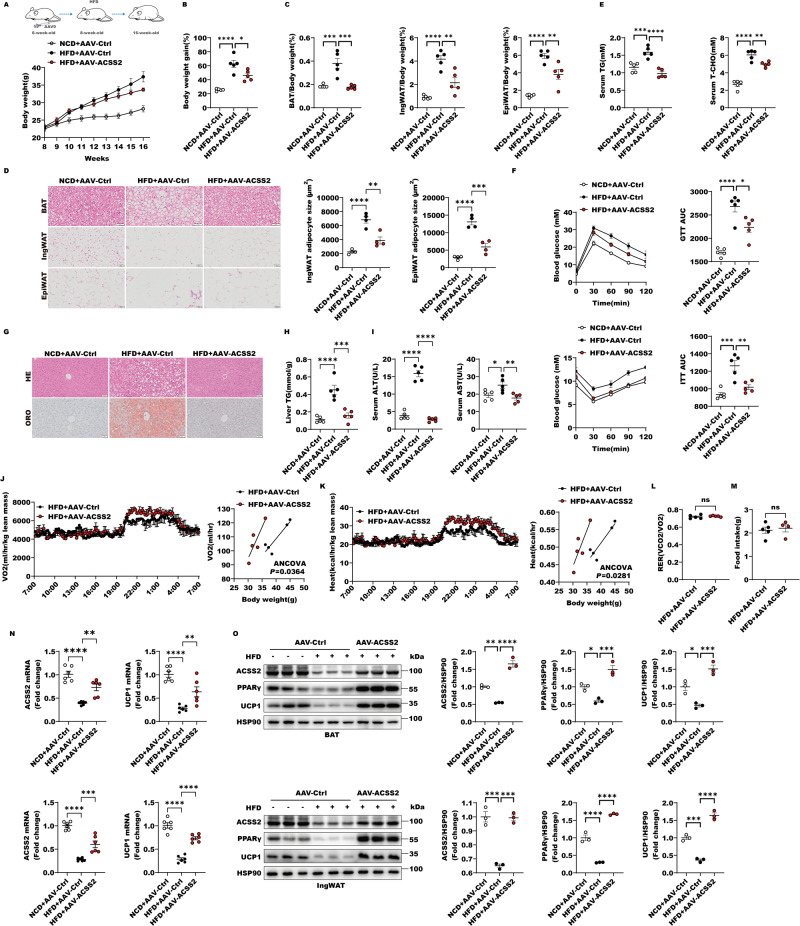
Adipose ACSS2 over-expression enhances PPARγ-UCP1 axis and the adipose tissues plasticity to combat HFD-induced obesity in mice. **A** The changes of mice body weight from NCD + AAV-Ctrl, HFD + AAV-Ctrl and HFD with ACSS2 over expression (HFD + AAV-ACSS2) groups were shown. **B** The quantitative analysis of weight gain percentage from NCD + AAV-Ctrl, HFD + AAV-Ctrl and HFD + AAV-ACSS2 groups was performed (*n* = 5 per group). **C** The quantitative analysis on the weight of BAT, ingWAT and epiWAT in mice from NCD + AAV-Ctrl, HFD + AAV-Ctrl and HFD + AAV-ACSS2 groups was performed (*n* = 5 per group). **D** Representative H&E staining images of BAT, ingWAT and epiWAT from NCD + AAV-ctrl, HFD + AAV-ctrl and HFD + AAV-ACSS2 groups were shown. The average size of adipocytes for each mouse was measured using Image J software based on five different fields of view. This average value was then utilized for statistical analysis (*n* = 4 per group). **E** The levels of TG and TC in serum of NCD + AAV-Ctrl, HFD + AAV-Ctrl and HFD + AAV-ACSS2 groups were determined. (*n* = 5 per group). **F** Glucose tolerance test and insulin tolerance test were performed in mice from NCD + AAV-Ctrl, HFD + AAV-Ctrl and HFD + AAV-ACSS2 groups. The areas under the curve for glucose during GTT or ITT (*n* = 5 per group) were analyzed. **G** Representative H&E and Oil Red O staining images of mice liver from NCD + AAV-Ctrl, HFD + AAV-Ctrl and HFD + AAV-ACSS2 groups were shown (*n* = 4 per group). **H** The levels of TG in livers of NCD + AAV-Ctrl, HFD + AAV-Ctrl and HFD + AAV-ACSS2 groups were measured (*n* = 5 per group). **I** The enzymatic activities of ALT and AST in serum of NCD + AAV-Ctrl, HFD + AAV-Ctrl and HFD + AAV-ACSS2 groups were determined (*n* = 5 per group). **J**, **K** Rates of O2 consumption (**J**) and heat production (**K**) and regression-based analysis of absolute heat production against body weight of HFD + AAV-Ctrl and HFD + AAV-ACSS2 groups were shown (*n* = 5 per group). **L** Respiratory exchange ratio of HFD + AAV-Ctrl and HFD + AAV-ACSS2 mice were shown (*n* = 5 per group). **M** Food intake of HFD + AAV-Ctrl and HFD + AAV-ACSS2 mice were shown (*n* = 5 per group). **N** The mRNA levels of *Acss2* and *Ucp1* in BAT and ingWAT from NCD + AAV-Ctrl, HFD + AAV-Ctrl and HFD + AAV-ACSS2 groups were quantitatively analyzed (*n* = 6 per group). **O** The lysates of BAT or ingWAT in mice from NCD + AAV-Ctrl, HFD + AAV-Ctrl and HFD + AAV-ACSS2 groups were subjected to western blot with indicated antibodies. The levels of ACSS2, PPARγ and UCP1 were quantitatively analyzed (*n* = 3 per group).

To further investigate adipose-specific ACSS2 function, we generated adipose-specific ACSS2 over-expression mice (AAV-ACSS2) to evaluate its phenotype with the high fat diet (HFD)-induced obesity by injected the AAV9 vectors encoding adiponectin-drived ACSS2 over-expression in wild type mice (Fig. 6A). Interestingly, the AAV-ACSS2 mice significantly suppressed body weight gain and fat mass accumulation in ingWAT, epiWAT and BAT compared with the control mice with HFD (Fig. 6A–C). The adipocyte expansions of ingWAT, epiWAT and BAT by H&E stain were also remarkably reduced in HFD-treated AAV-ACSS2 mice (Fig. 6D). Besides, adipose-specific ACSS2 over-expression improved levels of serum triglyceride (TG) and total cholesterol (T-CHO), obesity-related insulin resistance, glucose tolerance (Fig. 6E, F). The abnormal lipid accumulation in liver and impaired activities of alanine transaminase (ALT) and aspartate transaminase (AST) in serum were also greatly recused in HFD-treated AAV-ACSS2 mice (Fig. 6G-I). Moreover, the HFD-treated AAV-ACSS2 mice showed an enhanced rates of O2 consumption (Fig. 6J) and heat production compared with HFD-induced obese mice (Fig. 6K) without differences in respiratory exchange ratio (RER) (Fig. 6L) and food intake (Fig. 6M). Correspondingly, the suppression of PPARγ-UCP1 axis in the obese mice were remarkably reversed by adipose-specific ACSS2 overexpression through the assays of their mRNA and protein level changes by qRT-PCR and western blot (Fig. 6N, O). Taken together, adipose-specific ACSS2 overexpression can enhance the BAT and ingWAT plasticity to protect against obesity and improve its related insulin resistance and liver abnormal lipid accumulation.

### D-mannose rapidly targets adipose tissues to enhance global and nuclear ACSS2 level to combat obesity

**Fig. 7:**
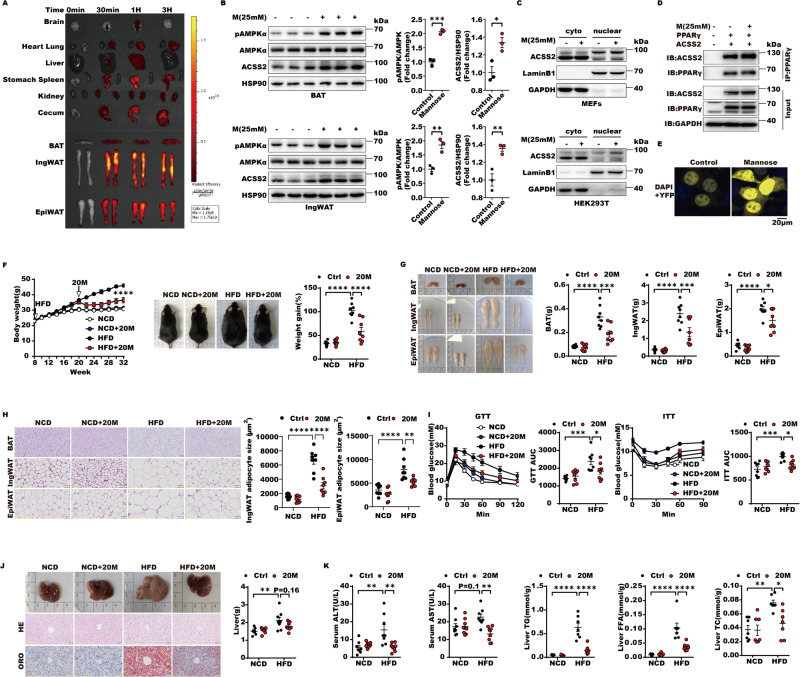
D-mannose rapidly targets adipose tissues to enhance global and nuclear ACSS2 level to combat obesity. **A** The status of D-mannose distribution in various tissues and organs, including brain, heart, lung, liver, stomach, spleen, kidney, cecum and adipose tissues, such as BAT, ingWAT and epiWAT after one single tail vein injection of 125 μL 5 mM FITC-labelled D-mannose (*n* = 2 per group). **B** The tissue lysates of BAT and ingWAT explants from 12-week-old WT mice (*n* = 3 per group) treated with or without 25 mM D-mannose 24 h were subjected to western blot with indicated antibodies. The quantitative analyses of pAMPK and ACSS2 were performed. **C** The levels of cytoplasmic and nuclear ACSS2 in the presence or absence of 25 mM D-mannose in MEFs and HEK293T cells were examined by western blot with indicated bodies (*n* = 2 biological replicates). **D** Representative immunoblot of exogenous IP of PPARγ and ACSS2 with or without 25 mM D-mannose treatment in HEK293T cells (*n* = 2 biological replicates). **E** Representative BiFC images of HEK293T cells transfected with 2 μg plasmid encoding ACSS2 and PPARγ fused to the fluorescent protein fragments in the presence or absence of 25 mM D-mannose indicated in each panel (*n* = 3 biological replicates). **F**–**K** 8-week-old wild type male mice were fed with NCD or HFD for 24 weeks, and provided with regular tap water or 20% (W/V) D-mannose in drinking water at the specified time. **F** The changes of mice body weight from four groups of mice, NCD (mice with normal chow diet), NCD + 20 M (mice with normal chow diet plus 20% D-mannose in oral drinking water), HFD (mice with high fat diet) and HFD + 20 M (mice with high fat diet plus 20% D-mannose in oral drinking water) were shown. The representative images of mice were shown. The percentage of weight gain was statistically calculated (*n* = 8 per group). **G** The changes of BAT, ingWAT and epiWAT from NCD, NCD + 20 M, HFD and HFD + 20 M groups were photographed and their weights were quantitatively analyzed (*n* = 8 per group). **H** Representative H&E staining images of BAT, ingWAT and epiWAT from NCD, NCD + 20 M, HFD and HFD + 20 M groups were shown. The average size of adipocytes for each mouse was measured using Image J software based on five different fields of view. This average value was then utilized for statistical analysis (*n* = 7–8 per group). **I** Glucose tolerance test and insulin tolerance test were performed in mice from NCD, NCD + 20 M, HFD and HFD + 20 M groups. The areas under the curve for glucose during GTT or ITT (*n* = 8 per group) were analyzed. **J** Representative photographs, H&E and Oil Red O staining images of mice liver from NCD, NCD + 20 M, HFD and HFD + 20 M groups. The liver weights per group were quantitatively analyzed (*n* = 8 per group). **K** The enzymatic activities of ALT and AST in serum of NCD, NCD + 20 M, HFD and HFD + 20 M groups were determined. The levels of TG, TC and FFA in livers of these mice were also measured (*n* = 7–8 per group).

Metabolic stress (nutrient deficiency and hypoxia) can induce the expression of ACSS2. Activated AMPK can phosphorylate ACSS2 into the nucleus. D-mannose shares the same transporter as glucose, which inhibits glycolysis and activates AMPK [29, 30]. We speculate that D-mannose may rapidly increase the level of ACSS2 and AMPK-dependent ACSS2 nuclear translocation in highly glucose-utilizing adipose tissues. Interestingly, we found that abundant D-mannose rapidly entered adipose tissues within 30 min after tail vein injection and lasted for 3 h with the highest D-mannose absorption in ingWAT (Figs. 7A, S7A, B). Both the global and nuclear levels of ACSS2 in ingWAT and BAT were both significantly elevated with D-mannose entry to adipose tissues (Fig. 7B, C). Moreover, the interaction of ACSS2 and PPARγ was augmented in response to D-mannose, indicating that PPARγ-UCP1 axis may be activated by D-mannose (Fig. 7D, E). To gain insight into D-mannose effect on adipose tissues plasticity in vivo, we firstly performed oral administration of super-physiological 1%, 10% and 20% D-mannose to HFD-fed mice and found that 20% D-mannose could prevent body weight gain and fat mass accumulation in ingWAT, epiWAT and BAT without influencing water and food intake (Fig. S7C–F). The glucose utilization, insulin sensitivity and liver lipid accumulation were also improved with 20% D-mannose treatment (Fig. S7G, H). Based on this, we used 20% D-mannose in drinking water to treat obese mice with 12-week HFD and found that D-mannose rapidly caused significant body weight loss in obese mice after 2-week D-mannose supplementation (Fig. 7F). Fat mass accumulations in ingWAT, epiWAT and BAT and adipocyte expansions by Haematoxylin and Eosin (H&E) stain were remarkably reduced (Fig. 7G, H). Besides, D-mannose also improved obesity-related insulin resistance, glucose tolerance and liver steatosis (Fig. 7I–K). Collectively, D-mannose can rapidly target adipose tissues to enhance ACSS2 to protect against obesity and improve its related insulin resistance and liver steatosis.

### D-mannose increases adipose tissues energy expenditure to promote ingWAT beiging and BAT thermogenesis

**Fig. 8:**
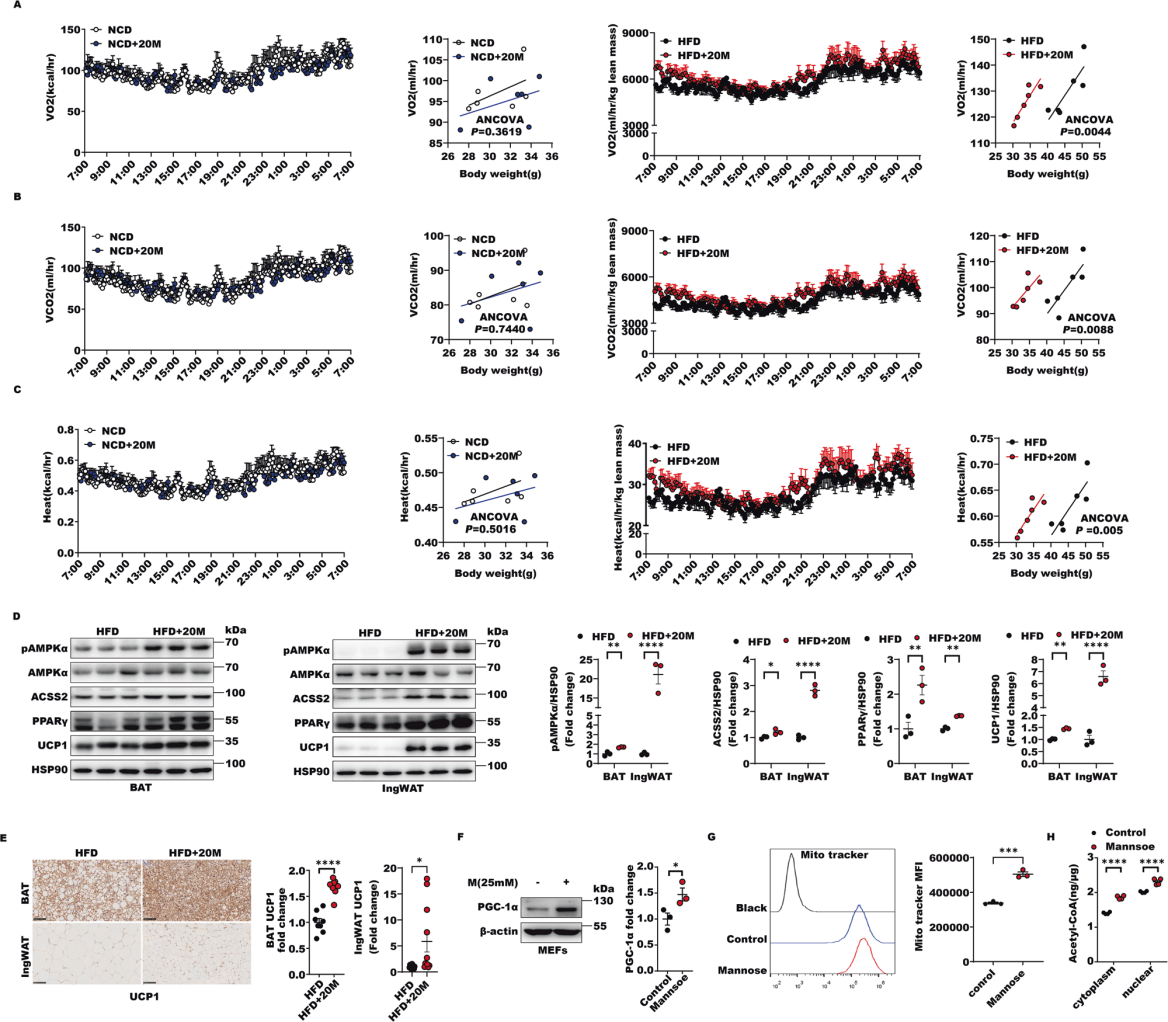
D-mannose increases adipose tissues energy expenditure to promote ingWAT beiging and BAT thermogenesis. **A**–**C** metabolic cage experiments were performed in NCD, NCD + 20 M, HFD and HFD + 20 M groups of mice after 24 weeks intervention. Oxygen consumption (**A**), carbon dioxide generation (**B**) and heat production (**C**) of mice from 4 groups (*n* = 6 per group) were measured and regression-based analysis were shown. **D** The levels of pAMPKα, ACSS2, PPARγ and UCP1 were quantitatively analyzed in the lysates of BAT or ingWAT from HFD and HFD + 20 M groups (*n* = 3 per group). **E** Representative immunohistochemistry images of UCP1 staining of BAT or ingWAT in mice from HFD and HFD + 20 M groups. The signals of UCP1 were quantitatively calculated (*n* = 7–12 per group). **F** The expressions of PGC-1α in response to 25 mM D-mannose in MEFs were evaluated by western blot with anti-PGC-1α antibody and the signal of β-actin was as a control. The quantitative analysis was performed to indicate its levels (*n* = 3 biological replicates). **G** The number of mitochondria in MEFs was examined with or without 25 mM D-mannose by the flow cytometry with the Mito-tracker stain. The intensities of signal of Mito-tracker was quantitatively analyzed (*n* = 3 biological replicates). **H** The levels of acetyl-CoA in the cytoplasm and nucleus extract of HEK293T cells with or without 25 mM D-mannose were determined (*n* = 4 biological replicates).

Since ACSS2 is able to activate SIRT1-PRDM16-PPARγ-UCP1 axis, we attempted to evaluated D-mannose, as a rapid ACSS2 inducer, effect on mice gross metabolism rates. Firstly, D-mannose treated obese mice displayed higher oxygen (O2) consumption and carbon dioxide (CO_2_) production rates during both light and dark cycles compared with HFD-induced obese mice, whereas normal chow diet (NCD) mice were not influenced by D-mannose (Fig. 8A, B). Furthermore, heat generation capacity was also increased by D-mannose treatment in HFD, rather than NCD mice without affecting mice respiratory exchange ratio, food intake and locomotion activity (Figs. 8C, S8A–C). Consistent with higher metabolic rates and heat production, ingWAT beiging and BAT thermogenesis were significantly enhanced by D-mannose with higher levels of ACSS2, PPARγ and UCP1 and AMPK activation in obese mice (Fig. 8D, E). Beige and brown adipocytes are characterized by multilocular lipid droplets, high mitochondrial density and expression of UCP1. Regarding this, we further examined D-mannose function in mitochondria and discovered that the protein levels of the master regulator of mitochondria biogenesis, PGC-1α, and the resulting mitochondria numbers were remarkably increased by D-mannose (Fig. 8F, G). Cellular acetyl-CoA production, which strongly depends on mitochondria, was thus enhanced by D-mannose, suggesting that fatty acid oxidation is activated in response to D-mannose (Fig. 8H). These data showed that D-mannose could facilitate mitochondria biogenesis and increase adipose tissues energy expenditure to promote ingWAT beiging and BAT thermogenesis.

### D-mannose targets adipose ACSS2 to combat obesity

**Fig. 9:**
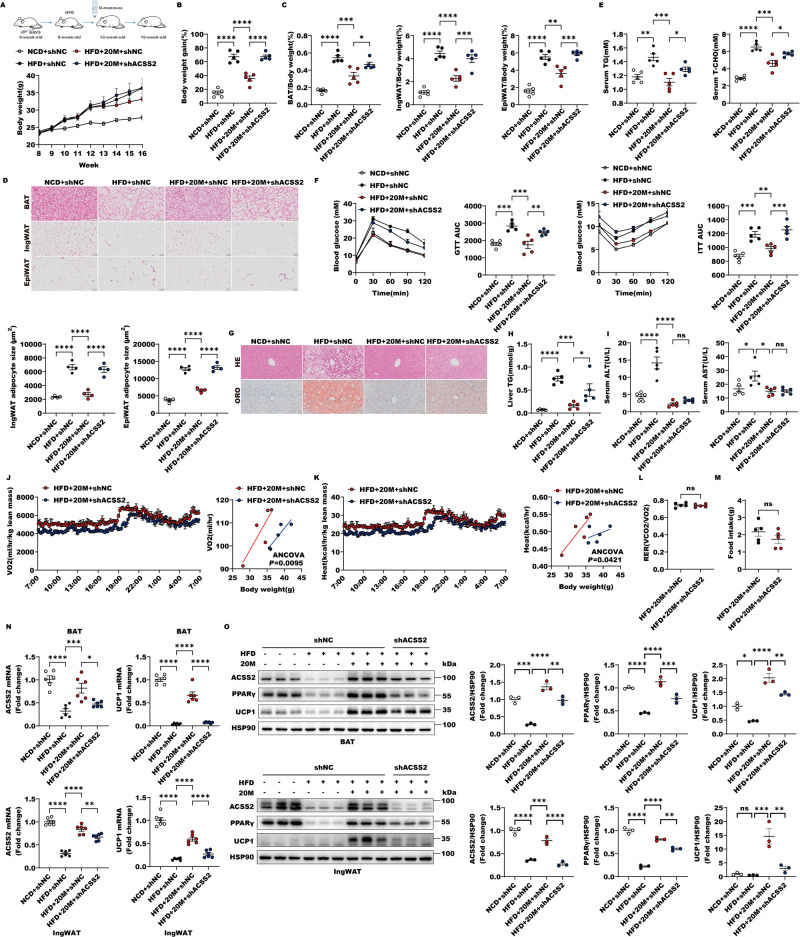
D-mannose targets adipose ACSS2 to combat obesity. **A** The changes of mice body weight from NCD+shNC, HFD+shNC, HFD + 20 M+shNC and HFD + 20 M with ACSS2 down-regulation (HFD + 20 M+shACSS2) groups were shown (*n* = 5 per group). **B** The quantitative analysis of weight gain percentage from NCD+shNC, HFD+shNC, HFD + 20 M + shNC and HFD + 20 M+shACSS2 groups was performed (*n* = 5 per group). **C** The quantitative analysis on the weight of BAT, ingWAT and epiWAT in mice from NCD+shNC, HFD+shNC, HFD + 20 M+shNC and HFD + 20 M + shACSS2 groups was performed (*n* = 5 per group). **D** Representative H&E staining images of BAT, ingWAT and epiWAT from NCD + shNC, HFD + shNC, HFD + 20 M + shNC and HFD + 20 M + shACSS2 groups were shown. The average size of adipocytes for each mouse was measured using Image J software based on five different fields of view. This average value was then utilized for statistical analysis (*n* = 4 per group). **E** The levels of TG and TC in serum of NCD+shNC, HFD+shNC, HFD + 20 M + shNC and HFD + 20 M + shACSS2 groups were determined. (*n* = 5 per group). **F** Glucose tolerance test and insulin tolerance test were performed in mice from NCD+shNC, HFD + shNC, HFD + 20 M + shNC and HFD + 20 M + shACSS2 groups. The areas under the curve for glucose during GTT or ITT (*n* = 5 per group) were analyzed. **G** Representative H&E and Oil Red O staining images of mice liver from NCD + shNC, HFD + shNC, HFD + 20 M + shNC and HFD + 20 M + shACSS2 groups (*n* = 4 per group). **H** The levels of TG in livers of NCD + shNC, HFD + shNC, HFD + 20 M + shNC and HFD + 20 M + shACSS2 groups were measured (*n* = 5 per group). **I** The enzymatic activities of ALT and AST in serum of NCD+shNC, HFD+shNC, HFD + 20 M+shNC and HFD + 20 M + shACSS2 groups were determined (*n* = 5 per group). **J** Rates of O2 consumption and regression-based analysis of absolute O2 consumption against body weight of HFD + 20 M+shNC and HFD + 20 M+shACSS2 groups were shown (*n* = 5 per group). **K** Heat production and regression-based analysis of absolute heat production against body weight of HFD + 20 M + shNC and HFD + 20 M + shACSS2 groups were shown (*n* = 5 per group). **L** Respiratory exchange ratio of HFD + 20 M + shNC and HFD + 20 M+shACSS2 groups were shown (*n* = 5 per group). **M** Food intake of HFD + 20 M+shNC and HFD + 20 M + shACSS2 groups were shown (*n* = 5 per group). **N** The mRNA levels of *Acss2* and *Ucp1* in BAT and ingWAT from NCD + shNC, HFD + shNC, HFD + 20 M + shNC and HFD + 20 M + shACSS2 groups were quantitatively analyzed (*n* = 6 per group). **O** The lysates of BAT or ingWAT in mice from NCD + shNC, HFD + shNC, HFD + 20 M + shNC and HFD + 20 M + shACSS2 groups were subjected to western blot with indicated antibodies. The levels of ACSS2, PPARγ and UCP1 were quantitatively analyzed (*n* = 3 per group).

To investigate whether D-mannose anti-obesity like effect is depended on adipose ACSS2, we injected the AAV9 vectors encoding adiponectin-drived small RNA against *Acss2* to down-regulate adipose ACSS2 expression in D-mannose-administrated HFD-treated mice (Fig. S9A–C). As expected, the decreased ACSS2 expression in adipose significantly abrogated D-mannose-induced reduction in body weight, fat mass content in BAT, ingWAT, and epiWAT (Fig. 9A–C). Histopathology of adipose tissues using H&E stain showed that adipose ACSS2 reduction reversed D-mannose induced smaller adipocyte expansion (Fig. 9D). The improvements in serum triglyceride (TG) and total cholesterol (T-CHO), glucose utilization and insulin resistance by D-mannose administration in obese mice were also reversed by down-regulating adipose ACSS2 (Fig. 9E, F). As to the liver steatosis, the downregulation of adipose ACSS2 partly abolished D-mannose beneficial reductions in fat accumulation of liver, while had no reversion of enzymatic activities of ALT and AST in obese mice (Fig. 9G–I). Furthermore, the higher rates of O2 consumption (Fig. 9J) and heat generation capacity (Fig. 9K) were abolished by adipose ACSS2 downregulation in D-mannose treated obese mice without affecting mice respiratory exchange ratio (Fig. 9L) and food intake (Fig. 9M). To support these observations, adipose ACSS2 downregulation abrogated D-mannose-mediated enhancement in UCP1 and PPARγ expression in ingWAT and BAT of obese mice (Fig. 9N, O). In conclusion, adipose ACSS2 is the target for D-mannose in promoting adipose tissues plasticity and resisting obesity-related disorders.

## Discussion

We here establish that metabolic sensor ACSS2 functions as a tunable switch of PPARγ to maintain its transcriptional activity homeostasis in promoting adipose tissues plasticity. Unexpectedly, the classic acetyl-CoA provider for non-histone and histone acetylation, ACSS2, can promote PPARγ deacetylation, rather than acetylation, and thereafter induce its polyubiquitination and degradation via recruiting SIRT1. In this scenario, ACSS2 positively regulates PPARγ transcriptional activity via facilitating HDAC, Sin3A and NCoR1 departure and SIRT1 recruitment. Followed by this, however, ACSS2 can negatively control its activity by promoting deacetylated PPARγ polyubiquitination and degradation. During this process, P300 primes PPARγ for ACSS2 loading through acetylation modulation while SIRT1 expels ACSS2 from PPARγ to facilitate its translocation to P300 through deacetylation, suggesting that PPARγ acetylation status depends ACSS2 recruitment and departure. As for ACSS2-PPARγ-SIRT1 complex, DBD and hinge subregions of PPARγ has been reported to be responsively flexible to ligand and critical for SIRT1 binding [37, 38]. We demonstrate that PPARγ LBD interacts with ACSS2, suggesting that ACSS2 recruitment may cause PPARγ DBD and hinge domain conformation change to facilitate SIRT1 binding. Furthermore, the recognition site for PPARγ polyubiquitination and degradation is within its LBD and this degradation induced by PPARγ activation is necessary for maintaining PPARγ activity hemostasis and its proper activity [39]. We thus hypothesized that ACSS2 disassociation after SIRT1 recruitment from these regions may facilitate E3 ligase binding with PPAR-γ, thereby promoting PPAR-γ polyubiquitination and degradation. By this manner, ACSS2 thus couples histone acetylation with transcription activator deacetylation in PPARγ switch to control target gene proper expression.

It provides us the details of how co-activators and co-repressors sequential exchange happens on ligand activated PPARγ switch, reflecting the complexity but fine-tuning of gene regulation.

Considering the requirement of appropriate PPARγ switch for adipose tissues function, ACSS2 presents a novel regulator of adipose tissues plasticity via increasing energy expenditure to promote WAT beiging and BAT thermogenesis without influencing adipogenesis. Therefore, it may overcome the side effects of PPARγ overactivation caused by its agonists, like rosiglitazone. Moreover, ACSS2 also have multiple beneficial functions, such as promoting memory formation, alcohol utilization and autophagy induction [24, 25, 40]. At these respects, ACSS2 may be an ideal target of anti-obesity without other side effects. However, ACSS2 has been also evidenced to support tumor growth and may be an important linker in obesity-related myeloma [21, 41]. Therefore, the systematic effects of ACSS2 need further investigation.

Notably, there have been previous reports supporting that PPARγ can either functionally antagonize certain positive transcriptional factors (TFs) to repress transcription or recruit corepressors [42, 43]. SIRT1 functions differently in distinct tissues and can activate PPARγ by deacetylation to transcribe PRDM16 target gene in adipocytes or repressing BACE1 transcription in neuron by disassociating or docking with NCoR1, indicating that binding of SIRT1 and NCoR1 to PPARγ are not always mutually exclusive [36]. Based on the data we collected, it is thus conceivable to hypothesize that ACSS2 may acquire the ability of interfering gene transcription through a unique noncanonical mechanism of PPARγ. As a result, future studies should focus on this topic to profile their separate roles of gene regulation.

Considering that ACSS2 can interact with PPARα, PPARβ and RXRα, it might be a general regulator for NRs. We thus suggest that ACSS2 may have far more protective roles in PPARs and other NRs related diseases including inflammation and neuropsychiatric disorders and neurodegenerative diseases and future work will be required to investigate ACSS2 function in these diseases. Moreover, it is also unclear which genes are governed by ACSS2 and what’s the characteristics of ACSS2 targeted genes. In future, we should pay more attention to other ACSS2 partners in gene regulation to advance our deeper understanding of its physiological role.

In human, glucose level dynamically fluctuates and low physiological glucose can activate AMPK to reprogram cell metabolism [44]. D-mannose, which has a physiological blood concentration less than one-fiftieth of that of glucose, can trigger AMPK as well through perturbing glycolysis and enhance FOA [29, 30]. However, administration of superphysiological amounts of glucose would result in potentially unwanted risk to human health such as metabolic syndrome, type II diabetes, chronic inflammation/autoimmunity and even cancer [45, 46]. In this respect, D-mannose is obviously different from glucose and initiates a complex of ACSS2 and PPARγ to target *Ucp1* transcription, thereby improving obesity and its related disorders, such as glucose utilization, insulin sensitivity and liver steatosis. Also, blood D-mannose, like glucose, can rapidly enter the adipose tissues within 30 min to trigger ACSS2 and enhance WAT beiging and BAT thermogenesis. Importantly, D-mannose seems like a safe agent to combat obesity via reversing the impairments of adipose tissues plasticity in the obese mice while it has no obvious effects in normal diet mice. Considering that D-mannose has diverse beneficial effects, such as anti-tumor, enhancing immune tolerance and anti-inflammation, we propose D-mannose as a rapid inducer of ACSS2 to safely but efficiently treat obesity-related disorders without deleterious effects.

Overall, our findings identify ACSS2 as a novel target to promote WAT beiging and BAT thermogenesis and provide the mechanism of ACSS2 regulation in coupling PPARγ transcriptional activity homeostasis via SIRT1 and histone acetylation. D-mannose functions as a rapid ACSS2 inducer to resist obesity, improve glucose utilization and insulin resistance, and liver steatosis. In addition to D-mannose clinical application in treating cancer, urinary infections, type 1 diabetes and diabetic wounds, we also support the possibility of putting forth D-mannose as a potential therapeutic strategy of obesity and its related disorders via ACSS2.

## Materials and methods

### Mice

All mouse studies were followed by the National Institutes of Health Guide for the Care and Use of Laboratory Animals and approved by the Animal Care and Utilization Committee of Shandong University using the randomization principle with single-blind. *Acss2*^*−/−*^ and the LoxP-flanked *Acss2* mice were generated using the CRISPR-Cas9 system by GemPharmatech Company (Jiangsu, China). We generated the adipose conditional *Acss2* knockout mice by crossing *Acss2*^*floxp/floxp*^ mice with *Adiponectin-Cre* mice (GemPharmatech, Jiangsu, China) and the *Acss2*^*floxp/floxp*^ mice were used for littermate control. The adeno-associated virus subtype 9 (AAV9) vectors encoding adiponectin-drived Cre recombinase, and the adeno-associated virus subtype 9 (AAV9) vectors encoding adiponectin-drived shACSS2 or shNC (Genechem, Shanghai, China) was injected into *Acss2*^*floxp/floxp*^ or wild type mice once through the tail vein to get the adipose-specific ACSS2 knockdown mice (1.5*10^11^vg/100 μl/piece). The shRNA sequence for shACSS2 was: 5′-CAGGAUUGAUGACAUGCUCAA-3′, and the sequence of shNC was: 5′-UUCUCCGAACGUGUCACGUTT-3′. The AAV9 vectors encoding adiponectin-drived ACSS2 (AAV-ACSS2) (Genechem, Shanghai, China) was injected to the wild type mice (1.5*10^11^vg/100 μl/piece) to generate adipose ACSS2 overexpression mice in the HFD-treated mice.

For mouse organ imaging experiments, 8-9-week-old male mice were injected with a single injection of 125 μl of 5 mM FITC-labeled mannose (QY-C-FDG5, QiyueBio, Xian, China). For HFD-induced obesity experiments, 8-9-week-old male mice were fed a chow diet (Beijing keao xieli, Beijing, China) or an 60% kcal high fat diet (TP2330, Trophic Animal Feed, Nantong, China) for 16-24 weeks. For D-mannose administration study, mice were provided with regular tap water or 1% (W/V), 10% (W/V) and 20% (W/V) D-mannose (D-813082, Macklin, Shanghai, China) in drinking water at the specified time.

### Cells

HEK293T cell lines were purchased from Shanghai Cell Bank of Chinese Academy of Sciences (GNHu17, Cell Bank of Type Culture Collection of the Chinese Academy of Sciences, Shanghai, China). Mouse embryonic fibroblasts (MEFs) of C57BL/6 J mice were prepared from E13.5 embryos. All cells were cultured in DMEM medium (11995065, Gibco, USA) with 10% heat-inactivated fetal bovine serum (FBS, 10099-141, Gibco, USA) and 100 IU/mL penicillin and 100 μg/mL streptomycin (PS, 030311B, Biological Industries, Israel) generally. All cells were grown in a humidified cell incubator with an atmosphere of 5% CO2 at 37 °C.

### In vitro beige adipocyte differentiation

Isolation of stromal vascular fraction (SVF) cells from ingWAT was excised from 8-12-week-old male mice, minced into pieces with scissors, and then incubated with digestion buffer containing 1 mg/ml type I collagenase (LS004196, Worthington, USA) and 2% BSA (Sigma-Aldrich) in DMEM/F12 (10565018, Gbico, USA) in a ratio of 5 ml/g tissue at 37 °C for 30 mins, followed by centrifugation at 500 g for 5 min. Pellets containing SVF cells were rinsed three times in PBS and resuspended with DMEM/F12 supplemented with 10% FBS and PS. The SVF cells were cultured in a humidified atmosphere containing 5% CO2/95% air at 37 °C.

Confluent SVF cells were cultured in the induction media containing 0.5 mM 3-isobutyl-1-methylxanthine (IBMX, Sigma), 1 μM dexamethasone (DEX, Sigma), 125 nM indomethacin (IDM, MCE), 850 nM insulin (Novo nordisk), 1 nM 3,3’,5-Triiodo-L-thyronine (T3, Selleck) and 1 μM rosiglitazone (Rosig, Selleck) for 2 days and then in maintenance media containing 850 nM insulin, 1 nM T3 and 1 μM rosiglitazone every other day till Day 6. To activate thermogenic gene expression, rosiglitazone was abandoned from the maintenance medium after four days of beige adipocyte induction, and CL316,243 (10 μM, MCE) were added to the fully differentiated adipocytes (Day 6) for 4 h before collecting cells.

### Bacteria

*Escherichia coli* DH5α and Rossetta (DE3) were grown in LB medium at 37 °C with 200 rpm/min and used for recombinant plasmid cloning and protein expression.

### Quantitative real-time polymerase chain reaction (qRT-PCR)

Total RNA from 1 × 10^6^ cells or tissues was isolated by SPARKeasy cell RNA kit (AC0205, Sparkjade, Shandong, China). After the adjustment of RNA concentration, 1% agarose gel was used to check the RNA quality and amount. One microgram RNA was used for reverse transcription by ReverTra Ace qPCR RT Kit (FSQ-101, Toyobo, Tokyo, Japan). The cDNAs were amplified by UltraSYBR Mixture (CW0957, CWBio, Jiangsu, China) on the Bio-Rad CFX 96 (Bio-Rad, Hercules, CA, USA). Cycle threshold (Ct) values were recorded. Data was normalized using 18 s or β-actin and transformed using the 2^−ΔΔCT^method. The primer sequences are shown in Supplementary Table S6.

### Plasmid construction

The plasmids pCMV-ACSS2 and VN173-Flag-ACSS2 encode full-length human ACSS2 (NM_018677.4). Plasmids of pCMV-PPARγ, PGL3-PPARγ and VN155-HA-PPARγ encodes full-length human PPARγ (NM_001354666.3).The human UCP1 promoter sequences were subcloned to pGL3 basic (E1751, Promega, Madison, MI, USA) to drive luciferase expression. For BiFC assay, full length of human ACSS2 or its N terminal(1-321nt) and C-terminal (322-2103nt) truncations were constructed to pVN173 plasmid. PPARγ and its derivatives ΔLBD (1-752nt) and ΔDBD (1-329nt and 526-1425nt) were subcloned to pVC155. ACSS2 point mutations including E239R, S267A, S273A, S280A, E596A, S659A, T363K, S30A, S30E and PPARγ point mutations including K154Q, K154R, K155Q, K155R, K154/155Q, K154/155R, K238Q, K238R, K263Q and K263R were generated using the KOD-Plus Mutagenesis Kit (F0936K, Toyobo, Tokyo, Japan) according to the manufacturer’s protocol.

### Protein purification and pull-down assay

The recombination protein of His-ACSS2 and glutathione *S*-transferase (GST)-PPARγ was induced by isopropyl β-d-1 thiogalactopyranoside (IPTG, B300845-0005, Sangon Biotech,Shanghai, China) in *Escherichia coli* BL21 (DE3) harboring ACSS2-pET28a or PPARγ-pGEX-4t plasmid. These proteins were purified as described previously [24]. Briefly, BL21 (DE3) cells expressing (His)_6_-ACSS2 or GST-tagged PPARγ were cultured overnight and 2.5 mL of the resulting cultures were transferred to 250 mL fresh LB medium (A507002, Sangon Biotech,Shanghai, China) individually. 1 mM IPTG was added to induce (His)_6_-ACSS2 or GST-tagged PPARγ expression for 20 h at 16 °C when OD_600_ of the culture reached around 0.4. The bacterial cells were collected after centrifuged for 15 min at 4000 rpm at 4 °C and resuspended with lysis buffer (25 mM Tris, 50 mM NaCL, PH = 8.0) or phosphate buffered saline (PBS, B320KJ, BasalMedia, Shanghai, China) before lysis via sonication. For his-ACSS2 affinity purification, cell lysates were loaded onto a Ni-NTA column (70666-4, Sigma-Aldrich, USA) and washed with five column volumes of 30 mM imidazole (A600227, Sangon Biotech, Shanghai, China) to remove contaminated proteins. Finally, the his-ACSS2 protein was eluted by elution buffer (250 mmol/L imidazole, 25 mmol/L Tris, PH = 8.0) and dialyzed to remove imidazole before use. For GST- PPARγ purification, cell lysates were loaded onto a GSTrap HP column (GE Healthcare Life Sciences, UK) and washed with five column volumes of PBS. The subsequent elution and dialysis were performed with 10 mM reduced glutathione to extract GST- PPARγ protein without glutathione. The purification efficiency was examined using SDS-PAGE and Coomassie Brilliant Blue (G-250) staining.

For GST pull-down assay, 200 ng of purified His-ACSS2 was incubated with 100 ng of GST- PPARγ together with glutathione agarose beads (sc-2009, Santa Cruz, USA) in a binding buffer (50 mM Tris-HCl, pH 7.5, 1% Triton X-100, 150 mM NaCl, 1 mM dithiothreitol, 0.5 mM EDTA, 100 μM PMSF, 100 μM leupeptin, 1 μM aprotinin, 100 μM sodium orthovanadate, 100 μM sodium pyrophosphate, and 1 mM sodium fluoride) at 4 °C. The glutathione beads were then washed four times with binding buffer and the bound proteins were boiled with SDS buffer prior to electrophoresis on SDS-polyacrylamide gels.

### Imaging assay

125 μl 5 mM FITC-mannose dissolved in PBS was injected into mice through the tail vein. After 5 min, 30 min, 1 h and 3 h intravenous administration, the mice were sacrificed and dissected, the pictures of the distribution of red fluorescence in tissues were captured by IVIS Lumina II *vivo* imaging system (PerkinElmer, MA, USA) at Translational Medicine Center Facility of Shandong University.

### Glucose tolerance test (GTT) and Insulin tolerance test (ITT)

Mice were fasted for 16 h or not, and glucose (2 g/kg, 608203, Sigma-Aldrich, USA) or insulin (0.75 U/kg, Novo nordisk, Danmark) was intraperitoneally injected into the mice, respectively. The tail capillary blood glucose was measured by a GLS-77 glucometer (YASEE, China) at indicated times or before injection.

### Metabolic measurement

The mice metabolic status was monitored by the Comprehensive Laboratory Animal Monitoring System (CLAMS, USA) with a set of live-in cages for automated, non-invasive and simultaneous monitoring of food and water consumption, horizontal and vertical activity and metabolic performance. All groups of mice from NCD+shNC, HFD+shNC, HFD + M+shNC, HFD + M+shACSS2 and mice of WT, *Acss2*^*−/−*^ or mice of *Acss2*^*fl/fl*^, *Acss2*^CKO^, *Acss2*^KD^ were individually placed in the CLAMS metabolic cages with access to food and water. After one-day acclimation, metabolic parameters including the volume of oxygen consumption (VO_2_) and carbon dioxide generation (VCO_2_), the respiration exchange ratio (RER = VCO_2_/VO_2_) and the caloric (heat) value: [(3.815 + 1.232 × RER) × VO_2_] × 1000/body weight were recorded for 3 days. WT and *Acss2*^−/−^ mice were placed at 4 °C for 24 h, metabolic parameters were recorded. Data were collected every 6–8 min over a 24 h period.

### Biochemical analysis

All kits were purchased from Nanjing Jiancheng Bioengineering Institute. Serum alanine transaminase (ALT), aspartate aminotransferase (AST) were detected using alanine aminotransferase assay Kit and aspartate aminotransferase assay Kit. The triglyceride (TG), total cholesterol (TC) and nonesterified free fatty acids (FFA) assay in liver were detected using triglyceride assay kit, total cholesterol assay kit and nonesterified free fatty acids assay kit.

### Hematoxylin and eosin (H&E) and immunohistochemistry (IHC) staining

H&E staining was carried out as the standard protocol described. Paraffin sections were dewaxed, hydrated, and stained with hematoxylin and eosin, respectively, and then dehydrated and mounted. IHC was performed to measure the expression of UCP1 protein in mouse adipose tissues as described previously [47]. Section was dewaxed and hydrated, followed by antigen retrieval. Endogenous peroxidase was blocked by 3% hydrogen peroxide solution (PV-9000, ZSGB-Bio, Beijing, China). The section was incubated with the blocking goat serum (C0265, Beyotime, Shanghai, China) for 15 min and immunostained with anti-UCP1 antibody (ab10983, Abcam, Cambridge, UK) overnight at 4 °C. After three washes with PBS, the slides were stained with horseradish peroxidase-conjugated anti-rabbit IgG (PV-9000, ZSGB-Bio, Beijing, China) and DAB Chromogenic Kit (ZLI-9018, ZSGB-BIO, Beijing, China), followed by counterstaining with hematoxylin. The full section was scanned by using the Panoramic Scanning Microscope-VS120 (Olympus Life Science, Tokyo, Japan). Cell size and the density of UCP1-positive staining (brownish yellow) was analyzed by Image J.

### Oil red O staining

Fresh frozen fixed liver sections were immersed with Oil Red solution for 8–10 min in the dark, and then differentiated with 60% isopropanol, washed with pure water, counterstained with hematoxylin, and mounted with glycerol gelatin. The full sections were scanned by using the Panoramic Scanning Microscope-VS120 (Olympus Life Science, Tokyo, Japan).

### Adipose tissues explant culture

IngWAT or BAT were cut into small pieces and washed by PBS twice, DMEM one time. Then the explants were cultured with DMEM with 10% FBS and 100 IU/mL penicillin and 100 μg/mL streptomycin (0.1 g tissue/mL) with or without 25 mM D-mannose for 24 h.

### Western blotting and Co-immunoprecipitation

Cells or tissues were lysed with RIPA lysis buffer (R0020, Solarbio, Beijing, China) with 1% protease inhibitor cocktail (B14002, Bimake, USA) and 1% phosphatase inhibitor cocktail (B15001, Bimake, USA). The supernatant was collected after high-speed 12,000 rpm centrifugation for 30 min at 4 °C and protein concentration was determined using a BCA method. 10–30 μg of denatured protein was separated by SDS-PAGE, and then transferred to PVDF membranes (IPVH00010, Millipore, Massachusetts, USA). The membranes were blocked with 5% bovine serum albumin (BSA, V900933, Sigma-Aldrich, USA) in TBS-T (Tris-buffered saline Tween 20) for 2 h and incubated with primary antibodies overnight at 4 °C. Then horseradish peroxidase conjugated secondary antibodies were added for 1 h. After three washes with TBS-T, signals were detected by the eECL Western Blot Kit (69078, Millipore, Massachusetts, USA). All protein signals were collected with different exposure time to make sure the bands were not overexposed and within the linear range to perform quantitative analysis. The band intensity was quantified using the Image J software.

The Nuclear/Cytosol Fractionation Kit was used to extract the cytoplasmic and nuclear proteins from MEFs or HEK293T cells. The cytoplasmic and nuclear extraction were prepared for acetyl-CoA or western blot analysis.

HEK293T cells or tissues were lysed by IP buffer. Cell or tissue extracts were clarified by centrifugation at 12000 rpm, and the supernatants were subjected to immunoprecipitation with the indicated antibodies. After overnight incubation at 4 °C, protein A or G agarose beads were added and left for an additional 3 h with rotation. Then the protein complexes were washed 5 times with IP buffer and then subjected to immunoblot analyses with corresponding antibodies as described previously.

### RNA interference and plasmid transfection

ACSS2 interference (siACSS2), control siRNA (siNC) oligos or plasmids were transfected to cells by using JetPRIME transfection reagent (114-15, Polyplus, France) according to the manufacturer’s protocol. Cells were either subjected to immunofluorescence analysis or lysed for qRT-PCR analysis, SDS-PAGE and western blot analysis.

siACSS2: CAGGAUUGAUGACAUGCUCAA

siNC: sense UUCUCCGAACGUGUCACGUTT

antisense ACGUGACACGUUCGGAGAATT

### Fluorescence resonance energy transfer (FRET) assay

For FRET analysis with EGFP and mCherry as donor-acceptor pair, we constructed various mCherry-N1-ACSS2 and pEGFP-C1-PPARγ fusions. A vector expressing EGFP and mCherry served as a reference vector for negative FRET control measurements. Full-length protein expression of the fusion constructs was confirmed by western blot.

When FRET occurs, the intensity of the donor (EGFP) decreases while the intensity of the acceptor (mCherry) emission increases. After acceptor photobleaching, donor signal would rise due to energy return [48]. Acceptor photobleaching was achieved by scanning a region of interest (ROI) (nucleus) 100 times (scans at 1.6-µs pixel time) using the 488-nm (EGFP) and 561-nm (mCherry) laser line at 100% intensity with LSM 780. Bleaching times per pixel were identical for each experiment. Three donor and acceptor images were taken before and 7–12 images were taken after the acceptor-photobleaching procedure to assess changes in donor and acceptor. FRET efficiency, which presents the distance between donor and acceptor, was calculated as follows when acceptor signals were reduced more than 90%: FRET efficiency (%) = (Donor intensity_post-bleaching_ − Donor intensity_pre-bleaching_) * 100 / Donor intensity_post-bleaching_.

### Bimolecular fluorescent complimentary (BiFC) assay

This assay allows for the rapid visualization of the compartment-specific interactions of a protein complex, and protein-protein interactions can be easily quantified in vivo [49]. BiFC expression plasmids for ACSS2 and PPARγ were constructed by inserting the PCR fragment containing full length ACSS2, PPARγ or their derivatives (primer in Table S7) into pBiFC-VN173 and pBiFC-VC155 (22010, 22011, Addgene, Cambridge, MA, USA) with ClonExpress II One Step Cloning Kit (C112-01, Vazyme, Nanjing, China). The resulting plasmids were transfected into HEK293T cells and co-transfection of pBiFC-bFosVC155 and pBiFC-bJunVN173 was defined as a positive. DAPI (S36964, Invitrogen, USA) stain was indicated as cellular nuclear and fluorescence could be detected at 561 nm excitation wavelength after 24 h by LSM780 with a 63× Plan-Apochromat objective and analyzed using ZEN lite 2012 software package.

### Luciferase assay

Luciferase activity was measured with a dual luciferase assay system. The Luciferase reporter vector PGL3 basic and its derivatives with or without human (−3000 - 0, 3000 bp) *Ucp1* promoter region were transiently transfected into the HEK293T cells in the presence of PPARγ and PRDM16 alone or together with ACSS2. 24 h after transfection, luciferase activity was measured with a dual luciferase assay system (DL101-01, Vazyme, Nanjing, China) and the readout was determined using a microplate luminometer (Centro LB 960; Berthold, Wildbad, Germany). Data were analyzed by GraphPad Prism 7. Three independent experiments have carried out for biological replicates.

### Flow cytometry

To assess mitochondria changes, followed by D-mannose treatment, MEFs were incubated with a mitochondrial stain (Mito Tracker Red FM, 50 nM, Ex = 644 nm, Em = 665 nm) at 37 °C for 30 min and then washed with PBS twice. Copper mesh filtrated cells, and CytoFLEX S (BECKMAN, USA) was used for flow cytometry.

### Quantification analysis

The specific western band signals were quantificationally analyzed by Image J. All image statistical analyses were performed using Image J Software and Zeiss Auto-measure software. Nuclear range were circled and the fluorescence intensity of each pixel was calculated by Zeiss Auto-measure software in FRET experiment. More than 50 cells were calculated.

### Statistical analysis

Mean values, standard error of mean, and statistical significance were analyzed by GraphPad Prism 7. Differences between groups were analyzed with the Student’s *t* test (unpaired), one-way or two-way ANOVA followed by the Tukey’s test (for multiple comparisons). *P* values < 0.05 was considered to be statistically significant; *P < 0.05, **P < 0.01, ***P < 0.001, ****P < 0.0001. Results are presented followed by at least three independent experiments of biological replicates.

### Supplementary information

KEY RESOURCES TABLE

Supplemental information merge

Original Data File

## Data Availability

Data from this study are available on request.
