# Supplementary material for: ACSS2 controls PPARγ activity homeostasis to potentiate adipose-tissue plasticity
Source: Cell Death Differ. 2024 Feb 8;31(4):479–96. doi: 10.1038/s41418-024-01262-0 (PMC11043345; doi:10.1038/s41418-024-01262-0)
Supplement: Supplementary file 1 — KEY RESOURCES TABLE [file 41418_2024_1262_MOESM1_ESM.docx]

**KEY RESOURCES TABLE**

| REAGENT or RESOURCE | SOURCE | IDENTIFIER |
| --- | --- | --- |
| Antibodies |  |  |
| Anti-PRDM16, dil:1:1000 | Abcam | ab106410 |
| Anti-UCP1 (for WB dil:1:1000 for WAT;1:2000 for BAT)(for IHC dil：1:250 for WAT;1:500 for BAT) | Abcam | ab10983 |
| Anti-PPARα, dil:1:1000 | Abcam | ab215270 |
| Anti-PPARβ, dil:1:1000 | Abcam | ab23673 |
| Anti-Sin3A, dil:1:1000 | Abcam | ab3479 |
| Anti-Ubiquitin, dil:1:1000 | CST | 20326S |
| Anti-PGC-1α, dil:1:1000 | CST | 2178S |
| Anti-PPARγ (for IP dil:1:200) | CST | 2435S |
| Anti-Phospho-AMPKα (Thr172), dil:1:1000 | CST | 2535S |
| Anti-GST, dil:1:1000 | CST | 2624S |
| Anti-ACSS2, dil:1:1000 | CST | 3658S |
| Anti-NcoR1, dil:1:1000 | CST | 5948S |
| Anti-C/EBPα, dil:1:1000 | CST | 8178S |
| Anti-NF-κB p65 (D14E12), dil:1:1000 | CST | 8242S |
| Anti-Acetylated-Lysine, dil:1:1000 | CST | 9441S |
| Anti-Sirt1, dil:1:1000 | CST | 9475S |
| Anti-HA, dil:1:1000 | Proteintech | 51064-2-AP |
| Anti-Lamin B1, dil:1:1000 | Proteintech | 66095-1-Ig |
| Anti-P300, dil:1:1000 | Santa Cruz | sc-32244 |
| Anti-HDAC (A-3), dil:1:1000 | Santa Cruz | sc-376957 |
| Anti-ACSS2, dil:1:1000 | Santa Cruz | sc-398559 |
| Anti-ACLY, dil:1:1000 | Santa Cruz | sc-517267 |
| Anti-PPARγ (for WB dil:1:1000) | Santa Cruz | sc-7273 |
| Anti-Flag, dil:1:1000 | Sigma-Aldrich | F7425 |
| Anti-GAPDH, dil:1:2000 | ZSGB-BIO | TA-08 |
| Anti-HSP90, dil:1:2000 | ZSGB-BIO | TA-12 |
| Goat anti-rabbit IgG-HRP, dil: 1:2000 | Jackson ImmunoResearch Labs | 111035144 |
| Goat anti-mouse IgG-HRP, dil: 1:2000 | Jackson ImmunoResearch Labs | 115035146 |
| Chemicals and inhibitors |  |  |
| Anti-HA magnetic beads | Bimake | B26201 |
| Anti-Flag magnetic beads | Bimake | B26101 |
| Protease inhibitor cocktail | Bimake | B14002 |
| Phosphatase inhibitor cocktail | Bimake | B15001 |
| MitoTracker Deep Red FM | Invitrogen | M22426 |
| DAPI | Invitrogen | Cat#S36964 |
| D-Mannose | Macklin | D-813082 |
| Insulin | Novo nordisk | N/A |
| FITC-D-Mannose | QiyueBio | QY-C-FDG5 |
| GST-Sefinose(TM) Resin 4FF(Settled Resin) | Sangon Biotech | C600031 |
| IPTG | Sangon Biotech | B300845-0005 |
| Protein A/G-Sepharose | Santa Cruz | sc-2003 |
| Glutathione-Agarose | Santa Cruz | sc-2009 |
| Rosiglitazone | Selleck | S2556 |
| GW9662 | Selleck | S2915 |
| ACSS2 inhibitor | Selleck | S8588 |
| CL316243 | MCE | HY-116771A |
| Type I collagenase | Worthington | LS004196 |
| Indomethacin (IDM) | MCE | HY-14397 |
| 3,3',5-Triiodo-L-thyronine (T3) | Selleck | S5726 |
| Oil Red O | Servicebio | G1015 |
| Anti-His magnetic beads | Sigma-Aldrich | H9914 |
| Streptavidin | Sigma-Aldrich | S1638 |
| Ni-NTA His bind | Sigma-Aldrich | 70666-4 |
| Phenylmethylsulfonyl fluoride (PMSF) | Sigma-Aldrich | Cat#52332 |
| D-Glucose | Sigma-Aldrich | Cat#608203 |
| 3-Isobutyl-1-methylxanthine (IBMX) | Sigma-Aldrich | Cat#I5879 |
| Dexamethasone | Sigma-Aldrich | Cat#D4902 |
| Critical Commercial Assays |  |  |
| Mouse acetyl-CoA ELISA assay kit | Jianglai Bio | Cat#JL32779 |
| Nonesterified Free fatty acids assay kit | Nanjing Jiancheng | A042-2-1 |
| Triglyceride assay kit | Nanjing Jiancheng | A110-1-1 |
| Total cholesterol assay kit | Nanjing Jiancheng | A111-1-1 |
| BacTiter Glo kit | Promega | Cat#G8231 |
| The Nuclear/Cytosol Fractionation Kit | Thermo | Cat#78833 |
| The BCA Protein Assay Kit | Thermo | Cat#23225 |
| ClonExpress II One Step Cloning Kit | Vazyme | Cat#C112-01 |
| Dual luciferase assay system | Vazyme | Cat#DL101-01 |
| Universal two-step assay kit | ZSGB-Bio | PV-9000 |
| DAB Chromogenic Kit | ZSGB-BIO | ZLI-9018 |
| Experimental Models: Cell Lines |  |  |
| Cell line: HEK293T | Shanghai Cell Bank of Chinese Aca- demy of Sciences | GNHu17 |
| Experimental Models: Organism/strains |  |  |
| Mouse: C57BL/6J | Charles River | N/A |
| Mouse: *Acss2*^-/-^ *, Acss2*^flox/flox^ , *Adipoq-cre* | GemPharmatech | N/A |
| Bacteria: *Escherichia coli* BL21(DE3) | Zhuangmeng Bio | ZK202 |
| Bacteria: *Escherichia coli* DH5α | Zhuangmeng Bio | ZK206 |
| Oligonucleotides |  |  |
| Primers used for quantitative PCR, see Table S6 | This study | N/A |
| Primers used for plasmids construction, see Table S7 | This study | N/A |
| Recombinant DNA |  |  |
| PPRE X3-TK-luc | addgene | 1015 |
| pBiFC-VN173 | addgene | 22010 |
| pBiFC-VC155 | addgene | 22011 |
| pBiFC-bJunVN173 | addgene | 22012 |
| pBiFC-bFosVC155 | addgene | 22013 |
| PGEX-4T | Miaoling Bio | P0001 |
| PET28a | Miaoling Bio | P0023 |
| pEGFP-C1 | Miaoling Bio | P0134 |
| pmCherry-N1 | Miaoling Bio | P0475 |
| pECMV-P300-myc | Miaoling Bio | P0691 |
| pCMV-RXRA-Tag 2B | Miaoling Bio | P10927 |
| pEnCMV-PPARD(human)-3×FLAG | Miaoling Bio | P18449 |
| pEnCMV-PPARA(human)-3×FLAG | Miaoling Bio | P18450 |
| pEnCMV-SIRT1(human)-3×HA | Miaoling Bio | P2135 |
| pCMV-SPORT6-Creb1 | Miaoling Bio | P3655 |
| pCMV-PRDM16(mouse)-3×FLAG-SV40-Neo | Miaoling Bio | P38414 |
| pECMV-CEBPβ-m-FLAG | Miaoling Bio | P5635 |
| pGL3 basic | Promega | Cat#E1751 |
| pBiFC-VC155-PPARγ | This study | N/A |
| pBiFC-VN173-ACSS2 | This study | N/A |
| PGL3-UCP1 promoter-Luciferase (Human) | This study | N/A |
| PCMV-ACSS2 | This study | N/A |
| PCMV-PPARγ | This study | N/A |
| PET28a-ACSS2 | This study | N/A |
| PGEX-4T-PPARγ | This study | N/A |
| pmCherry-N1-ACSS2 | This study | N/A |
| pEGFP-C1-PPARγ | This study | N/A |
| PGL3-PPARγ | This study | N/A |
| pCMV-MDM2(human)-3×FLAG-Neo | Miaoling Bio | P41596 |
| pCMV-NEDD4(human)-3×HA-SV40-Neo | Miaoling Bio | P36058 |
| pECMV-3×FLAG-TRIM25 | Miaoling Bio | P3543 |
| pCMV-TRIM27(human)-3×FLAG-Neo | Miaoling Bio | P50192 |
| pCMV-STUB1(human)-3×FLAG-Neo | Miaoling Bio | P41623 |
| pENTER-MKRN1 | Weizhen Bio | CH899109 |
| pcDNA3.1-3×FLAG-C-SIAH2 | Fenghui Bio | NM-005067 |
| Software |  |  |
| CytExpert | Beckman Coulter | https://www.mybeckman.cn/flow-cytometry/research-flow-cytometers/cytoflex/software |
| FlowJo | Bioscienses | https://www.bdbiosciences.com/zh-cn/products/software/flowjo-v10-software |
| Graphpad Prism 9 | Graphpad | https://www.graphpad.com/ |
| OlyVIA | Olympus | https://olyvia.software.informer.com/ |
| Living Image | PerkinElmer | https://www.perkinelmer.com.cn/ |
| Zeiss Zen | Zeiss | https://www.zeiss.com |
| Image J |  | https://imagej.en.softonic.com |
| Other |  |  |
| 100μm cell strainer | BIOFIL | CSS013100 |
| 0.22μm filter | Millipore | SLGP033N |
| PVDF membrane | Millipore | IPVH00010 |
| Mouse high fat diet | Trophic Animal Feed High-Tech Co | TP23300 |
| Mouse normal chow | Beijing Keao Xieli | N/A |
