# Supplementary material for: ACSS2 controls PPARγ activity homeostasis to potentiate adipose-tissue plasticity
Source: Cell Death Differ. 2024 Feb 8;31(4):479–96. doi: 10.1038/s41418-024-01262-0 (PMC11043345; doi:10.1038/s41418-024-01262-0)
Supplement: Supplementary file 2 — Supplemental information merge [file 41418_2024_1262_MOESM2_ESM.pdf]

SUPPLEMENTAL INFORMATION

Figure S1

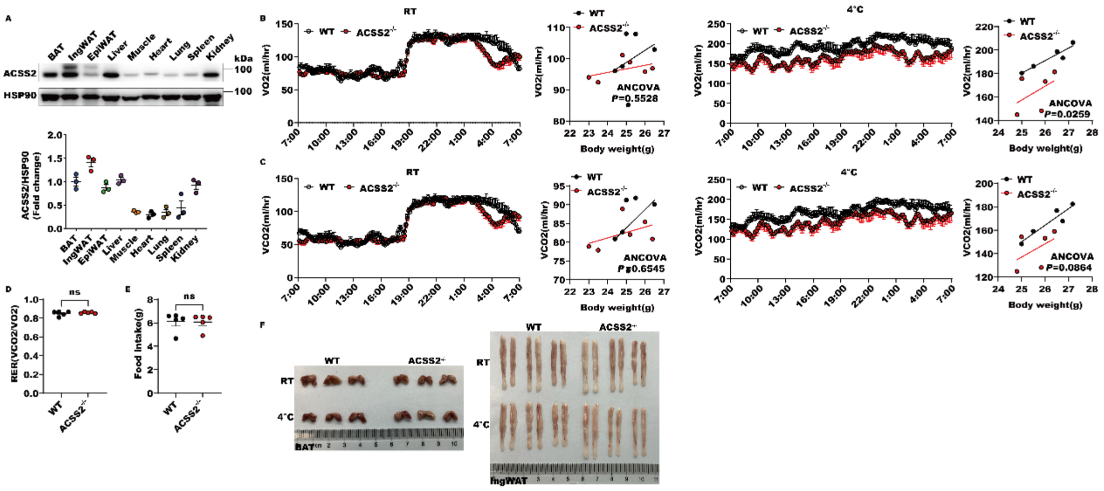

Figure S1. Adipose ACSS2 promotes WAT beiging and BAT thermogenesis

(A) The tissue lysates of various tissues and organs, including BAT, ingWAT and epiWAT, liver, muscle, heart, lung, spleen and kidney were subjected to western blot with indicated antibodies. The quantitative analyses of ACSS2 were performed (n = 3 biological replicates).

(B-E) The metabolic cage experiments were performed in 8-12-week-old *Acss2*$^{-/-}$ male mice or wild type male mice (WT) with or without cold challenge for 24 hours.

(B) Oxygen consumption (VO2) rates and regression-based analysis of absolute VO2 against body weight of WT mice and *Acss2*$^{-/-}$ mice were shown (n = 5 per group).

(C) Carbon dioxide consumption (VCO2) rates and regression-based analysis of absolute VCO2 against body weight of WT mice and *Acss2*$^{-/-}$ mice were shown (n = 5 per group).

(D) Respiratory exchange ratio (RER) of WT mice and *Acss2*$^{-/-}$ mice challenged with cold exposure at 4 °C for 24 h were shown (n = 5 per group).

(E) Food intake of WT mice and *Acss2*$^{-/-}$ mice challenged with cold exposure at 4 °C for 24 h were shown (n = 5 per group).

(F) Representative images of BAT and ingWAT from 6-8-week-old wild type or *Acss2*$^{-/-}$ male mice challenged with or without cold exposure at 4 °C for 16 h were shown (n = 3 per group).

Figure S2

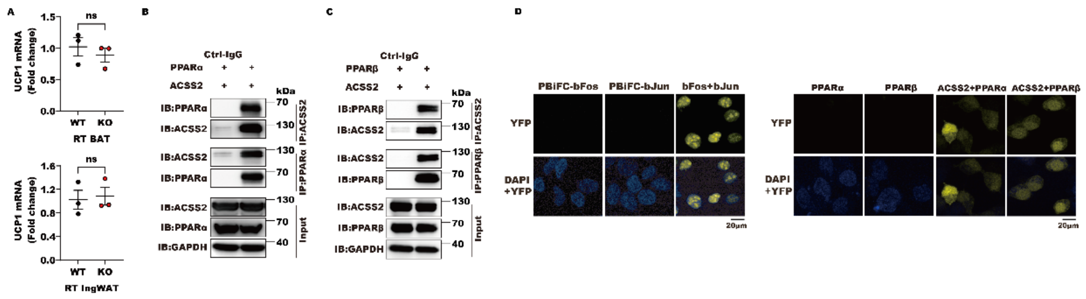

Figure S2. ACSS2 is also a co-activator of PPARα and PPARβ

(A) The mRNA levels of *Ucp1* and *Acss2* in BAT and ingWAT from 6-8-week-old wild type or *Acss2*$^{-/-}$ male mice in room temperature were shown (n = 3 per group).

(B-C) Co-IP of FLAG-tagged ACSS2 and HA-tagged PPARα (A) or PPARβ (B) in HEK293T cells (n = 2 biological replicates).

(D) Fluorescent images of HEK293T cells transfected with 2 μg plasmid encoding bJun or bFos fused to the fluorescent protein fragments indicated in each panel. DAPI stain demonstrated nuclear locus. This pair was designed as the positive control for BiFC assay. The intensity YFP signal indicates the amounts and localization of BiFC complex (bJun-bFos). For PPARα or PPARβ interactions with ACSS2, HEK293T cells transfected with 2 μg plasmid encoding ACSS2 and PPARα or PPARβ alone or together fused to the fluorescent protein fragments indicated in each panel. The intensity YFP signal indicates the amounts and localization of BiFC complex (ACSS2-PPARα/β) (n = 2 biological replicates).

Figure S3

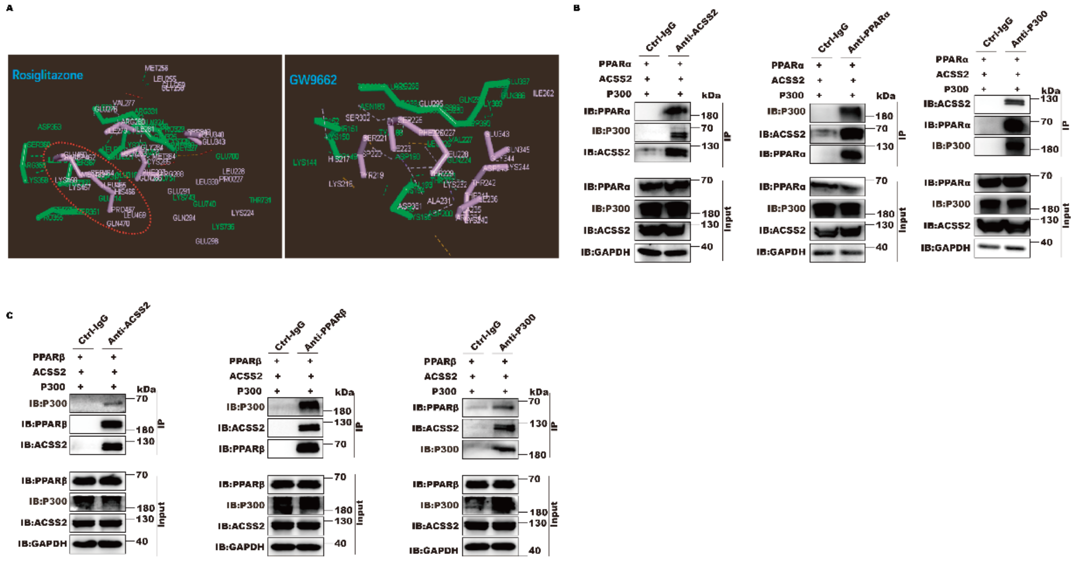

Figure S3. ACSS2 binds PPARγ in a ligand-dependent manner

(A) The amino acid composition at the interaction face between ACSS2 and PPARγ in the presence of rosiglitazone or GW9662 by protein-protein docking analysis. The colored green stick shows ACSS2 amino acid composition to bind PPARγ; The colored purple presents PPARγ amino acid composition to interact with ACSS2. The red dashed ellipse highlights that ACSS2 can characteristically bind PPARγ C terminal (containing flexible region between helix 11 (439-460) and helix 12 and helix 12) in response to rosiglitazone.

(B) CoIP of FLAG-tagged ACSS2, HA-tagged PPARα and HA-P300 in HEK293T cells (n = 2 biological replicates).

(C) CoIP of FLAG-tagged ACSS2, HA-tagged PPARβ and HA-P300 in HEK293T cells (n = 2 biological replicates).

Figure S4

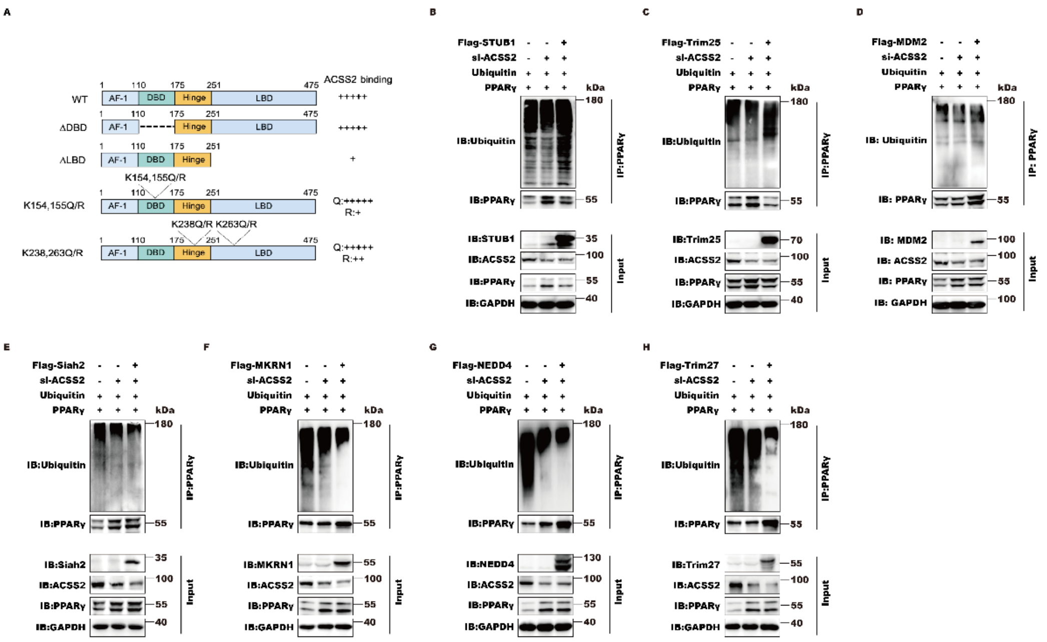

Figure S4. ACSS2 regulates PPARγ transcriptional activity by SIRT1

(A) Schematic diagrams of PPARγ full length and internal and acetylation mutants.

(B) Representative immunoblot the changes of PPARγ ubiquitination following transfection with PPARγ, Ubiquitin and STUB1 with or without the small RNA interference against ACSS2 in HEK293T cells (n = 2 biological replicates).

(C) Representative immunoblot the changes of PPARγ ubiquitination following transfection with PPARγ, Ubiquitin and Trim25 with or without the small RNA interference against ACSS2 in HEK293T cells (n = 2 biological replicates).

(D) Representative immunoblot the changes of PPARγ ubiquitination following transfection with PPARγ, Ubiquitin and MDM2 with or without the small RNA interference against ACSS2 in HEK293T cells (n = 2 biological replicates).

(E) Representative immunoblot the changes of PPARγ ubiquitination following transfection with PPARγ, Ubiquitin and Siah2 with or without the small RNA

interference against ACSS2 in HEK293T cells (n = 2 biological replicates).

(F) Representative immunoblot the changes of PPARγ ubiquitination following transfection with PPARγ, Ubiquitin and MKRN1 with or without the small RNA interference against ACSS2 in HEK293T cells (n = 2 biological replicates).

(G) Representative immunoblot the changes of PPARγ ubiquitination following transfection with PPARγ, Ubiquitin and NEDD4 with or without the small RNA interference against ACSS2 in HEK293T cells (n = 2 biological replicates).

(H) Representative immunoblot the changes of PPARγ ubiquitination following transfection with PPARγ, Ubiquitin and Trim27 with or without the small RNA interference against ACSS2 in HEK293T cells (n = 2 biological replicates).

Figure S5

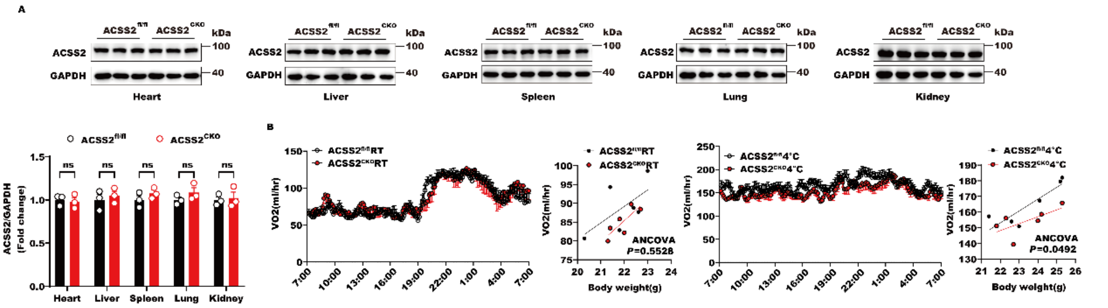

Figure S5. Adipose-specific ACSS2 knockout mice displayed the impairments in the adaptive thermogenesis of adipose tissues under the cold stimulation in mice

(A) The protein levels of ACSS2 in liver, heart, spleen, lung and kidney from *Acss2*$^{fl/fl}$ or *Acss2*^{CKO} mice were quantitatively analyzed by western blot (n = 3 per group).

(B) The metabolic cage experiments were performed in 6-8-week-old *Acss2*$^{fl/fl}$ or *Acss2*^{CKO} mice exposed to cold stimulation. VO2 rates and regression-based analysis of absolute VO2 against body weight of *Acss2*$^{fl/fl}$ or *Acss2*^{CKO} mice were shown (n = 6 per group).

Figure S6

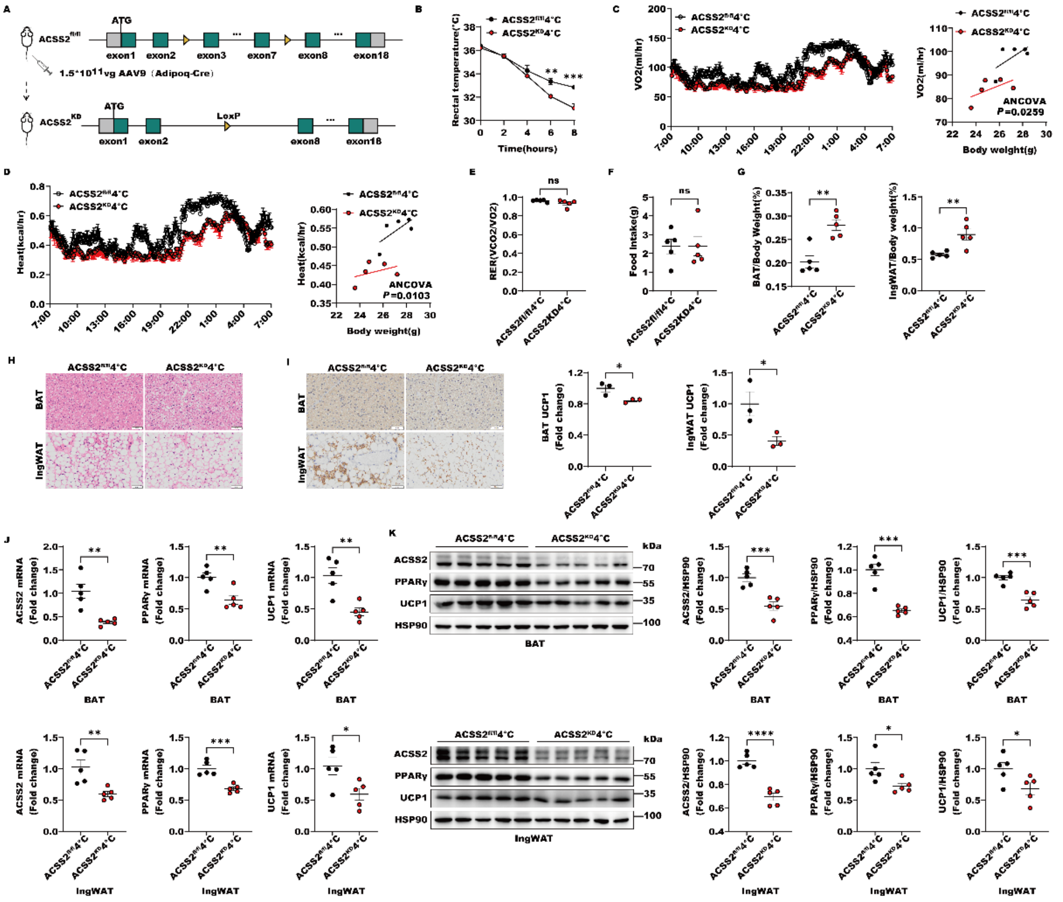

Figure S6. Adipose-specific ACSS2 knockdown impairs the adaptive thermogenesis of AT under the cold stimulation in mice

(A) The adipose-specific *Acss2* knockdown mice (*Acss2*^{KD}) were generated by injecting the adeno-associated virus subtype 9 (AAV9) vectors encoding adiponectin-drived Cre recombinase to specifically knockdown adipose *Acss2* in *Acss2*$^{fl/fl}$ mice.

(B) The rectal temperature of 6-8-week-old *Acss2*$^{fl/fl}$ or *Acss2*^{KD} male mice exposed to cold (4 °C for 8 hours) were shown (n = 5 per group).

(C-F) The metabolic cage experiments were performed in 6-8-week-old *Acss2*^{KD} or *Acss2*$^{fl/fl}$ male mice with or without cold challenge for 24 hours.

(C) VO2 rates and regression-based analysis of absolute VO2 against body weight of

Acss2^{KD} or *Acss2*$^{fl/fl}$ male mice were shown (n = 5 per group).

(D) Heat production and regression-based analysis of absolute heat against body weight of *Acss2*^{KD} or *Acss2*$^{fl/fl}$ male mice were shown (n = 5 per group).

(E) RER of *Acss2*^{KD} or *Acss2*$^{fl/fl}$ male mice challenged with cold exposure at 4 °C for 24 h were shown (n = 5 per group).

(F) Food intake of *Acss2*^{KD} or *Acss2*$^{fl/fl}$ male mice challenged with cold exposure at 4 °C for 24 h were shown (n = 5 per group).

(G) The quantitative analysis on the weight of BAT and ingWAT in mice from *Acss2*$^{fl/fl}$ and *Acss2*^{KD} challenged with cold exposure at 4 °C for 16 h was performed (n = 4 per group).

(H-I) Representative H&E (H) or immunohistochemistry for UCP1 (I) of BAT and ingWAT from 6-8-week-old *Acss2*$^{fl/fl}$ or *Acss2*^{KD} male mice challenged with cold exposure at 4 °C for 16 h were shown, and the quantitative analyses of UCP1 were performed (n = 5 per group).

(J) The mRNA of *Acss2, Pparγ* and *Ucp1* in BAT or ingWAT from *Acss2*$^{fl/fl}$ or *Acss2*^{KD} male mice challenged with cold exposure at 4 °C for 16 h were quantitatively analyzed (n = 5 per group).

(K) The protein expression of ACSS2, PPARγ and UCP1 in BAT or ingWAT from *Acss2*$^{fl/fl}$ or *Acss2*^{KD} male mice challenged with cold exposure at 4 °C for 16 h were quantitatively analyzed (n = 5 per group).

Figure S7

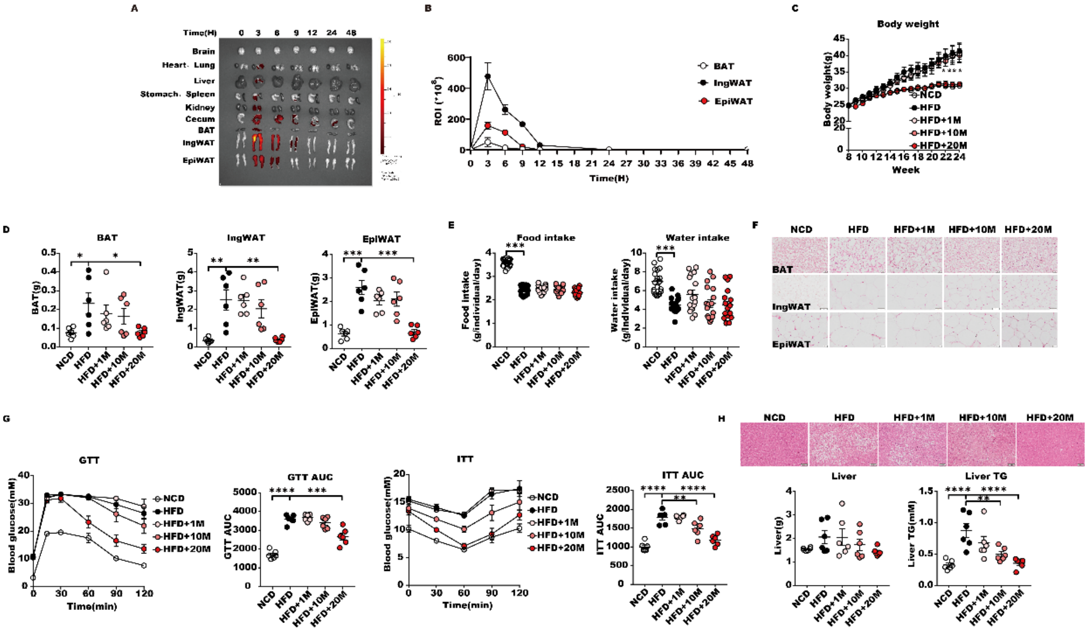

Figure S7. Oral 20% D-mannose can prevent HFD-induced obesity in mice

(A) The changes of D-mannose distribution in various tissues and organs, including brain, heart, lung, liver, stomach, spleen, kidney, cecum and adipose tissues, such as BAT, ingWAT and epiWAT after one single tail vein injection of 125 μL 5 mM FITC-labelled D-mannose in different time points (n = 3 per group).

(B) The quantitative analysis of D-mannose distribution in various adipose tissues such as BAT, ingWAT and epiWAT after one single tail vein injection of 125 μL 5 mM FITC-labelled D-mannose in different time points (n = 3 per group).

(C) The changes of mice body weight (n=6 per group) in response to 1%, 10% and 20% D-mannose with or without high fat diet for 24 weeks. The percentage of weight gain was statistically calculated (n = 6 per group).

(D) The changes of BAT, ingWAT and epiWAT from five groups of mice, NCD (mice with normal chow diet), HFD (mice with high fat diet), HFD+1M (mice with high fat

diet plus 1 % D-mannose in oral drinking water), HFD+10M (mice with high fat diet plus 10 % D-mannose in oral drinking water) and HFD+20M (mice with high fat diet plus 20% D-mannose in oral drinking water), were quantitatively analyzed (n = 6 per group).

(E) The food and water intake status of mice with or without high fat diet in response to 1%, 10% and 20% D-mannose was recorded.

(F) Representative H&E staining images of BAT, ingWAT and epiWAT from mice in groups of NCD, HFD, HFD+1M, HFD+10M and HFD+20M.

(G) Glucose tolerance test and insulin tolerance test were performed in mice from NCD, HFD, HFD+1M, HFD+10M and HFD+20M groups. The areas under the curve for glucose during GTT or ITT (n=6 per group) were analyzed.

(H) Representative H&E staining images of mice liver from NCD, HFD, HFD+1M, HFD+10M and HFD+20M groups. The liver weights and TG levels were quantitatively analyzed (n=6 per group).

Figure S8

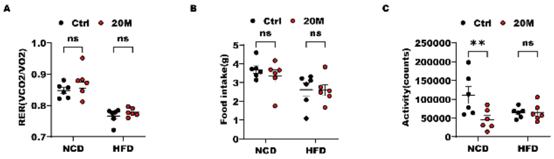

Figure S8. Oral 20% D-mannose doesn't influence mice RER, food intake and activity

(A-C) The RER (A), food intake (B) and activities (C) of mice with or without high fat diet in response to 20% D-mannose were statically analyzed (n=6 per group).

Figure S9

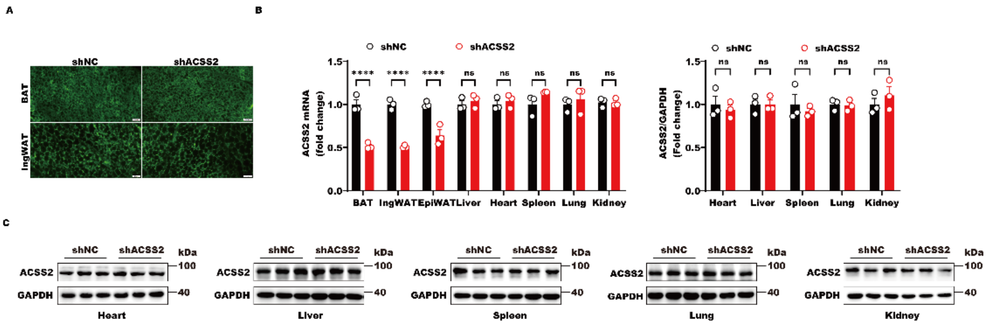

Figure S9. D-mannose targets adipose ACSS2 to combat obesity

(A) The detection of green fluorescence in the BAT and ingWAT of mice after injecting the GFP-AAV9 vectors encoding adiponectin-drived shRNA against *Acss2* through immunofluorescence.

(B) The mRNA levels of *Acss2* in BAT, ingWAT, epiWAT, Liver, Heart, Spleen, Lung and Kidney from shNC or shACSS2 mice challenged with cold exposure at 4 °C for 16 h were quantitatively analyzed (n = 3 per group).

(C) The protein levels of ACSS2 in liver, heart, spleen, lung and kidney from shNC or shACSS2 mice were quantitatively analyzed by western blot (n = 3 per group).

KEY RESOURCES TABLE

REAGENT or RESOURCE	SOURCE	IDENTIFIER
Antibodies		
Anti-PRDM16, dil:1:1000	Abcam	ab106410
Anti-UCP1 (for WB dil:1:1000 for WAT;1:2000 for BAT)(for IHC dil: 1:250 for WAT;1:500 for BAT)	Abcam	ab10983
Anti-PPARα, dil:1:1000	Abcam	ab215270
Anti-PPARβ, dil:1:1000	Abcam	ab23673
Anti-Sin3A, dil:1:1000	Abcam	ab3479
Anti-Ubiquitin, dil:1:1000	CST	20326S
Anti-PGC-1α, dil:1:1000	CST	2178S
Anti-PPARγ (for IP dil:1:200)	CST	2435S
Anti-Phospho-AMPKα (Thr172), dil:1:1000	CST	2535S
Anti-GST, dil:1:1000	CST	2624S
Anti-ACSS2, dil:1:1000	CST	3658S
Anti-NcoR1, dil:1:1000	CST	5948S
Anti-C/EBPα, dil:1:1000	CST	8178S
Anti-NF-κB p65 (D14E12), dil:1:1000	CST	8242S
Anti-Acetylated-Lysine, dil:1:1000	CST	9441S

Anti-Sirt1, dil:1:1000	CST	9475S
Anti-HA, dil:1:1000	Proteintech	51064-2-AP
Anti-Lamin B1, dil:1:1000	Proteintech	66095-1-Ig
Anti-P300, dil:1:1000	Santa Cruz	sc-32244
Anti-HDAC (A-3), dil:1:1000	Santa Cruz	sc-376957
Anti-ACSS2, dil:1:1000	Santa Cruz	sc-398559
Anti-ACLY, dil:1:1000	Santa Cruz	sc-517267
Anti-PPARγ (for WB dil:1:1000)	Santa Cruz	sc-7273
Anti-Flag, dil:1:1000	Sigma-Aldrich	F7425
Anti-GAPDH, dil:1:2000	ZSGB-BIO	TA-08
Anti-HSP90, dil:1:2000	ZSGB-BIO	TA-12
Goat anti-rabbit IgG-HRP, dil: 1:2000	Jackson ImmunoResearch Labs	111035144
Goat anti-mouse IgG-HRP, dil: 1:2000	Jackson ImmunoResearch Labs	115035146
Chemicals and inhibitors		
Anti-HA magnetic beads	Bimake	B26201
Anti-Flag magnetic beads	Bimake	B26101
Protease inhibitor cocktail	Bimake	B14002

Phosphatase inhibitor cocktail	Bimake	B15001
MitoTracker Deep Red FM	Invitrogen	M22426
DAPI	Invitrogen	Cat#S36964
D-Mannose	Macklin	D-813082
Insulin	Novo nordisk	N/A
FITC-D-Mannose	QiyueBio	QY-C-FDG5
GST-Sefinose(TM) Resin 4FF(Settled Resin)	Sangon Biotech	C600031
IPTG	Sangon Biotech	B300845-0005
Protein A/G-Sepharose	Santa Cruz	sc-2003
Glutathione-Agarose	Santa Cruz	sc-2009
Rosiglitazone	Selleck	S2556
GW9662	Selleck	S2915
ACSS2 inhibitor	Selleck	S8588
CL316243	MCE	HY-116771A
Type I collagenase	Worthington	LS004196
Indomethacin (IDM)	MCE	HY-14397
3,3',5-Triiodo-L-thyronine (T3)	Selleck	S5726
Oil Red O	Servicebio	G1015
Anti-His magnetic beads	Sigma-Aldrich	H9914
Streptavidin	Sigma-Aldrich	S1638

Ni-NTA His bind	Sigma-Aldrich	70666-4
Phenylmethylsulfonyl fluoride (PMSF)	Sigma-Aldrich	Cat#52332
D-Glucose	Sigma-Aldrich	Cat#608203
3-Isobutyl-1-methylxanthine (IBMX)	Sigma-Aldrich	Cat#I5879
Dexamethasone	Sigma-Aldrich	Cat#D4902
Critical Commercial Assays		
Mouse acetyl-CoA ELISA assay kit	Jianglai Bio	Cat#JL32779
Nonesterified Free fatty acids assay kit	Nanjing Jiancheng	A042-2-1
Triglyceride assay kit	Nanjing Jiancheng	A110-1-1
Total cholesterol assay kit	Nanjing Jiancheng	A111-1-1
BacTiter Glo kit	Promega	Cat#G8231
The Nuclear/Cytosol Fractionation Kit	Thermo	Cat#78833
The BCA Protein Assay Kit	Thermo	Cat#23225
ClonExpress II One Step Cloning Kit	Vazyme	Cat#C112-01
Dual luciferase assay system	Vazyme	Cat#DL101-01
Universal two-step assay kit	ZSGB-Bio	PV-9000
DAB Chromogenic Kit	ZSGB-BIO	ZLI-9018
Experimental Models: Cell Lines		
Cell line: HEK293T	Shanghai Cell Bank of Chinese	GNHu17

	Aca- demy of Sciences	
Experimental Models: Organism/strains		
Mouse: C57BL/6J	Charles River	N/A
Mouse: *Acss2*$^{-/-}$, *Acss2*$^{flox/flox}$, *Adipoq-cre*	GemPharmatech	N/A
Bacteria: *Escherichia coli* BL21(DE3)	Zhuangmeng Bio	ZK202
Bacteria: *Escherichia coli* DH5α	Zhuangmeng Bio	ZK206
Oligonucleotides		
Primers used for quantitative PCR, see Table S6	This study	N/A
Primers used for plasmids construction, see Table S7	This study	N/A
Recombinant DNA		
PPRE X3-TK-luc	addgene	1015
pBiFC-VN173	addgene	22010
pBiFC-VC155	addgene	22011
pBiFC-bJunVN173	addgene	22012
pBiFC-bFosVC155	addgene	22013
PGEX-4T	Miaoling Bio	P0001
PET28a	Miaoling Bio	P0023

pEGFP-C1	Miaoling Bio	P0134
pmCherry-N1	Miaoling Bio	P0475
pECMV-P300-myc	Miaoling Bio	P0691
pCMV-RXRA-Tag 2B	Miaoling Bio	P10927
pEnCMV-PPARD(human)-3×FLAG	Miaoling Bio	P18449
pEnCMV-PPARA(human)-3×FLAG	Miaoling Bio	P18450
pEnCMV-SIRT1(human)-3×HA	Miaoling Bio	P2135
pCMV-SPORT6-Creb1	Miaoling Bio	P3655
pCMV-PRDM16(mouse)-3×FLAG-SV40-Neo	Miaoling Bio	P38414
pECMV-CEBPβ-m-FLAG	Miaoling Bio	P5635
pGL3 basic	Promega	Cat#E1751
pBiFC-VC155-PPARγ	This study	N/A
pBiFC-VN173-ACSS2	This study	N/A
PGL3-UCP1 promoter-Luciferase (Human)	This study	N/A
PCMV-ACSS2	This study	N/A
PCMV-PPARγ	This study	N/A
PET28a-ACSS2	This study	N/A
PGEX-4T-PPARγ	This study	N/A
pmCherry-N1-ACSS2	This study	N/A

pEGFP-C1-PPARγ	This study	N/A
PGL3-PPARγ	This study	N/A
pCMV-MDM2(human)-3×FLAG-Neo	Miaoling Bio	P41596
pCMV-NEDD4(human)-3×HA-SV40-Neo	Miaoling Bio	P36058
pECMV-3×FLAG-TRIM25	Miaoling Bio	P3543
pCMV-TRIM27(human)-3×FLAG-Neo	Miaoling Bio	P50192
pCMV-STUB1(human)-3×FLAG-Neo	Miaoling Bio	P41623
pENTER-MKRN1	Weizhen Bio	CH899109
pcDNA3.1-3×FLAG-C-SIAH2	Fenghui Bio	NM-005067
Software		
CytExpert	Beckman Coulter	https://www.mybeckman.cn/flow-cytometry/research-flow-cytometers/cytoflex/software
FlowJo	Biosciences	https://www.bdbiosciences.com/zh-cn/products/softwa

		re/flowjo-v10-software
Graphpad Prism 9	Graphpad	https://www.graphpad.com/
OlyVIA	Olympus	https://olyvia.software.informer.com/
Living Image	PerkinElmer	https://www.perkinelmer.com.cn/
Zeiss Zen	Zeiss	https://www.zeiss.com
Image J		https://imagej.en.softonic.com
Other		
100µm cell strainer	BIOFIL	CSS013100
0.22µm filter	Millipore	SLGP033N
PVDF membrane	Millipore	IPVH00010
Mouse high fat diet	Trophic Animal Feed High-Tech Co	TP23300
Mouse normal chow	Beijing Keao Xieli	N/A

Table S1-Down genes in BAT of HFD VS NCD

gene id	gene name	description	gene_type	GO_id	GO_term	KO_ID	KO_Descript	kegg_id	kegg_term	locus	BAT_H1_cou	BAT_H2_cou	BAT_H3_cou	BAT_N1_cou	BAT_N2_cou	BAT_N3_cou	BAT_H1_FPK	BAT_H2_FPK	BAT_H3_FPK	BAT_N1_FPK	BAT_N2_FPK	BAT_N3_FPK	H	N	log2FC	Pvalue	Qvalue	updown
ENSMUSG00	Sorcs2	sortilin-relat	protein codi	GO:0023052	signaling|cell	-	-	-	-	5:36017180-	183	116	184	1398	1349	1268	1.0724255	0.6554143	1.0690231	8.0436543	7.9902854	6.5493773	0.9322877	7.5277723	-3.013376	1.30E-38	1.62E-34	DOWN
ENSMUSG00	Hcar1	hydrocarbox	protein codi	GO:0023052	signaling|res	K08401	hydroxycarb	mmu04024	cAMP signal	5:123876736	124	75	140	816	1148	880	2.2987889	1.3405532	2.573129	14.85252	21.510774	14.378955	2.0708237	16.914083	-3.029948	1.02E-30	8.43E-27	DOWN
ENSMUSG00	Acss2	acyl-CoA syn	protein codi	GO:0003824	catalytic acti	K01895	acetyl-CoA s	mmu01100|	Metabolic pa	2:155517948	3482	3088	3146	11552	11331	11198	16.565289	14.164173	14.838254	53.958338	54.484595	46.954422	15.189239	51.799118	-1.769878	7.79E-19	1.94E-15	DOWN
ENSMUSG00	Hcar2	hydroxycarb	protein codi	GO:0002376	immune syst	K08402	hydroxycarb	mmu04024	cAMP signal	5:123863570	21	21	34	185	271	178	0.6626536	0.6388834	1.0636396	5.7313848	8.6429314	4.9504156	0.7883922	6.4415773	-3.030429	7.22E-19	1.94E-15	DOWN
ENSMUSG00	Slc2a5	solute carrie	protein codi	GO:0050896	response to	K08143	MFS transpo	mmu04973	Carbohydrat	4:150119283	248	210	129	1023	878	1158	3.7852394	3.0903253	1.9520194	15.330206	13.544742	15.578139	2.942528	14.817696	-2.332193	2.47E-18	5.11E-15	DOWN
ENSMUSG00	Gm35853	predicted ge	antisense	-	-	-	-	-	-	15:10132288	59	55	58	308	253	260	0.8173481	0.7346255	0.7965994	4.1892753	3.5425246	3.1746443	0.7828577	3.6354814	-2.215325	4.08E-17	6.75E-14	DOWN
ENSMUSG00	Hsd11b1	hydroxysterc	protein codi	GO:0032502	developmen	K15680	corticosteroi	mmu01100|	Metabolic pa	1:193221634	913	726	1002	2548	2770	2770	8.8186019	6.7609764	9.5951236	24.163483	27.042331	23.581673	8.3915673	24.929162	-1.570822	5.48E-15	5.92E-12	DOWN
ENSMUSG00	Gpx8	glutathione p	protein codi	GO:0016209	antioxidant a	K00432	glutathione p	mmu01100|	Metabolic pa	13:11304275	319	416	426	1219	1574	1251	10.727201	13.48754	14.202299	40.246733	53.49775	37.078211	12.80568	43.607565	-1.767795	2.52E-14	2.61E-11	DOWN
ENSMUSG00	Acly	ATP citrate ly	protein codi	GO:0003824	catalytic acti	K01648	ATP citrate (	mmu01100|	Metabolic pa	11:10047635	22682	11809	12650	74365	56120	63672	168.65981	84.66171	93.255532	542.91264	421.77724	417.29629	115.52568	460.66206	-1.995495	3.34E-14	3.32E-11	DOWN
ENSMUSG00	Ptges	prostaglandi	protein codi	GO:0016209	antioxidant a	K15729	microsomal	mmu01100|	Metabolic pa	2:30889471-	47	49	97	400	318	280	0.5445715	0.5473878	1.1142585	4.5503994	3.7241033	2.8594517	0.7354059	3.7113182	-2.335319	7.02E-14	6.46E-11	DOWN
ENSMUSG00	Cyp2e1	cytochrome	protein codi	GO:0003824	catalytic acti	K07415	cytochrome	mmu01100|	Metabolic pa	7:140763739	267	231	548	1577	1996	1340	4.7641978	3.974053	9.6942007	27.627447	35.997603	21.074075	6.1441505	28.233042	-2.200099	3.69E-13	2.87E-10	DOWN
ENSMUSG00	Trf	transferrin [S	protein codi	GO:0032502	developmen	K14736	transferrin[-]	mmu04066|	HIF-1 signali	9:103204001	780	718	1165	3374	3487	2442	9.4436654	8.3813588	13.983824	40.107199	42.671049	26.058988	10.602949	36.279078	-1.774672	4.59E-13	3.46E-10	DOWN
ENSMUSG00	Mup22	major urinar	protein codi	GO:0005615	extracellular	-	-	-	-	4:60817589-	0	2	3	166	62	29	0	0.1363883	0.2103606	11.527873	4.4323992	1.8079005	0.115583	5.9227241	-5.67926	1.74E-12	1.20E-09	DOWN
ENSMUSG00	Pim1	proviral integ	protein codi	GO:0032502	developmen	K04702	proto-oncog	mmu05200|	Pathways in	17:29490753	1113	760	769	2460	2481	3050	8.8498777	5.8263836	6.062089	19.204738	19.939033	21.375078	6.9127834	20.17295	-1.545083	1.96E-12	1.32E-09	DOWN
ENSMUSG00	Acaca	acetyl-Coenz	protein codi	GO:0003824	catalytic acti	K11262	acetyl-CoA c	mmu01100|	Metabolic pa	11:84129672	15869	10922	9157	40876	38526	37053	47.674764	31.636235	27.273822	120.56979	116.98445	98.113359	35.528274	111.8892	-1.655031	4.85E-12	3.17E-09	DOWN
ENSMUSG00	Gsta3	glutathione S	protein codi	GO:0032502	developmen	K00799	glutathione S	mmu01100|	Metabolic pa	1:21240589-	45	42	55	260	203	155	0.5768281	0.5190676	0.698954	3.2721506	2.6300264	1.7511592	0.5982832	2.5511121	-2.092226	2.18E-11	1.20E-08	DOWN
ENSMUSG00	Isyna1	myo-inosito	protein codi	GO:0003824	catalytic acti	K01858	myo-inosito	mmu01100|	Metabolic pa	8:70594373-	131	111	97	412	401	303	1.6311242	1.3325529	1.1974121	5.0366798	5.0465662	3.3252552	1.3870298	4.4695004	-1.688115	7.54E-11	3.60E-08	DOWN
ENSMUSG00	B3galt2	UDP-Gal:bet	protein codi	GO:0003824	catalytic acti	K07820	beta-1,3-ga	mmu01100|	Metabolic pa	1:143640664	292	294	328	688	1092	894	1.9145826	1.8585894	2.1321609	4.4290609	7.2368544	5.1664837	1.9684443	5.6107997	-1.511151	9.36E-11	4.38E-08	DOWN
ENSMUSG00	Lctl	lactase-like [	protein codi	GO:0032502	developmen	-	-	-	-	9:64117147-	257	206	355	613	1043	1198	4.0822039	3.1548089	5.5904167	9.5599121	16.744854	16.77199	4.2758098	14.358919	-1.747678	1.35E-10	6.08E-08	DOWN
ENSMUSG00	Nmt2	N-myristoylt	protein codi	GO:0003824	catalytic acti	-	-	-	-	2:3284212-3	231	200	335	693	722	753	1.7642117	1.472692	2.5365093	5.1963941	5.5732751	5.0687269	1.924471	5.2794654	-1.45593	1.55E-10	6.86E-08	DOWN
ENSMUSG00	Slc25a10	solute carrie	protein codi	GO:0009987	cellular proc	K13577	solute carrie	mmu04964	Proximal tub	11:12049184	251	187	457	1274	1136	902	2.9779413	2.1390848	5.3754272	14.84033	13.622485	9.4322478	3.4974845	12.631687	-1.852658	1.61E-10	7.03E-08	DOWN
ENSMUSG00	Scd1	stearoyl-Coe	protein codi	GO:0032502	developmen	K00507	stearoyl-CoA	mmu01100|	Metabolic pa	19:44394455	269416	174324	217560	619285	603640	537598	2998.4131	1870.5482	2400.4957	6766.8998	6790.1802	5273.4095	2423.1523	6276.8298	-1.373151	1.96E-10	8.25E-08	DOWN
ENSMUSG00	Chn1	chimerin 1 [S	protein codi	GO:0032502	developmen	-	-	-	-	2:73610660-	205	253	253	649	590	734	1.0157363	1.2086223	1.2427994	3.1572051	2.9547064	3.2054488	1.1557193	3.1057867	-1.426168	2.07E-10	8.57E-08	DOWN
ENSMUSG00	Slc25a1	solute carrie	protein codi	GO:0009987	cellular proc	-	-	-	-	16:17925223	6586	4916	3362	16110	16332	15051	106.24755	76.463248	53.771071	255.16662	266.30039	214.00718	78.82729	245.15806	-1.636945	2.22E-10	8.89E-08	DOWN
ENSMUSG00	Thrsp	thyroid horm	protein codi	GO:0008152	metabolic pr	-	-	-	-	7:97412938-	26803	22711	22142	55900	62575	59840	609.74633	498.13344	499.38609	1248.558	1438.8047	1199.8395	535.75529	1295.7341	-1.274124	3.68E-10	1.41E-07	DOWN
ENSMUSG00	Pth1r	parathyroid	protein codi	GO:0032502	developmen	K04585	parathyroid	mmu04080|	Neuroactive	9:110722085	277	259	225	832	890	568	3.59148	3.2376981	2.8922135	10.59127	11.663232	6.4909373	3.2404639	9.5818131	-1.564098	4.51E-10	1.70E-07	DOWN
ENSMUSG00	Mycl	v-myc avian	protein codi	GO:0032502	developmen	-	-	-	-	4:122995652	13	3	22	113	154	69	0.1320167	0.0293767	0.2214907	1.1266471	1.580655	0.6175813	0.127628	1.1082945	-3.118324	6.86E-10	2.50E-07	DOWN
ENSMUSG00	Igkv1-110	immunoglob	IG_V_gene	GO:0002376	immune syst	-	-	-	-	6:68270485-	5	2	6	31	33	52	0.7593198	0.2928438	0.9033441	4.622384	5.0654614	6.9604671	0.6518359	5.5494375	-3.089761	8.88E-10	3.15E-07	DOWN
ENSMUSG00	Mt2	metallothion	protein codi	GO:0023052	signaling|res	K14739	metallothion	mmu04978	Mineral abso	8:94172664-	154	179	153	348	629	635	11.258712	12.617288	11.089537	24.979263	46.478814	40.917466	11.655179	37.458514	-1.684323	1.70E-09	5.63E-07	DOWN
ENSMUSG00	Chst12	carbohydrate	protein codi	GO:0003824	catalytic acti	K04742	chondroitin 4	mmu00532	Glycosaminc	5:140505550	250	207	150	575	543	610	4.9160035	3.924516	2.9242698	11.101244	10.792143	10.572275	3.9215964	10.821887	-1.464439	3.06E-09	9.85E-07	DOWN
ENSMUSG00	Car5b	carbonic anh	protein codi	GO:0003824	catalytic acti	K01672	carbonic anh	mmu01100|	Metabolic pa	X:163976822	363	430	570	1162	1132	1196	3.3530354	3.8295082	5.2198675	10.538248	10.568483	9.7370682	4.134137	10.281267	-1.31436	3.21E-09	1.02E-06	DOWN
ENSMUSG00	Smoc1	SPARC relate	protein codi	GO:0032502	developmen	-	-	-	-	12:81026808	698	528	610	1600	1638	1293	7.3978238	5.3954299	6.4096157	16.649436	17.546784	12.078497	6.4009565	15.424906	-1.268902	3.68E-09	1.13E-06	DOWN
ENSMUSG00	Atp1a3	ATPase, Na+	protein codi	GO:0032502	developmen	K01539	sodium/pota	mmu04024|	cAMP signal	7:24978167-	81	33	28	251	241	184	0.6182346	0.2428409	0.2118712	1.8809239	1.8591629	1.2377942	0.3576489	1.6592937	-2.213953	3.93E-09	1.18E-06	DOWN
ENSMUSG00	Gm7049	predicted ge	processed p	-	-	-	-	-	-	13:62813076	849	569	443	2060	1735	1624	30.20078	19.514888	15.623117	71.946335	62.379948	50.91693	21.779595	61.747738	-1.503409	5.11E-09	1.49E-06	DOWN
ENSMUSG00	Irf4	interferon re	protein codi	GO:0032502	developmen	K09445	interferon re	mmu04659	Th17 cell diff	13:30749226	61	60	92	160	309	457	0.7054466	0.6690018	1.0548068	1.816701	3.6118236	4.6581648	0.8097517	3.3622298	-2.053867	6.58E-09	1.84E-06	DOWN
ENSMUSG00	Vat1l	vesicle amine	protein codi	GO:0003824	catalytic acti	-	-	-	-	8:114205612	77	79	146	237	412	545	0.5309992	0.5252608	0.9981848	1.6046593	2.8716813	3.3125793	0.6848149	2.5963066	-1.922675	6.48E-09	1.84E-06	DOWN
ENSMUSG00	Sytl1	synaptotagm	protein codi	GO:0009987	cellular proc	-	-	-	-	4:133253090	17	27	39	100	108	165	0.3006917	0.460452	0.683907	1.7366367	1.9307899	2.5723362	0.4816836	2.0799209	-2.110371	1.01E-08	2.76E-06	DOWN
ENSMUSG00	Mapk8ip1	mitogen-act	protein codi	GO:0002376	immune syst	K04434	mitogen-act	mmu04010	MAPK signal	2:92383676-	130	55	76	361	271	275	1.3049149	0.5322917	0.7563271	3.5577798	2.7494409	2.4329826	0.8645112	2.9134011	-1.752748	1.08E-08	2.92E-06	DOWN
ENSMUSG00	Cyp2f2	cytochrome	protein codi	GO:0003824	catalytic acti	K07416	cytochrome	mmu00980	Metabolism	7:27119909-	15	9	18	175	64	63	0.4054702	0.2345597	0.4823727	4.6443208	1.7485121	1.5009221	0.3741342	2.6312517	-2.814122	1.85E-08	4.75E-06	DOWN
ENSMUSG00	Me1	malic enzym	protein codi	GO:0003824	catalytic acti	K00029	malate dehy	mmu01100|	Metabolic pa	9:86581371-	7028	4978	3395	16219	14941	13538	76.60679	52.31594	36.688428	173.57639	164.60779	130.0636	55.203719	156.08259	-1.499472	2.19E-08	5.55E-06	DOWN
ENSMUSG00	Gm28875	predicted ge	lincRNA	-	-	-	-	-	-	12:10492550	14	12	13	40	76	96	0.7170852	0.5925838	0.6601499	2.0114984	3.9344078	4.3338258	0.6566063	3.4265773	-2.383668	2.80E-08	6.96E-06	DOWN
ENSMUSG00	Net1	neuroepithel	protein codi	GO:0003824	catalytic acti	-	-	-	-	13:3882018-	292	226	298	796	1268	542	2.2766154	1.6988658	2.3034413	6.0932905	9.9922181	3.7245403	2.0929742	6.6033496	-1.657644	2.94E-08	7.17E-06	DOWN
ENSMUSG00	Gm45774	predicted ge	antisense	-	-	-	-	-	-	8:94170748-	22	18	17	64	73	95	0.5027275	0.3965753	0.3851453	1.4359233	1.6860629	1.9134087	0.4281494	1.6784649	-1.970956	3.06E-08	7.37E-06	DOWN
ENSMUSG00	Fasn	fatty acid syn	protein codi	GO:0032502	developmen	K00665	fatty acid syn	mmu01100|	Metabolic pa	11:12080584	166611	102372	91885	426941	328337	279039	728.81316	431.75499	398.48407	1833.6292	1451.6689	1075.8289	519.68407	1453.709	-1.484032	3.29E-08	7.77E-06	DOWN
ENSMUSG00	Tmem120b	transmembra	protein codi	GO:0032502	developmen	-	-	-	-	5:123068415	920	717	746	1670	1850	1822	12.284059	9.2303208	9.8752218	21.892818	24.966696	21.442146	10.4632	22.76722	-1.121634	3.33E-08	7.79E-06	DOWN
ENSMUSG00	Retn	resistin [Sour	protein codi	GO:0032502	developmen	-	-	-	-	8:3655770-3	354	298	753	1435	1825	1319	12.526691	10.167019	26.416909	49.855887	65.272668	41.138054	16.370206	52.08887	-1.669903	3.69E-08	8.55E-06	DOWN
ENSMUSG00	Ptp4a1	protein tyros	protein codi	GO:0032502	developmen	-	-	-	-	1:30940302-	1523	1805	2558	4245	5140	5198	12.239383	13.985578	20.380459	33.494103	41.750136	36.818121	15.53514	37.35412	-1.265732	5.45E-08	1.19E-05	DOWN
ENSMUSG00	Gm20458	predicted ge	protein codi	GO:0009987	cellular proc	-	-	-	-	2:164460967	0	0	0	57	6	15	0	0	0	2.4895271	0.2697637	0.5881066	0	1.1157991	#NAME?	5.47E-08	1.19E-05	DOWN
ENSMUSG00	Gm5182	predicted ge	processed p	-	-	-	-	-	-	12:10152142	5507	2565	3983	15957	10056	9697	55.051529	24.722092	39.474658	156.61618	101.60492	85.439253	39.749426	114.55345	-1.527015	7.47E-08	1.56E-05	DOWN
ENSMUSG00	Gm6781	predicted ge	processed p	-	-	-	-	-	-	14:18531363	23	25	40	86	106	133	1.1277608	1.1818887	1.944497	4.1402214	5.2533073	5.7479042	1.4180488	5.0471443	-1.83156	8.29E-08	1.71E-05	DOWN
ENSMUSG00	Fdft1	farnesyl diph	protein codi	GO:0003824	catalytic acti	K00801	farnesyl-dipl	mmu01100|	Metabolic pa	14:63145150	408	602	680	1439	1594	1258	3.8214411	5.4363451	6.3143721	13.233028	15.090051	10.385171	5.1907195	12.90275	-1.313672	1.06E-07	2.14E-05	DOWN
ENSMUSG00	Rgma	repulsive gui	protein codi	GO:0032502	developmen	K23096	repulsive gui	mmu04360|	Axon guidan	7:73375509-	265	205	253	515	496	658	2.4943411	1.8604012	2.3609347	4.759358	4.7187483	5.4588553	2.238559	4.9789872	-1.153282	1.79E-07	3.53E-05	DOWN
ENSMUSG00	Ccdc57	coiled-coil d	protein codi	GO:0009987	cellular proc	-	-	-	-	11:12082652	53	25	21	165	90	174	0.7537751	0.3428084	0.2961014	2.3040021	1.293739	2.1811336	0.4642283	1.9262916	-2.05292	2.25E-07	4.30E-05	DOWN
ENSMUSG00	Cfd	complement	protein codi	GO:0002376	immune syst	K01334	complement	mmu05171|	Coronavirus	10:79890853	4738	3583	10926	17856	21409	24920	229.18356	167.10094	523.9664	848.01538	1046.6947	1062.4347	306.7503	985.71493	-1.684106	2.41E-07	4.58E-05	DOWN
ENSMUSG00	Gucd1	guanylyl cycl	protein codi	-	-	-	-	-	-	10:75351111	666	617	822	1403	1445	1569	3.0612944	2.7343852	3.7459024	6.3316917	6.7132722	6.3565237	3.1805273	6.4671625	-1.023867	4.17E-07	7.39E-05	DOWN
ENSMUSG00	Elovl6	ELOVL family	protein codi	GO:0003824	catalytic acti	K10203	elongation c	mmu01100|	Metabolic pa	3:129532355	5690	5089	3523	12311	10682	10766	31.350628	27.033988	19.244226	66.597579	59.486959	52.282234	25.876281	59.455591	-1.200182	5.87E-07	0.0001012	DOWN
ENSMUSG00	Pcmtd1	protein-L-iso	protein codi	GO:0003824	catalytic acti	-	-	-	-	1:7088920-7	363	284	440	524	1494	1614	1.2639544	0.9534233	1.5189054	1.7913746	5.2578736	4.9532898	1.2454277	4.000846	-1.683664	6.65E-07	0.0001131	DOWN
ENSMUSG00	Tkt	transketolase	protein codi	GO:0003824	catalytic acti	K00615	transketolase	mmu01100|	Metabolic pa	14:30548359	32040	24117	24672	54644	53884	60407	203.18879	147.45997	155.11914	340.23644	345.38405	337.64496	168.5893	341.08848	-1.016633	7.41E-07	0.0001245	DOWN
ENSMUSG00	Irs1	insulin recep	protein codi	GO:0060090	molecular ad	K16172	insulin recep	mmu05010|	Alzheimer di	1:82233101-	339	265	259	558	710	649	2.2577617	1.7016417	1.7101365	3.648745	4.7793765	3.8096765	1.8898467	4.079266	-1.11004	7.46E-07	0.0001245	DOWN
ENSMUSG00	Clmn	calmin [Sour	protein codi	GO:0032502	developmen	-	-	-	-	12:10476311	114	56	123	272	279	250	0.5098441	0.2414704	0.545371	1.1943518	1.2611637	0.9854587	0.4322285	1.1469914	-1.407989	7.79E-07	0.000128	DOWN
ENSMUSG00	Gm7329	predicted ge	processed p	-	-	-	-	-	-	1:65095227-	123	99	48	313	266	267	35.669876	27.680388	13.800254	89.118966	77.967153	68.245137	25.716839	78.443752	-1.608945	1.07E-06	0.0001702	DOWN
ENSMUSG00	Pmvk	phosphomev	protein codi	GO:0003824	catalytic acti	K13273	phosphomev	mmu01100|	Metabolic pa	3:89454541-	602	507	418	1162	1271	980	11.701737	9.5017859	8.0553387	22.176403	24.970912	16.78981	9.7529537	21.312375	-1.12778	1.18E-06	0.0001836	DOWN
ENSMUSG00	Fam117a	family with s	protein codi	-	-	-	-	-	-	11:95337018	276	278	276	615	746	512	4.3897288	4.2630145	4.3520242	9.6036267	11.992301	7.1773563	4.3349225	9.5910948	-1.145689	1.19E-06	0.0001839	DOWN
ENSMUSG00	Orm1	orosomucoid	protein codi	GO:0002376	immune syst	-	-	-	-	4:63344560-	604	792	729	1412	1703	1496	47.462401	60.004143	56.79287	108.93773	135.25777	103.61195	54.753138	115.93582	-1.082313	1.39E-06	0.0002113	DOWN
ENSMUSG00	Igf1	insulin-like g	protein codi	GO:0032502	developmen	K05459	insulin-like g	mmu05200|	Pathways in	10:87858265	162	135	199	381	414	334	0.7505877	0.6030646	0.9141017	1.7331775	1.9387492	1.3639528	0.755918	1.6786265	-1.15098	1.70E-06	0.0002524	DOWN
ENSMUSG00	Ppp1r10	protein phos	protein codi	GO:0003824	catalytic acti	-	-	-	-	17:35916434	451	387	312	1026	632	1152	3.0409323	2.5158513	2.0856368	6.7921751	4.3070755	6.8461823	2.5474735	5.981811	-1.231515	1.81E-06	0.000264	DOWN
ENSMUSG00	Apoe	apolipoprote	protein codi	GO:0032502	developmen	K04524	apolipoprote	mmu05010|	Alzheimer di	7:19696109-	2374	2164	4031	5188	7709	7943	22.204813	19.514944	37.379461	47.642825	72.878555	65.481224	26.366406	62.000868	-1.233587	1.95E-06	0.0002836	DOWN
ENSMUSG00	Rnf144b	ring finger p	protein codi	GO:0003824	catalytic acti	-	-	-	-	13:47122656	182	111	157	294	344	395	1.5849758	0.9320058	1.3555185	2.5137864	3.0279158	3.0318895	1.2908334	2.8578639	-1.146634	2.89E-06	0.0004102	DOWN
ENSMUSG00	Gm10642	predicted ge	antisense	-	-	-	-	-	-	9:70656251-	33	18	33	94	116	68	0.623748	0.3280178	0.6183895	1.7444101	2.2160517	1.1328212	0.5233851	1.697761	-1.697689	3.53E-06	0.0004875	DOWN
ENSMUSG00	Spire1	spire type ac	protein codi	GO:0032502	developmen	-	-	-	-	18:67488209	417	293	308	759	665	681	1.5610485	1.0575262	1.1431017	2.7896709	2.5161517	2.2469473	1.2538921	2.5175899	-1.00563	4.68E-06	0.0006308	DOWN
ENSMUSG00	Fcor	Foxo1 corep	protein codi	GO:0032502	developmen	-	-	-	-	8:3671188-3	147	112	130	244	321	294	22.837405	16.776034	20.022783	37.21765	50.40444	40.256917	19.878741	42.626336	-1.100519	5.50E-06	0.0007183	DOWN
ENSMUSG00	Tprkb	Tp53rk bindi	protein codi	GO:0008152	metabolic pr	-	-	-	-	6:85911865-	81	47	99	214	179	193	0.5010557	0.2803088	0.6071396	1.2997019	1.1191432	1.0522571	0.4628347	1.1570341	-1.321862	5.83E-06	0.0007543	DOWN
ENSMUSG00	Nr4a1	nuclear rece	protein codi	GO:0032502	developmen	K04465	nuclear rece	mmu04151|	PI3K-Akt sig	15:10125426	221	211	112	285	670	3830	3.551131	3.2689022	1.7842072	4.4962567	10.881401	54.242476	2.8680801	23.206711	-3.016385	6.12E-06	0.0007826	DOWN
ENSMUSG00	Atp4b	ATPase, H+/	protein codi	GO:0003824	catalytic acti	K01543	H+/K+-exch	mmu01100|	Metabolic pa	8:13386205-	37	29	18	84	72	131	1.2219594	0.9234092	0.5893632	2.7237372	2.4033658	3.8131928	0.9115773	2.9800986	-1.708923	7.70E-06	0.0009458	DOWN
ENSMUSG00	Gm35986	predicted ge	antisense	-	-	-	-	-	-	3:129527467	20	13	9	57	51	43	1.255682	0.7869316	0.5601664	3.5135696	3.2363234	2.3794709	0.8675933	3.0431213	-1.810461	8.28E-06	0.0009943	DOWN
ENSMUSG00	Elovl5	ELOVL family	protein codi	GO:0003824	catalytic acti	K10244	elongation c	mmu01100|	Metabolic pa	9:77917364-	1229	934	1067	2822	2498	1665	19.285128	14.130617	16.599258	43.476912	39.618507	23.027699	16.671667	35.374373	-1.085306	8.65E-06	0.0010222	DOWN
ENSMUSG00	Mr1	major histoc	protein codi	GO:0032502	developmen	-	-	-	-	1:155127277	100	83	110	205	191	291	0.5654009	0.4524593	0.6166	1.138001	1.0915089	1.4501633	0.54482	1.2265577	-1.170763	9.14E-06	0.0010655	DOWN
ENSMUSG00	Tst	thiosulfate su	protein codi	GO:0032502	developmen	K01011	thiosulfate/3	mmu01100|	Metabolic pa	15:78399556	324	421	427	774	925	740	5.2241062	6.5447544	6.8257182	12.252902	15.074544	10.516343	6.1981929	12.614596	-1.025174	9.50E-06	0.0010924	DOWN
ENSMUSG00	Pqlc3	PQ loop rep	protein codi	GO:0031224	intrinsic com	-	-	-	-	12:16988648	116	113	145	239	258	319	0.8098552	0.7606267	1.0036222	1.6382426	1.8205541	1.9629286	0.8580347	1.8072418	-1.074682	1.04E-05	0.001183	DOWN
ENSMUSG00	Nr4a2	nuclear rece	protein codi	GO:0032502	developmen	K08558	nuclear rece	mmu04928|	Parathyroid	2:57106830-	104	85	98	161	232	510	0.9953752	0.7843579	0.9298941	1.512898	2.2442717	4.3021732	0.9032091	2.6864476	-1.572568	1.17E-05	0.0013188	DOWN
ENSMUSG00	Hvcn1	hydrogen vo	protein codi	GO:0008152	metabolic pr	-	-	-	-	5:122206804	40	26	50	138	85	111	0.5777895	0.3620955	0.7160342	1.9571461	1.240995	1.4131966	0.5519731	1.5371126	-1.477553	1.25E-05	0.0013905	DOWN
ENSMUSG00	Sik1	salt inducible	protein codi	GO:0032502	developmen	K19008	serine/threo	mmu04922	Glucagon sig	17:31844250	443	308	319	563	780	1444	4.4285101	2.9685802	3.1615375	5.5257801	7.881055	12.722934	3.5195426	8.709923	-1.307272	1.32E-05	0.0014583	DOWN
ENSMUSG00	Unc119	unc-119 lipid	protein codi	GO:0032502	developmen	-	-	-	-	11:78343482	115	110	123	216	316	242	1.0861441	1.001671	1.151724	2.0029697	3.0165609	2.0145106	1.0798464	2.3446804	-1.118565	1.69E-05	0.0018138	DOWN
ENSMUSG00	Sctr	secretin rece	protein codi	GO:0032502	developmen	K04588	secretin rece	mmu04080|	Neuroactive	1:120006894	127	110	136	213	266	351	2.8361758	2.3684543	3.0110871	4.6702394	6.0040712	6.9087945	2.7385724	5.8610351	-1.097731	1.76E-05	0.001884	DOWN
ENSMUSG00	Igkc	immunoglob	IG_C_gene	-	-	-	-	-	-	6:70726435-	181	185	332	396	707	594	20.719598	20.418218	37.67858	44.507015	81.800687	59.931451	26.272132	62.079718	-1.240589	1.89E-05	0.0020056	DOWN
ENSMUSG00	Aacs	acetoacetyl-	protein codi	GO:0032502	developmen	K01907	acetoacetyl-	mmu01100|	Metabolic pa	5:125475814	3705	2189	2252	7952	5355	5033	47.915241	27.294481	28.874047	100.97014	69.997125	57.369116	34.69459	76.112128	-1.133416	1.94E-05	0.0020449	DOWN
ENSMUSG00	Tmie	transmembra	protein codi	GO:0032502	developmen	-	-	-	-	9:110865711	34	16	15	101	54	72	0.6082736	0.275982	0.2660591	1.7740897	0.976465	1.1353376	0.3834382	1.2952974	-1.756217	2.20E-05	0.0022353	DOWN
ENSMUSG00	Acsl5	acyl-CoA syn	protein codi	GO:0009758	carbohydrate	K01897	long-chain a	mmu01100|	Metabolic pa	19:55251938	4626	3373	2232	8534	7602	6862	50.11944	35.233889	23.974445	90.778782	83.246051	65.526514	36.442591	79.850449	-1.131675	2.77E-05	0.0027471	DOWN
ENSMUSG00	2010003K11	RIKEN cDNA	protein codi	-	-	-	-	-	-	19:4496788-	160	154	118	300	388	270	8.3495327	7.748325	6.1049263	15.370797	20.464946	12.418623	7.400928	16.084789	-1.119919	3.19E-05	0.0031102	DOWN
ENSMUSG00	Gprc5c	G protein-co	protein codi	GO:0023052	signaling|res	-	-	-	-	11:11485115	123	101	126	215	237	274	0.8593179	0.6803157	0.8727127	1.4747421	1.673518	1.6871884	0.8041154	1.6118161	-1.003213	3.49E-05	0.0033409	DOWN
ENSMUSG00	AA914427	EST AA91442	TEC	-	-	-	-	-	-	16:17922043	80	29	13	168	190	140	4.1783736	1.4603486	0.6731717	8.614993	10.030096	6.444802	2.1039646	8.3632968	-1.990961	3.58E-05	0.0034145	DOWN
ENSMUSG00	Gm20716	predicted ge	protein codi	GO:0003824	catalytic acti	-	-	-	-	2:168180986	0	0	0	0	0	1092	0	0	0	0	0	11.084369	0	3.6947898	#NAME?	4.86E-05	0.0043601	DOWN
ENSMUSG00	Zfp703	zinc finger p	protein codi	GO:0032502	developmen	-	-	-	-	8:26977325-	807	576	814	1274	1163	2243	11.321344	7.7909517	11.321463	17.547895	16.490707	27.734446	10.144586	20.591016	-1.021305	5.21E-05	0.0046337	DOWN
ENSMUSG00	Serpina3n	serine (or cys	protein codi	GO:0005615	extracellular	-	-	-	-	12:10440672	62	57	134	329	199	180	1.8293566	1.621533	3.9197795	9.5308389	5.9346178	4.6810425	2.4568897	6.7154997	-1.450662	5.49E-05	0.0048309	DOWN
ENSMUSG00	Scd2	stearoyl-Coe	protein codi	GO:0003824	catalytic acti	K00507	stearoyl-CoA	mmu01100|	Metabolic pa	19:44293674	2978	1571	2226	8839	5732	3110	25.16427	12.799098	18.648275	73.332063	48.95545	23.162522	18.870548	48.483345	-1.361353	6.33E-05	0.0054569	DOWN
ENSMUSG00	Oscp1	organic solut	protein codi	GO:0050896	response to	-	-	-	-	4:126058565	66	62	102	134	181	252	0.6274382	0.5682788	0.9613503	1.2507276	1.7391613	2.1115143	0.7190224	1.7004677	-1.241823	6.63E-05	0.0056731	DOWN
ENSMUSG00	Esr1	estrogen rec	protein codi	GO:0032502	developmen	K08550	estrogen rec	mmu05200|	Pathways in	10:4611593-	110	105	266	433	362	388	0.3525031	0.3244172	0.845094	1.3623479	1.1725005	1.0958883	0.5073381	1.2102455	-1.25428	8.18E-05	0.0067946	DOWN
ENSMUSG00	Gm49513	predicted ge	processed tr	-	-	-	-	-	-	15:84181914	2	4	5	9	23	38	0.402046	0.7751179	0.9962623	1.7760943	4.6724064	6.7315608	0.7244754	4.3933539	-2.600314	8.27E-05	0.0068411	DOWN
ENSMUSG00	Mmd2	monocyte to	protein codi	GO:0032502	developmen	-	-	-	-	5:142562358	47	77	104	127	234	259	0.5285813	0.8349303	1.1595858	1.4023359	2.6599083	2.567332	0.8410325	2.2098587	-1.393721	9.49E-05	0.007721	DOWN
ENSMUSG00	mt-Ts2	mitochondri	Mt_tRNA	-	-	-	-	-	-	MT:11613-1	1	1	1	339	1	1	1.0323723	0.9956186	1.0232796	343.55203	1.0432387	0.9099113	1.0170902	115.16839	-6.823153	0.000109	0.0087148	DOWN
ENSMUSG00	Gm35290	predicted ge	antisense	-	-	-	-	-	-	17:28001065	31	33	39	78	80	89	1.4544653	1.492779	1.8140923	3.5930821	3.7936943	3.6804134	1.5871122	3.6890633	-1.21685	0.0001148	0.0090503	DOWN
ENSMUSG00	Kit	KIT proto-on	protein codi	GO:0032502	developmen	K05091	proto-oncog	mmu05200|	Pathways in	5:75574916-	168	117	157	751	191	350	0.7895603	0.5301595	0.7315281	3.465352	0.9072884	1.4498069	0.6837493	1.9408158	-1.505124	0.0001368	0.010353	DOWN
ENSMUSG00	Tmem179	transmembra	protein codi	GO:0031224	intrinsic com	-	-	-	-	12:11250018	234	138	323	577	464	442	5.3094229	3.0189465	7.2658645	12.854001	10.641059	8.839336	5.198078	10.778132	-1.052057	0.0001466	0.011031	DOWN
ENSMUSG00	Chst1	carbohydrate	protein codi	GO:0003824	catalytic acti	K01022	keratan sulfa	mmu00533	Glycosaminc	2:92599707-	123	76	110	272	225	173	2.7950276	1.6650769	2.4781337	6.0684661	5.1677028	3.464899	2.3127461	4.900356	-1.08328	0.0001491	0.01108	DOWN
ENSMUSG00	Anxa8	annexin A8 [	protein codi	GO:0003824	catalytic acti	-	-	-	-	14:34085981	7	10	16	22	63	43	0.1870589	0.2576357	0.4238871	0.5771915	1.7015563	1.0127629	0.2895272	1.0971702	-1.922016	0.0001709	0.0123666	DOWN
ENSMUSG00	Gm49492	predicted ge	bidirectional	-	-	-	-	-	-	15:10060020	28	37	49	79	81	137	0.6653009	0.8476348	1.1542828	1.8429894	1.9452853	2.8691318	0.8890728	2.2191355	-1.319624	0.000202	0.0140488	DOWN
ENSMUSG00	Cldn24	claudin 24 [S	protein codi	GO:0005198	structural mo	K06087	claudin[-]	mmu04514|	Cell adhesio	8:47822143-	2	2	5	10	17	20	0.1837405	0.1771197	0.4553054	0.901837	1.5783298	1.6192184	0.2720552	1.3664617	-2.328474	0.0002036	0.0141172	DOWN
ENSMUSG00	Eci3	enoyl-Coenz	protein codi	GO:0003824	catalytic acti	K13239	Delta3-Delta	mmu04146|	Peroxisome	13:34946614	144	132	175	237	262	542	5.5328284	4.8899441	6.6661601	8.9405337	10.174699	18.354817	5.6963108	12.490016	-1.132676	0.0002076	0.0142772	DOWN
ENSMUSG00	Orm3	orosomucoid	protein codi	GO:0002376	immune syst	-	-	-	-	4:63356162-	48	49	38	116	119	84	3.7816011	3.7219535	2.9680703	8.972715	9.475855	5.8328641	3.4905416	8.0938114	-1.213368	0.0002137	0.0145369	DOWN
ENSMUSG00	Gm37305	predicted ge	antisense	-	-	-	-	-	-	3:65957758-	53	32	49	72	136	123	0.7543035	0.4391069	0.6913827	1.0060925	1.956346	1.5429193	0.6282644	1.5017859	-1.257235	0.0002448	0.0162828	DOWN
ENSMUSG00	Tspan4	tetraspanin 4	protein codi	GO:0009987	cellular proc	-	-	-	-	7:141475240	365	451	399	547	789	1245	5.8449434	6.963185	6.3345298	8.6001535	12.770273	17.57207	6.3808861	12.980832	-1.024554	0.0002569	0.0169204	DOWN
ENSMUSG00	Fer1l6	fer-1-like 6 (	protein codi	GO:0050896	response to	-	-	-	-	15:58510048	87	66	90	136	167	207	0.6152933	0.4500353	0.6310377	0.9443438	1.1937466	1.2903152	0.5654555	1.1428019	-1.01509	0.0002591	0.0169694	DOWN
ENSMUSG00	Fbxo36	F-box protei	protein codi	GO:0005515	protein bind	-	-	-	-	1:84839841-	38	28	46	61	106	112	1.0221745	0.7261827	1.2267254	1.6110114	2.8818938	2.6553541	0.9916942	2.3827531	-1.264662	0.0002896	0.0185275	DOWN
ENSMUSG00	Fxyd7	FXYD domai	protein codi	GO:0098772	molecular fu	-	-	-	-	7:31042513-	7	3	5	20	15	23	0.5500738	0.2273185	0.3895064	1.543014	1.1913113	1.5929612	0.3889662	1.4424288	-1.890783	0.0002929	0.0186937	DOWN
ENSMUSG00	Gm454	predicted ge	transcribed	-	-	-	-	-	-	5:138202134	7	7	9	26	32	20	0.3251771	0.313526	0.4144634	1.1858021	1.5024401	0.8188725	0.3510555	1.1690382	-1.735551	0.0003156	0.0197836	DOWN
ENSMUSG00	Ttc25	tetratricopep	protein codi	GO:0032502	developmen	-	-	-	-	11:10054560	314	140	93	746	383	365	2.5095111	1.0787773	0.7368748	5.85368	3.0938105	2.5710913	1.4417211	3.8395273	-1.413137	0.0004891	0.028105	DOWN
ENSMUSG00	Sp3os	trans-acting	antisense	-	-	-	-	-	-	2:72979424-	12	15	16	25	66	43	0.4675588	0.5635022	0.618067	0.9563821	2.5991683	1.476703	0.5497093	1.6774178	-1.609501	0.0005227	0.0296932	DOWN
ENSMUSG00	Sco2	SCO2 cytoch	protein codi	GO:0032502	developmen	K23755	protein SCO	mmu05230	Central carb	15:89371637	62	50	34	160	156	70	1.5765311	1.2258086	0.8571292	3.9944666	4.009307	1.5688182	1.2198229	3.1908639	-1.387275	0.0005434	0.0306783	DOWN
ENSMUSG00	Gm5873	predicted ge	processed p	-	-	-	-	-	-	6:6735427-6	24	15	19	53	44	45	1.3710965	0.8262233	1.0761343	2.9727912	2.5406303	2.2658585	1.0911513	2.5930933	-1.248823	0.0005774	0.0321386	DOWN
ENSMUSG00	Pfn2	profilin 2 [Sc	protein codi	GO:0003824	catalytic acti	K05759	profilin[-]	mmu05014|	Amyotrophic	3:57841895-	70	74	58	112	133	186	0.7680985	0.7828785	0.6309641	1.2066201	1.4750543	1.7988701	0.7273137	1.4935148	-1.038062	0.0007334	0.0390713	DOWN
ENSMUSG00	Pstpip2	proline-serin	protein codi	GO:0040011	locomotion|	-	-	-	-	18:77789914	32	16	38	67	61	75	0.8328231	0.4014713	0.9804779	1.7120002	1.6045917	1.7203813	0.7382574	1.6789911	-1.185399	0.0008166	0.0427677	DOWN
ENSMUSG00	Adcy5	adenylate cy	protein codi	GO:0003824	catalytic acti	K08045	adenylate cy	mmu01100|	Metabolic pa	16:35154877	133	82	121	246	295	156	1.097216	0.6522284	0.9896464	1.9925362	2.459787	1.1343053	0.9130302	1.8622095	-1.028281	0.0008793	0.0453942	DOWN
ENSMUSG00	Ccl8	chemokine (	protein codi	GO:0032502	developmen	K16596	C-C motif ch	mmu04060|	Cytokine-cyt	11:82115185	12	12	19	25	60	41	1.4301261	1.37883	2.2449299	2.925294	7.2273845	4.3066873	1.6846286	4.8197886	-1.516539	0.000911	0.0463307	DOWN
ENSMUSG00	Rassf6	Ras associati	protein codi	GO:0023052	signaling|res	K09854	Ras associati	mmu04390|	Hippo signal	5:90603076-	38	81	100	150	150	213	0.409666	0.8419204	1.0688021	1.5876845	1.6344457	2.0238913	0.7734628	1.7486738	-1.176857	0.0009562	0.0471938	DOWN

Table S2-Down genes in IngWAT of HFD VS NCD

gene id	gene name	description	gene_type	GO_id	GO_term	KO_ID	KO_Descript	kegg_id	kegg_term	locus	ING H1 cou	ING H2 cou	ING H3 cou	ING N1 cou	ING N2 cou	ING N3 cou	ING H1 FPK	ING H2 FPK	ING H3 FPK	ING N1 FPK	ING N2 FPK	ING N3 FPK	H	N	log2FC	Pvalue	Qvalue	updown
ENSMUSG00	Mup10	major urinar	protein codi	GO:0005615	extracellular	-	-	-	-	4:60578260-	4	1	11	624	1031	773	0.1607436	0.0406735	0.4391046	27.350749	41.395019	30.240238	0.2135072	32.995335	-7.271834	3.84E-71	1.06E-66	DOWN
ENSMUSG00	Mup11	major urinar	protein codi	GO:0032502	developmen	-	-	-	-	4:60658466-	12	0	16	1152	2623	1778	0.3408381	0	0.4513951	35.68567	74.429423	49.158024	0.2640777	53.091039	-7.651362	5.90E-59	5.45E-55	DOWN
ENSMUSG00	Slc2a5	solute carrie	protein codi	GO:0050896	response to	K08143	MFS transpo	mmu04973	Carbohydrat	4:150119283	0	3	0	344	449	262	0	0.0462087	0	5.7099759	6.8269588	3.8814915	0.0154029	5.4728087	-8.472935	3.57E-51	2.47E-47	DOWN
ENSMUSG00	Elovl6	ELOVL family	protein codi	GO:0003824	catalytic acti	K10203	elongation o	mmu01100	Metabolic pa	3:129532355	370	316	548	5374	6910	5006	2.0328329	1.757131	2.990436	32.200801	37.927297	26.772015	2.2601333	32.300038	-3.837056	8.17E-47	4.53E-43	DOWN
ENSMUSG00	Mup16	major urinar	protein codi	GO:0032502	developmen	-	-	-	-	4:61515592-	0	0	3	179	314	249	0	0	0.14747	9.6617976	15.525282	11.995679	0.0491567	12.394253	-7.978069	2.12E-43	9.78E-40	DOWN
ENSMUSG00	Mup22	major urinar	protein codi	GO:0005615	extracellular	-	-	-	-	4:60817589-	20	26	87	5078	3723	3667	1.4106158	1.8559327	6.0946378	390.60583	262.32755	251.75505	3.1203954	301.56281	-6.594586	2.78E-43	1.10E-39	DOWN
ENSMUSG00	Acly	ATP citrate ly	protein codi	GO:0003824	catalytic acti	K01648	ATP citrate (	mmu01100	Metabolic pa	11:10047635	4295	4046	5350	36003	39453	26829	31.846298	30.362626	39.400689	291.1414	292.24706	193.63818	33.869871	259.00888	-2.934927	1.03E-33	2.86E-30	DOWN
ENSMUSG00	Cyp2f2	cytochrome	protein codi	GO:0003824	catalytic acti	K07416	cytochrome	mmu00980	Metabolism	7:27119909-	162	156	420	3951	4560	4053	4.3665038	4.2556011	11.244032	116.14352	122.7885	106.33734	6.6220457	115.08979	-4.119339	5.21E-31	1.31E-27	DOWN
ENSMUSG00	Mup18	major urinar	protein codi	GO:0032502	developmen	-	-	-	-	4:61670177-	2	0	2	66	113	95	0.1306155	0	0.1297311	4.7001058	7.3714161	6.0382383	0.0867822	6.0365867	-6.12019	2.60E-24	4.50E-21	DOWN
ENSMUSG00	Cyp2e1	cytochrome	protein codi	GO:0003824	catalytic acti	K07415	cytochrome	mmu01100	Metabolic pa	7:140763739	7482	5232	5865	24093	33605	28266	133.12546	94.216891	103.64914	467.52385	597.34005	489.5516	110.3305	518.1385	-2.231506	5.94E-23	8.65E-20	DOWN
ENSMUSG00	Cidea	cell death-in	protein codi	GO:0023052	signaling|me	-	-	-	-	18:67343604	81	81	80	394	590	407	4.411512	4.4648498	4.3276198	23.402969	32.101917	21.57693	4.4013272	25.693939	-2.545418	1.45E-20	2.01E-17	DOWN
ENSMUSG00	Pon1	paraoxonase	protein codi	GO:0003824	catalytic acti	-	-	-	-	6:5168090-5	36	46	73	314	470	389	0.6594678	0.8528418	1.328238	6.2732924	8.601379	6.9364436	0.9468492	7.2703716	-2.940823	1.38E-19	1.66E-16	DOWN
ENSMUSG00	Acss2	acyl-CoA syn	protein codi	GO:0003824	catalytic acti	K01895	acetyl-CoA s	mmu01100	Metabolic pa	2:155517948	2599	1997	2976	8921	11626	8992	12.329404	9.5880739	14.022423	46.155008	55.098583	41.522436	11.979967	47.592009	-1.990095	9.47E-19	1.01E-15	DOWN
ENSMUSG00	Tst	thiosulfate su	protein codi	GO:0032502	developmen	K01011	thiosulfate/3	mmu01100	Metabolic pa	15:78399556	229	277	509	1665	2304	1806	3.6818598	4.5074402	8.1283922	29.19554	37.007477	28.264498	5.4392307	31.489172	-2.533381	1.33E-18	1.32E-15	DOWN
ENSMUSG00	Mpzl2	myelin prote	protein codi	GO:0009987	cellular proc	-	-	-	-	9:45039799-	9	30	10	228	222	207	0.0646669	0.2181553	0.0713598	1.7865905	1.593482	1.447707	0.1180607	1.6092598	-3.768797	1.53E-18	1.46E-15	DOWN
ENSMUSG00	Rassf6	Ras associati	protein codi	GO:0023052	signaling|res	K09854	Ras associat	mmu04390	Hippo signa	5:90603076-	6	4	7	78	163	89	0.0644992	0.0435175	0.0747366	0.9144756	1.7505325	0.9313018	0.0609178	1.19877	-4.298548	2.06E-18	1.90E-15	DOWN
ENSMUSG00	Acaca	acetyl-Coenz	protein codi	GO:0003824	catalytic acti	K11262	acetyl-CoA c	mmu01100	Metabolic pa	11:84129672	2669	2512	5882	20373	26246	22175	7.995637	7.6162612	17.501845	66.562405	78.549238	64.663448	11.037915	69.92503	-2.663341	7.21E-18	6.05E-15	DOWN
ENSMUSG00	Sorl1	sortilin-relat	protein codi	GO:0032502	developmen	-	-	-	-	9:41964720-	643	551	521	2023	2174	1700	3.3065671	2.8677137	2.6610822	11.345714	11.168646	8.5095427	2.945121	10.341301	-1.812019	1.87E-17	1.52E-14	DOWN
ENSMUSG00	Gm45470	predicted ge	antisense	-	-	-	-	-	-	8:27228686-	483	495	639	1656	2139	1786	3.2260218	3.3461311	4.2391193	12.062853	14.272677	11.611608	3.6037574	12.649046	-1.811455	2.10E-17	1.62E-14	DOWN
ENSMUSG00	Gm5182	predicted ge	processed p	-	-	-	-	-	-	12:10152142	1107	1266	2363	9005	7237	8541	11.034872	12.772356	23.395794	97.897815	72.0697	82.87418	15.734341	84.280565	-2.421283	3.14E-17	2.29E-14	DOWN
ENSMUSG00	Tkt	transketolase	protein codi	GO:0003824	catalytic acti	K00615	transketolase	mmu01100	Metabolic pa	14:30548359	6509	4591	8659	22789	29153	28847	41.161108	29.383102	54.386956	157.16923	184.17486	177.56777	41.643722	172.97062	-2.054356	1.38E-16	9.53E-14	DOWN
ENSMUSG00	Mup17	major urinar	protein codi	GO:0005615	extracellular	-	-	-	-	4:61591929-	0	0	0	28	33	37	0	0	0	2.0004646	2.1596552	2.3593433	0	2.1731544	#NAME?	2.33E-16	1.58E-13	DOWN
ENSMUSG00	H2-Q10	histocompat	protein codi	GO:0002376	immune syst	K06751	MHC class I	mmu05168	Herpes simp	17:35470089	741	671	1504	3861	5867	4699	6.5414275	5.9950728	13.187315	37.172666	51.742193	40.378533	8.5746052	43.097797	-2.329472	3.38E-15	1.91E-12	DOWN
ENSMUSG00	Fasn	fatty acid syn	protein codi	GO:0032502	developmen	K00665	fatty acid syn	mmu01100	Metabolic pa	11:12080584	36699	35257	77824	224993	259590	229360	160.07824	155.6471	337.16764	1070.3272	1131.2025	973.83743	217.63099	1058.4557	-2.282005	7.00E-15	3.80E-12	DOWN
ENSMUSG00	Nabp1	nucleic acid	protein codi	GO:0023052	signaling|me	-	-	-	-	1:51465862-	502	476	340	2017	2358	1212	1.5211973	1.4598438	1.0233288	6.6658776	7.1383885	3.5749938	1.3347899	5.7930866	-2.11772	8.27E-15	4.32E-12	DOWN
ENSMUSG00	Pth1r	parathyroid	protein codi	GO:0032502	developmen	K04585	parathyroid	mmu04080	Neuroactive	9:110722085	247	138	224	785	1115	660	3.1934242	1.8057424	2.8764755	11.068741	14.401523	8.3060404	2.6252141	11.258768	-2.100542	3.94E-14	1.91E-11	DOWN
ENSMUSG00	Slc25a1	solute carrie	protein codi	GO:0009987	cellular proc	-	-	-	-	16:17925223	5364	4065	5029	12380	17968	13492	86.288215	66.182162	80.352361	217.19653	288.75985	211.26609	77.607579	239.07416	-1.623189	9.05E-14	4.18E-11	DOWN
ENSMUSG00	Gm7049	predicted ge	processed p	-	-	-	-	-	-	13:62813076	1366	1129	1240	3048	3698	3328	48.453658	40.531003	43.686968	117.91265	131.04401	114.90792	44.223876	121.28819	-1.455542	1.52E-13	6.69E-11	DOWN
ENSMUSG00	Me1	malic enzym	protein codi	GO:0003824	catalytic acti	K00029	malate dehy	mmu01100	Metabolic pa	9:86581371-	10974	8697	9053	23810	31567	26502	119.27954	95.672718	97.734479	282.24737	342.77463	280.39508	104.22891	301.80569	-1.533864	1.99E-13	8.37E-11	DOWN
ENSMUSG00	Slc43a1	solute carrie	protein codi	GO:0009987	cellular proc	-	-	-	-	2:84838850-	310	372	482	971	1531	1288	2.1254693	2.5813905	3.3824236	7.2607573	10.486792	8.5960648	2.6630945	8.7812048	-1.721315	4.77E-13	1.86E-10	DOWN
ENSMUSG00	Igkv14-126	immunoglob	IG_V_gene	GO:0002376	immune syst	-	-	-	-	6:67896172-	43	52	29	953	429	189	7.2939324	8.9273089	4.8859247	176.30279	72.698998	31.206851	7.035722	93.402879	-3.730697	6.86E-13	2.60E-10	DOWN
ENSMUSG00	Enpep	glutamyl am	protein codi	GO:0032502	developmen	K11141	glutamyl am	mmu04614	Renin-angio	3:129269175	898	886	810	2519	2115	2377	9.0136517	9.0006867	8.0753873	27.575406	21.208451	23.224381	8.6965753	24.002746	-1.46468	1.04E-12	3.80E-10	DOWN
ENSMUSG00	Adrb3	adrenergic re	protein codi	GO:0032502	developmen	K04143	adrenergic re	mmu04080	Neuroactive	8:27225776-	4756	4016	5782	11212	17085	14291	40.990088	35.030664	49.495925	105.38763	147.10464	119.89205	41.838892	124.12811	-1.568913	1.99E-12	6.97E-10	DOWN
ENSMUSG00	4930453N24	RIKEN cDNA	protein codi	GO:0009987	cellular proc	-	-	-	-	16:64751751	185	220	197	534	612	620	1.720959	2.0712833	1.8202044	5.4176371	5.6875496	5.6141219	1.8708156	5.5731029	-1.574813	3.47E-12	1.20E-09	DOWN
ENSMUSG00	Cyp1a1	cytochrome	protein codi	GO:0032502	developmen	K07408	cytochrome	mmu01100	Metabolic pa	9:57687928-	16	31	71	279	496	257	0.2405686	0.4717273	1.0602771	4.5748724	7.4500923	3.761224	0.5908577	5.2620629	-3.154746	7.11E-12	2.32E-09	DOWN
ENSMUSG00	Pygl	liver glycoge	protein codi	GO:0003824	catalytic acti	K00688	glycogen ph	mmu01100	Metabolic pa	12:70190817	2091	1779	2558	5194	5867	5875	15.924197	13.711878	19.348987	43.139482	44.636888	43.551394	16.328354	43.775921	-1.422758	9.63E-12	3.10E-09	DOWN
ENSMUSG00	Gm48832	predicted ge	lincRNA	-	-	-	-	-	-	9:85332352-	59	54	106	408	341	279	0.5394286	0.4996826	0.9625883	4.0682766	3.1146484	2.4829942	0.6672331	3.2219731	-2.271682	9.86E-12	3.14E-09	DOWN
ENSMUSG00	Celsr2	cadherin, EG	protein codi	GO:0032502	developmen	-	-	-	-	3:108390851	401	538	356	1027	1229	1602	1.7280314	2.346425	1.5237433	4.8266623	5.2909447	6.7198592	1.8660666	5.6124887	-1.58864	3.60E-11	1.08E-08	DOWN
ENSMUSG00	Gys2	glycogen syn	protein codi	GO:0003824	catalytic acti	K00693	glycogen syn	mmu01100	Metabolic pa	6:142422613	254	145	234	544	716	883	5.1917219	2.9995878	4.7505879	12.126771	14.620576	17.568247	4.3139659	14.771865	-1.775765	3.68E-11	1.10E-08	DOWN
ENSMUSG00	Acacb	acetyl-Coenz	protein codi	GO:0032502	developmen	K01946	acetyl-CoA c	mmu01100	Metabolic pa	5:114146535	2250	2635	3074	6296	9665	6315	10.472528	12.412729	14.211084	31.959742	44.941257	28.611004	12.365447	35.170667	-1.508058	4.33E-11	1.25E-08	DOWN
ENSMUSG00	Jchain	immunoglob	protein codi	GO:0002376	immune syst	-	-	-	-	5:88519809-	124	57	226	1214	1013	531	2.0613537	0.9590103	3.7315783	22.009904	16.823408	8.592422	2.2506474	15.808578	-2.812296	4.86E-11	1.36E-08	DOWN
ENSMUSG00	Baiap2l2	BAI1-associa	protein codi	GO:0032991	protein-cont	-	-	-	-	15:79258195	15	28	22	83	157	129	0.1902507	0.3594138	0.2771458	1.1480834	1.9892922	1.5925969	0.2756034	1.5766575	-2.516204	1.01E-10	2.65E-08	DOWN
ENSMUSG00	Parm1	prostate and	protein codi	GO:0003824	catalytic acti	-	-	-	-	5:91517615-	238	649	521	1756	2042	1615	2.8518053	7.870556	6.2006156	22.947594	24.444045	18.836762	5.6409923	22.076134	-1.968467	1.07E-10	2.77E-08	DOWN
ENSMUSG00	Gm7329	predicted ge	processed p	-	-	-	-	-	-	1:65095227-	166	124	126	365	468	415	48.002936	36.290977	36.189692	115.11243	135.20128	116.81506	40.161202	122.37626	-1.607449	1.93E-10	4.74E-08	DOWN
ENSMUSG00	Nat8l	N-acetyltran	protein codi	GO:0003824	catalytic acti	K18309	aspartate N-	mmu01100	Metabolic pa	5:33995984-	2338	1937	2172	4922	5119	4967	21.083903	17.678838	19.454526	48.40806	46.117529	43.600501	19.405755	46.04203	-1.246467	2.57E-10	6.08E-08	DOWN
ENSMUSG00	Angpt1	angiopoietin	protein codi	GO:0032502	developmen	K05465	angiopoietin	mmu04151	PI3K-Akt sig	15:42424723	244	251	391	810	811	847	2.55912	2.6643599	4.0731681	9.2652214	8.4976036	8.6471979	3.0988827	8.803341	-1.506303	2.86E-10	6.65E-08	DOWN
ENSMUSG00	Tmem79	transmembra	protein codi	GO:0032502	developmen	-	-	-	-	3:88328655-	47	37	84	278	252	218	0.4683578	0.3731625	0.8313977	3.0212841	2.5087178	2.1145845	0.5576393	2.5481955	-2.192072	3.41E-10	7.67E-08	DOWN
ENSMUSG00	Fam13a	family with s	protein codi	-	-	-	-	-	-	6:58932090-	680	989	1387	2583	3772	2862	3.7041787	5.4525107	7.5043545	15.345344	20.527157	15.175502	5.5536813	17.016001	-1.615376	3.95E-10	8.74E-08	DOWN
ENSMUSG00	Ighv11-2	immunoglob	IG_V_gene	GO:0002376	immune syst	-	-	-	-	12:11404824	12	19	11	319	148	59	1.5807199	2.5330908	1.4392342	45.828883	19.476617	7.5652377	1.851015	24.290246	-3.713989	4.10E-10	8.95E-08	DOWN
ENSMUSG00	Pdk1	pyruvate deh	protein codi	GO:0003824	catalytic acti	K12077	pyruvate deh	mmu04066	HIF-1 signal	2:71873224-	509	578	552	1164	1321	1311	2.7361146	3.1445735	2.9472011	6.8240008	7.0940431	6.8597751	2.9426297	6.9259397	-1.234904	5.65E-10	1.20E-07	DOWN
ENSMUSG00	Gm33432	predicted ge	lincRNA	-	-	-	-	-	-	15:84127304	16	20	14	70	87	84	0.615752	0.7789686	0.5351284	2.9379243	3.3447816	3.1466139	0.643283	3.1431066	-2.288666	6.01E-10	1.27E-07	DOWN
ENSMUSG00	2810459M1	RIKEN cDNA	protein codi	GO:0005515	protein bind	-	-	-	-	1:86045863-	15	7	18	56	107	93	0.2368488	0.1118573	0.2822865	0.9643425	1.6878289	1.4293726	0.2103309	1.3605147	-2.69342	8.28E-10	1.72E-07	DOWN
ENSMUSG00	Gm42517	predicted ge	protein codi	GO:0031224	intrinsic com	-	-	-	-	5:108366073	44	39	46	119	203	178	1.4443049	1.295658	1.499751	4.2601526	6.6570088	5.6874563	1.413238	5.5348726	-1.969546	1.06E-09	2.19E-07	DOWN
ENSMUSG00	Prtn3	proteinase 3	protein codi	GO:0032502	developmen	-	-	-	-	10:79874476	74	74	112	241	313	292	1.5181774	1.5365217	2.2822307	5.3923115	6.4151487	5.8312501	1.7789766	5.8795701	-1.724663	2.08E-09	4.15E-07	DOWN
ENSMUSG00	Cnr1	cannabinoid	protein codi	GO:0032502	developmen	K04277	cannabinoid	mmu04080	Neuroactive	4:33924593-	207	162	181	439	484	505	1.0199134	0.8078389	0.8857778	2.358994	2.3823877	2.422005	0.9045101	2.3877956	-1.400471	2.32E-09	4.56E-07	DOWN
ENSMUSG00	Cfd	complement	protein codi	GO:0002376	immune syst	K01334	complement	mmu05171	Coronavirus	10:79890853	64350	54427	80171	136255	195382	153673	3103.8627	2656.9659	3840.834	7167.6353	9414.8379	7215.0983	3200.5542	7932.5238	-1.309458	2.94E-09	5.66E-07	DOWN
ENSMUSG00	Gbe1	glucan (1,4-a	protein codi	GO:0003824	catalytic acti	K00700	1,4-alpha-gl	mmu01100	Metabolic pa	16:70313949	770	761	1206	2076	2246	2451	4.8305356	4.831776	7.5146004	14.203691	14.076298	14.967126	5.7256373	14.415705	-1.332133	3.59E-09	6.77E-07	DOWN
ENSMUSG00	Ces1f	carboxyleste	protein codi	GO:0003824	catalytic acti	K01044	carboxyleste	mmu00983	Drug metab	8:93256236-	1725	1614	1796	3627	3659	3745	25.302785	23.960755	26.166164	58.0225	53.618678	53.47144	25.143235	55.037539	-1.130246	3.93E-09	7.30E-07	DOWN
ENSMUSG00	AA914427	EST AA91442	TEC	-	-	-	-	-	-	16:17922043	27	27	22	132	228	75	1.4062027	1.4231695	1.1380566	7.497651	11.862896	3.8021841	1.3224763	7.7209104	-2.545529	5.12E-09	9.32E-07	DOWN
ENSMUSG00	Aspg	asparaginase	protein codi	GO:0003824	catalytic acti	-	-	-	-	12:11210667	12	19	25	78	139	76	0.1558075	0.2496803	0.3224117	1.104527	1.8030294	0.9605454	0.2426332	1.2893673	-2.409814	6.88E-09	1.22E-06	DOWN
ENSMUSG00	Ighv7-3	immunoglob	IG_V_gene	GO:0002376	immune syst	-	-	-	-	12:11415318	0	0	0	26	41	6	0	0	0	4.7699656	6.8901114	0.9825246	0	4.2142005	#NAME?	7.55E-09	1.33E-06	DOWN
ENSMUSG00	Dennd2d	DENN/MADD	protein codi	GO:0003824	catalytic acti	-	-	-	-	3:106482405	90	95	205	402	437	545	0.6713426	0.717206	1.518835	3.2703769	3.2565497	3.9572093	0.9691279	3.494712	-1.850415	9.93E-09	1.69E-06	DOWN
ENSMUSG00	Igkc	immunoglob	IG_C_gene	-	-	-	-	-	-	6:70726435-	986	265	1015	4890	3550	2523	112.54985	30.614756	115.07693	608.76046	404.82815	280.33412	86.080514	431.30758	-2.324958	1.19E-08	2.00E-06	DOWN
ENSMUSG00	Pdhb	pyruvate deh	protein codi	GO:0003824	catalytic acti	K00162	pyruvate deh	mmu01100	Metabolic pa	14:8165996-	2434	2218	3263	5572	6165	5997	20.006608	18.451515	26.639383	49.949759	50.624438	47.981918	21.699168	49.518705	-1.190334	1.39E-08	2.31E-06	DOWN
ENSMUSG00	Iglc1	immunoglob	IG_C_gene	GO:0002376	immune syst	-	-	-	-	16:19061618	10	6	15	106	171	32	1.3375319	0.812215	1.9927938	15.463224	22.850414	4.1663965	1.3808469	14.160011	-3.358197	2.40E-08	3.83E-06	DOWN
ENSMUSG00	Mup3	major urinar	protein codi	GO:0036094	small molecu	-	-	-	-	4:62083476-	12	1	14	47	91	75	0.4178394	0.0352395	0.484193	1.7848421	3.1655564	2.5420565	0.312424	2.497485	-2.998899	2.58E-08	4.04E-06	DOWN
ENSMUSG00	Mogat1	monoacylgly	protein codi	GO:0003824	catalytic acti	K14458	2-acylglycer	mmu00561	Glycerolipid	1:78510991-	216	199	303	507	617	660	4.5992278	4.2884527	6.4080666	11.773553	13.124709	13.679338	5.0985824	12.8592	-1.334633	2.60E-08	4.05E-06	DOWN
ENSMUSG00	Slc1a3	solute carrie	protein codi	GO:0032502	developmen	K05614	solute carrie	mmu05016	Huntington	15:8634124-	2141	2180	2343	4221	4986	4521	11.096344	11.435033	12.061181	23.858772	25.816053	22.808068	11.530853	24.160964	-1.067179	2.73E-08	4.23E-06	DOWN
ENSMUSG00	Gsta3	glutathione S	protein codi	GO:0032502	developmen	K00799	glutathione S	mmu01100	Metabolic pa	1:21240589-	919	580	989	2317	2219	1657	11.746539	7.503086	12.555823	32.299088	28.3352	20.616162	10.601816	27.083484	-1.353102	3.17E-08	4.82E-06	DOWN
ENSMUSG00	Thrsp	thyroid horm	protein codi	GO:0008152	metabolic pr	-	-	-	-	7:97412938-	6474	5093	14097	32699	31462	23542	146.86016	116.92911	317.62327	808.97535	713.00404	519.83485	193.80418	680.60475	-1.812218	3.22E-08	4.88E-06	DOWN
ENSMUSG00	Ighv6-3	immunoglob	IG_V_gene	GO:0002376	immune syst	-	-	-	-	12:11439173	2	0	0	116	10	27	0.3412146	0	0	21.58035	1.7041906	4.483232	0.1137382	9.2559243	-6.346588	3.57E-08	5.36E-06	DOWN
ENSMUSG00	Gbp11	guanylate bin	polymorphic	GO:0002376	immune syst	-	-	-	-	5:105323042	58	65	69	196	204	168	1.1514051	1.305958	1.3605129	4.2435065	4.0457954	3.2463922	1.2726253	3.8452314	-1.595263	4.30E-08	6.33E-06	DOWN
ENSMUSG00	P2rx5	purinergic re	protein codi	GO:0032502	developmen	K05219	P2X purinoc	mmu04080	Neuroactive	11:73160421	63	79	60	182	222	196	1.5038396	1.9085661	1.4225519	4.7380909	5.2940684	4.5541724	1.6116526	4.8621105	-1.593042	4.77E-08	6.99E-06	DOWN
ENSMUSG00	Mup19	major urinar	protein codi	GO:0032502	developmen	-	-	-	-	4:61778324-	0	2	3	29	24	16	0	0.0925566	0.1362543	1.4462614	1.0963777	0.7121777	0.0762703	1.0849389	-3.830349	6.49E-08	9.32E-06	DOWN
ENSMUSG00	Slc5a6	solute carrie	protein codi	GO:0009987	cellular proc	K14386	solute carrie	mmu04977	Vitamin dige	5:31036036-	294	332	409	573	992	952	3.0102217	3.4403897	4.1593858	6.3984609	10.146998	9.4880995	3.5366657	8.6778528	-1.294948	7.22E-08	1.03E-05	DOWN
ENSMUSG00	Acsm3	acyl-CoA syn	protein codi	GO:0003824	catalytic acti	K01896	medium-cha	mmu01100	Metabolic pa	7:119760923	117	91	170	308	448	330	0.9151253	0.7203689	1.3206755	2.6273325	3.5006292	2.5124595	0.9853899	2.8801404	-1.547373	1.30E-07	1.77E-05	DOWN
ENSMUSG00	Oscp1	organic solu	protein codi	GO:0050896	response to	-	-	-	-	4:126058569	97	101	142	248	321	359	0.9195274	0.9690165	1.337015	2.5639715	3.0399891	3.3126695	1.0751863	2.97221	-1.466949	1.44E-07	1.94E-05	DOWN
ENSMUSG00	Gpd2	glycerol pho	protein codi	GO:0032502	developmen	K00111	glycerol-3-p	mmu00564	Glycerophos	2:57237635-	636	1253	1097	2402	2312	2502	4.9100126	9.7902673	8.4117563	20.224053	17.831499	18.801985	7.7040121	18.952512	-1.298707	1.59E-07	2.14E-05	DOWN
ENSMUSG00	Gm454	predicted ge	transcribed	-	-	-	-	-	-	5:138202134	222	140	174	371	433	547	10.28326	6.5633008	8.0053583	18.742204	20.037304	24.663531	8.283973	21.14768	-1.352105	2.26E-07	2.99E-05	DOWN
ENSMUSG00	Mmp9	matrix metal	protein codi	GO:0032502	developmen	K01403	matrix metal	mmu05200	Pathways in	2:164940780	294	271	358	1125	552	777	4.5267812	4.2230873	5.4749448	18.89143	8.490946	11.645396	4.7416044	13.009257	-1.456091	2.30E-07	3.02E-05	DOWN
ENSMUSG00	Mapk8ip1	mitogen-act	protein codi	GO:0002376	immune syst	K04434	mitogen-act	mmu04010	MAPK signal	2:92383676-	317	269	290	570	783	570	3.1729642	2.7250603	2.8830837	6.2222835	7.8296376	5.5535559	2.9270361	6.535159	-1.158782	2.52E-07	3.28E-05	DOWN
ENSMUSG00	Gm37310	predicted ge	TEC	-	-	-	-	-	-	15:8652106-	12	37	20	81	159	91	0.2455229	0.7661901	0.4064434	1.807463	3.2500314	1.8123715	0.4727188	2.2899553	-2.276265	3.37E-07	4.30E-05	DOWN
ENSMUSG00	Gpx8	glutathione p	protein codi	GO:0016209	antioxidant a	K00432	glutathione p	mmu01100	Metabolic pa	13:11304275	380	325	419	694	898	734	12.74221	11.029679	13.955002	25.379915	30.082348	23.957865	12.57563	26.473376	-1.073912	3.54E-07	4.48E-05	DOWN
ENSMUSG00	Gsta4	glutathione S	protein codi	GO:0003824	catalytic acti	K00799	glutathione S	mmu01100	Metabolic pa	9:78182774-	248	213	395	606	676	747	10.546393	9.1674607	16.684093	28.105639	28.719203	30.921672	12.132649	29.248838	-1.269485	5.94E-07	7.03E-05	DOWN
ENSMUSG00	Pcx	pyruvate car	protein codi	GO:0016032	viral process	K01958	pyruvate car	mmu01100	Metabolic pa	19:4510472-	17690	13087	19544	31344	34833	36294	166.16479	124.41389	182.33872	321.09613	326.87099	331.8461	157.63913	326.60441	-1.050919	6.03E-07	7.10E-05	DOWN
ENSMUSG00	Gm6123	predicted ge	processed p	-	-	-	-	-	-	1:4771131-4	273	237	332	538	606	643	15.508335	13.625984	18.732429	33.331414	34.391353	35.555262	15.955583	34.42601	-1.109438	7.53E-07	8.69E-05	DOWN
ENSMUSG00	Tmem53	transmembra	protein codi	GO:0031224	intrinsic com	-	-	-	-	4:117251951	138	127	194	291	438	405	2.4554085	2.2869845	3.4284643	5.6468456	7.7855998	7.014381	2.7236191	6.8156088	-1.323318	8.83E-07	0.0001006	DOWN
ENSMUSG00	Gm19522	predicted ge	antisense	-	-	-	-	-	-	16:42884370	30	36	45	194	108	102	0.7214969	0.8762751	1.074948	5.088506	2.5948796	2.3878722	0.8909067	3.3570859	-1.913863	9.17E-07	0.0001036	DOWN
ENSMUSG00	Ptges	prostaglandi	protein codi	GO:0016209	antioxidant a	K15729	microsomal	mmu01100	Metabolic pa	2:30889471-	618	503	1391	2292	2701	2110	7.140238	5.8818008	15.96268	28.880749	31.17621	23.729992	9.6615728	27.928984	-1.531433	1.01E-06	0.0001138	DOWN
ENSMUSG00	Mup12	major urinar	protein codi	GO:0032502	developmen	-	-	-	-	4:60737383-	0	2	0	18	9	18	0	0.1327378	0	1.2873968	0.5896445	1.1490501	0.0442459	1.0086971	-4.510804	1.17E-06	0.0001303	DOWN
ENSMUSG00	Sfxn1	sideroflexin 1	protein codi	GO:0032502	developmen	-	-	-	-	13:54071869	1592	1454	2653	3634	4387	4466	9.4540289	8.7388837	15.648235	23.535763	26.026506	25.815666	11.280382	25.125978	-1.155364	1.35E-06	0.0001467	DOWN
ENSMUSG00	BC035947	cDNA seque	protein codi	GO:0003824	catalytic acti	-	-	-	-	1:78497026-	69	64	117	207	231	255	0.6906418	0.6483382	1.1631776	2.2596763	2.309893	2.4844881	0.8340525	2.3513525	-1.495281	1.36E-06	0.0001467	DOWN
ENSMUSG00	Igkv17-121	immunoglob	IG_V_gene	GO:0002376	immune syst	-	-	-	-	6:68036812-	0	4	3	74	26	13	0	0.3467284	0.2552125	6.9124493	2.2247553	1.083855	0.200647	3.4070199	-4.085779	1.43E-06	0.0001543	DOWN
ENSMUSG00	Gm14286	predicted ge	antisense	-	-	-	-	-	-	2:157544266	40	40	48	83	158	159	5.2350872	5.2983296	6.2396322	11.847033	20.658216	20.255826	5.5910163	17.587025	-1.653329	1.56E-06	0.0001671	DOWN
ENSMUSG00	Orm3	orosomucoi	protein codi	GO:0002376	immune syst	-	-	-	-	4:63356162-	11	7	26	48	94	73	0.8641341	0.5565371	2.0287391	4.1125084	7.3773794	5.5822746	1.1498035	5.6907208	-2.307224	1.67E-06	0.0001776	DOWN
ENSMUSG00	Dixdc1	DIX domain	protein codi	GO:0032502	developmen	-	-	-	-	9:50662752-	201	215	190	474	415	398	0.7492528	0.8111257	0.7034589	1.9269899	1.5454474	1.4441266	0.7546125	1.6388546	-1.11888	2.51E-06	0.0002583	DOWN
ENSMUSG00	Igfals	insulin-like g	protein codi	GO:0032991	protein-cont	K17256	insulin-like g	mmu04935	Growth horr	17:24865997	319	552	281	678	1341	888	2.2887342	4.0083044	2.0024612	5.3052186	9.6118537	6.2016576	2.7664999	7.0395766	-1.347427	2.99E-06	0.0003012	DOWN
ENSMUSG00	Unc93a2	unc-93 hom	protein codi	GO:0031224	intrinsic com	-	-	-	-	17:7362815-	18	18	40	57	116	160	0.2937565	0.2973118	0.648387	1.0145299	1.891266	2.5417435	0.4131518	1.8158465	-2.135899	3.05E-06	0.0003045	DOWN
ENSMUSG00	Mpc1	mitochondri	protein codi	GO:0008152	metabolic pr	K22138	mitochondri	mmu05415	Diabetic car	17:8282904-	223	233	185	334	551	557	3.3037396	3.4936195	2.7222522	5.3965764	8.1550646	8.0324397	3.1732038	7.1946936	-1.180993	3.23E-06	0.0003206	DOWN
ENSMUSG00	Aacs	acetoacetyl-	protein codi	GO:0032502	developmen	K01907	acetoacetyl-	mmu01100	Metabolic pa	5:125475814	3230	2638	3577	7980	5944	5352	41.653692	34.430506	45.816745	112.23373	76.578025	67.182753	40.633648	85.331503	-1.070404	3.38E-06	0.0003312	DOWN
ENSMUSG00	Igkv10-94	immunoglob	IG_V_gene	GO:0002376	immune syst	-	-	-	-	6:68704508-	0	0	3	48	18	8	0	0	0.5214573	9.1612939	3.1470366	1.3627839	0.1738191	4.5570381	-4.712438	3.41E-06	0.0003327	DOWN
ENSMUSG00	Gfra4	glial cell line	protein codi	GO:0032502	developmen	-	-	-	-	2:131039632	35	58	57	104	158	182	0.4310405	0.7229207	0.697227	1.3968425	1.9439084	2.1817557	0.6170628	1.8408355	-1.576872	3.80E-06	0.0003675	DOWN
ENSMUSG00	Serpina3k	serine (or cys	protein codi	GO:0005615	extracellular	-	-	-	-	12:10433848	14	6	8	38	56	30	0.4133127	0.1792638	0.234585	1.2234963	1.651613	0.8621047	0.2757205	1.245738	-2.175722	4.99E-06	0.0004579	DOWN
ENSMUSG00	Alb	albumin [Sou	protein codi	GO:0007610	behavior|rhy	K16141	serum albun	mmu04918	Thyroid horr	5:90460897-	260	167	561	910	929	917	6.160344	4.0046442	13.202251	23.514833	21.989789	21.149066	7.7890796	22.217896	-1.512197	6.28E-06	0.0005559	DOWN
ENSMUSG00	Igkv14-111	immunoglob	IG_V_gene	GO:0002376	immune syst	-	-	-	-	6:68256389-	7	0	8	151	65	11	1.1551417	0	1.3112536	27.17553	10.715703	1.7668609	0.8221318	13.219365	-4.007139	8.14E-06	0.0007023	DOWN
ENSMUSG00	Ppp1r1a	protein phos	protein codi	GO:0003824	catalytic acti	K08050	protein phos	mmu04261	Adrenergic s	15:10353027	702	702	1235	1455	1942	2155	24.194174	24.486613	42.276103	54.689796	66.864778	72.295594	30.318963	64.616723	-1.091687	8.66E-06	0.0007312	DOWN
ENSMUSG00	Gm34280	predicted ge	antisense	-	-	-	-	-	-	7:99694297-	14	21	27	47	79	84	0.4021685	0.6105346	0.7703458	1.4724525	2.2671408	2.348797	0.5943496	2.0294634	-1.771715	8.97E-06	0.0007546	DOWN
ENSMUSG00	Rorc	RAR-related	protein codi	GO:0032502	developmen	K08534	RAR-related	mmu04659	Th17 cell dif	3:94372794-	190	337	283	552	602	546	2.1035737	3.7761603	3.1120295	6.6651713	6.6584561	5.884187	2.9972545	6.4026048	-1.095017	1.06E-05	0.0008576	DOWN
ENSMUSG00	Rhbg	Rhesus bloo	protein codi	GO:0009987	cellular proc	-	-	-	-	3:88242874-	4	12	28	56	82	71	0.1253916	0.3807701	0.8718974	1.9147452	2.5682599	2.1667029	0.459353	2.2165694	-2.270653	1.08E-05	0.0008708	DOWN
ENSMUSG00	Pcdhb7	protocadher	protein codi	GO:0009987	cellular proc	-	-	-	-	18:37341702	23	19	17	55	71	52	0.3989396	0.3335489	0.2928816	1.0404435	1.230319	0.8779814	0.34179	1.0495813	-1.618632	1.40E-05	0.0010823	DOWN
ENSMUSG00	Igkv4-69	immunoglob	IG_V_gene	GO:0002376	immune syst	-	-	-	-	6:69283794-	0	0	0	0	7	124	0	0	0	0	1.2202553	21.062606	0	7.4276204	#NAME?	1.74E-05	0.0013229	DOWN
ENSMUSG00	AI838599	expressed se	lincRNA	-	-	-	-	-	-	4:3242055-3	46	44	53	105	186	99	0.5745447	0.5562023	0.6575041	1.4302828	2.3208815	1.2036209	0.5960837	1.651595	-1.470273	2.10E-05	0.0015438	DOWN
ENSMUSG00	Cntnap1	contactin ass	protein codi	GO:0032502	developmen	K07379	contactin as	mmu04514	Cell adhesio	11:10117052	174	203	267	503	390	436	1.7740841	2.094778	2.7038951	5.5932322	3.9725006	4.3271495	2.1909191	4.6309608	-1.079775	2.48E-05	0.0017756	DOWN
ENSMUSG00	Mup15	major urinar	protein codi	GO:0005615	extracellular	-	-	-	-	4:61435790-	0	0	4	23	12	12	0	0	0.2608015	1.6467893	0.7870214	0.7668422	0.0869338	1.0668843	-3.617342	2.53E-05	0.0018037	DOWN
ENSMUSG00	Gm49322	predicted ge	protein codi	GO:0003824	catalytic acti	-	-	-	-	10:80606021	57	48	68	226	68	521	0.5845992	0.4982411	0.6927062	2.5279159	0.6967341	5.2013145	0.5918489	2.8086549	-2.246579	2.63E-05	0.0018589	DOWN
ENSMUSG00	Igkv2-109	immunoglob	IG_V_gene	GO:0002376	immune syst	-	-	-	-	6:68302427-	4	4	0	34	8	34	0.6494212	0.6573006	0	6.0208175	1.2976734	5.3737765	0.4355739	4.2307558	-3.279926	3.39E-05	0.0022988	DOWN
ENSMUSG00	Rprd1b	regulation of	protein codi	GO:0008152	metabolic pr	-	-	-	-	2:158028497	167	198	158	560	558	219	1.1451392	1.3741203	1.0761001	4.1879283	3.8225291	1.4617602	1.1984532	3.1574059	-1.397566	3.68E-05	0.0024575	DOWN
ENSMUSG00	Ddo	D-aspartate	protein codi	GO:0003824	catalytic acti	K00272	D-aspartate	mmu01100	Metabolic pa	10:40630011	79	126	106	243	214	236	0.4607592	0.7437592	0.6140522	1.5456891	1.2469055	1.3398237	0.6061902	1.3774728	-1.184181	3.81E-05	0.0025315	DOWN
ENSMUSG00	Rtn4rl2	reticulon 4 re	protein codi	GO:0032502	developmen	-	-	-	-	2:84871924-	161	105	115	196	372	316	3.7118439	2.4500191	2.6333897	4.9282029	8.5680304	7.091556	2.9317509	6.8625964	-1.226992	3.89E-05	0.0025691	DOWN
ENSMUSG00	Hcar2	hydroxycarb	protein codi	GO:0002376	immune syst	K08402	hydroxycarb	mmu04024	cAMP signal	5:123863570	407	218	326	530	797	608	12.806101	6.9421875	10.188122	18.187281	25.05273	18.621585	9.9788034	20.620532	-1.047143	4.16E-05	0.0027142	DOWN
ENSMUSG00	Ighv4-1	immunoglob	IG_V_gene	GO:0002376	immune syst	-	-	-	-	12:11394828	0	0	0	19	31	0	0	0	0	3.0394537	4.5427475	0	0	2.5274004	#NAME?	4.72E-05	0.0030443	DOWN
ENSMUSG00	Igkv4-91	immunoglob	IG_V_gene	GO:0002376	immune syst	-	-	-	-	6:68768555-	4	5	0	97	55	3	0.6784456	0.8583464	0	17.944711	9.3203547	0.495304	0.512264	9.2534566	-4.175033	4.89E-05	0.0031265	DOWN
ENSMUSG00	Rps18-ps4	ribosomal pr	lincRNA	-	-	-	-	-	-	13:97751112	0	0	0	952	0	0	0	0	0	58.760606	0	0	0	19.586869	#NAME?	5.07E-05	0.0032131	DOWN
ENSMUSG00	Strip2	striatin intera	protein codi	GO:0032502	developmen	-	-	-	-	6:29917012-	176	181	143	280	390	328	0.8117817	0.8449316	0.6551127	1.4084899	1.7970693	1.4726179	0.7706087	1.5593924	-1.016914	5.26E-05	0.0033013	DOWN
ENSMUSG00	Gpr146	G protein-co	protein codi	GO:0023052	signaling|res	-	-	-	-	5:139377697	366	338	180	283	1140	1492	1.5377029	1.4372282	0.7511357	1.2967245	4.7848779	6.1017051	1.2420223	4.0611025	-1.70918	6.48E-05	0.0039531	DOWN
ENSMUSG00	Igkv2-112	immunoglob	IG_V_gene	GO:0002376	immune syst	-	-	-	-	6:68219981-	0	4	1	25	10	13	0	0.3669111	0.0899936	2.4712734	0.9055102	1.1469451	0.1523016	1.5079096	-3.307547	6.48E-05	0.0039531	DOWN
ENSMUSG00	Igkv15-103	immunoglob	IG_V_gene	GO:0002376	immune syst	-	-	-	-	6:68437440-	7	0	8	19	24	39	1.1335791	0	1.2867768	3.3555569	3.8826387	6.1476256	0.8067853	4.4619404	-2.467415	6.53E-05	0.0039756	DOWN
ENSMUSG00	Igkv8-24	immunoglob	IG_V_gene	GO:0002376	immune syst	-	-	-	-	6:70216858-	4	0	0	26	24	5	0.6654343	0	0	4.717692	3.9890124	0.8097731	0.2218114	3.1721592	-3.838059	8.94E-05	0.0052566	DOWN
ENSMUSG00	Gm16323	predicted ge	antisense	-	-	-	-	-	-	2:30413716-	4	7	11	22	29	30	0.4368409	0.7737468	1.1933219	2.6205241	3.1642724	3.1894773	0.8013032	2.9914246	-1.900413	9.88E-05	0.0057497	DOWN
ENSMUSG00	Thrb	thyroid horm	protein codi	GO:0032502	developmen	K08362	thyroid horm	mmu04080	Neuroactive	14:17660261	63	254	80	348	410	470	0.3287307	1.3413831	0.4146156	1.9803875	2.1372692	2.3872052	0.6949098	2.1682873	-1.641658	9.97E-05	0.0057764	DOWN
ENSMUSG00	Gm50241	predicted ge	protein codi	GO:0005654	nucleoplasm	-	-	-	-	8:106883890	10	10	6	14	6	709	0.1959469	0.1983338	0.1167768	0.2991879	0.1174659	13.523689	0.1703525	4.6467809	-4.769637	0.0001003	0.0058005	DOWN
ENSMUSG00	Nrn1	neuritin 1 [So	protein codi	GO:0032502	developmen	-	-	-	-	13:36725361	37	30	47	85	91	94	0.4077067	0.3345761	0.5143958	1.0215018	1.0017656	1.0082449	0.4188929	1.0105041	-1.270422	0.0001107	0.0061922	DOWN
ENSMUSG00	Ighv1-11	immunoglob	IG_V_gene	GO:0002376	immune syst	-	-	-	-	12:11461224	0	0	0	7	4	7	0	0	0	1.3208654	0.6913531	1.1788086	0	1.0636757	#NAME?	0.0001112	0.0062082	DOWN
ENSMUSG00	Egln3	egl-9 family	protein codi	GO:0003824	catalytic acti	K09592	hypoxia-ind	mmu05200	Pathways in	12:54178981	43	32	40	120	73	96	0.980927	0.7388295	0.9063291	2.9855322	1.6636772	2.1317377	0.8753619	2.2603157	-1.368573	0.0001206	0.0066943	DOWN
ENSMUSG00	Pcsk4	proprotein c	protein codi	GO:0032502	developmen	-	-	-	-	10:80321283	87	98	113	131	306	263	0.8992771	1.0252165	1.1601289	1.4767647	3.1598624	2.6461745	1.0282075	2.4276005	-1.2394	0.0001319	0.0072064	DOWN
ENSMUSG00	Npas2	neuronal PA	protein codi	GO:0008152	metabolic pr	K09026	neuronal PA	mmu04710	Circadian rhy	1:39193731-	82	22	26	270	161	83	1.0082146	0.2737655	0.3175168	3.6205461	1.977606	0.9933656	0.5331656	2.1971726	-2.042993	0.000137	0.0074539	DOWN
ENSMUSG00	Rprml	reprimo-like	protein codi	GO:0031224	intrinsic com	-	-	-	-	11:10364957	97	118	124	155	364	253	5.8321708	7.1805541	7.4051341	10.163902	21.86422	14.80707	6.805953	15.611731	-1.197761	0.0001568	0.0083167	DOWN
ENSMUSG00	Ly6h	lymphocyte a	protein codi	GO:0023052	signaling|res	-	-	-	-	15:75551268	112	73	97	137	304	216	0.9365757	0.6178252	0.8056561	1.249437	2.539635	1.7581974	0.7866857	1.8490898	-1.232956	0.0001601	0.008412	DOWN
ENSMUSG00	Gm16537	predicted ge	antisense	-	-	-	-	-	-	15:99715543	14	16	10	40	31	50	1.2110887	1.4008291	0.8591634	3.7737049	2.6790562	4.2102057	1.1570271	3.5543223	-1.619152	0.0001589	0.008412	DOWN
ENSMUSG00	Gm28875	predicted ge	lincRNA	-	-	-	-	-	-	12:10492550	142	122	111	194	344	242	7.252475	6.3062994	5.6308722	10.806121	17.552176	12.031065	6.3965489	13.463121	-1.073647	0.0001599	0.008412	DOWN
ENSMUSG00	A530020G2	RIKEN cDNA	antisense	-	-	-	-	-	-	3:121531619	530	651	1291	1506	1732	1779	6.6170154	8.2259237	16.009092	20.505989	21.602719	21.619856	10.28401	21.242855	-1.046575	0.0001652	0.0086132	DOWN
ENSMUSG00	Ifi44	interferon-in	protein codi	GO:0002376	immune syst	-	-	-	-	3:151730922	73	65	65	99	195	163	0.5897391	0.5314521	0.5215602	0.8722469	1.5737764	1.2817808	0.5475838	1.2426014	-1.182212	0.0001787	0.0092164	DOWN
ENSMUSG00	Gm43597	predicted ge	lincRNA	-	-	-	-	-	-	5:150599932	46	76	37	108	186	113	0.4350469	0.7274598	0.3475631	1.1139661	1.7573782	1.0402713	0.5033566	1.3038719	-1.373149	0.0001817	0.0093148	DOWN
ENSMUSG00	Ccdc42	coiled-coil d	protein codi	GO:0032502	developmen	-	-	-	-	11:68587021	2	9	18	25	51	48	0.0491592	0.223844	0.4393663	0.6700741	1.2521336	1.1482461	0.2374565	1.0234846	-2.107754	0.0001868	0.009506	DOWN
ENSMUSG00	Igkv8-28	immunoglob	IG_V_gene	GO:0002376	immune syst	-	-	-	-	6:70143593-	0	5	2	92	12	6	0	0.4552415	0.1787407	9.0267496	1.0785108	0.5254687	0.2113274	3.5435764	-4.067654	0.0001881	0.0095377	DOWN
ENSMUSG00	Gm43413	predicted ge	TEC	-	-	-	-	-	-	5:123110696	38	41	34	91	78	86	1.4735684	1.6091204	1.3095509	3.8485642	3.0217462	3.2462014	1.4640799	3.3721706	-1.203683	0.0002133	0.0105472	DOWN
ENSMUSG00	Gm26671	predicted ge	lincRNA	-	-	-	-	-	-	3:57612697-	17	20	24	65	54	42	0.3917767	0.4664943	0.5493607	1.6337359	1.243266	0.9421929	0.4692106	1.2730649	-1.439999	0.0002328	0.0112702	DOWN
ENSMUSG00	Ighv7-1	immunoglob	IG_V_gene	GO:0002376	immune syst	-	-	-	-	12:11389640	2	0	0	3	10	26	0.3346347	0	0	0.547321	1.6713274	4.2339019	0.1115449	2.1508501	-4.26921	0.0002345	0.0112909	DOWN
ENSMUSG00	Gm43218	predicted ge	IG_V_gene	GO:0002376	immune syst	-	-	-	-	6:70240427-	0	0	0	14	0	18	0	0	0	2.6953	0	3.0930825	0	1.9294609	#NAME?	0.0003012	0.0138533	DOWN
ENSMUSG00	Igkv6-20	immunoglob	IG_V_gene	GO:0002376	immune syst	-	-	-	-	6:70335841-	0	0	1	15	5	5	0	0	0.1638869	2.6492051	0.8089225	0.7881792	0.054629	1.4154356	-4.695436	0.0003029	0.0139099	DOWN
ENSMUSG00	Igkv19-93	immunoglob	IG_V_gene	GO:0002376	immune syst	-	-	-	-	6:68736297-	4	1	0	62	18	2	0.3435411	0.0869273	0	5.8079441	1.5445851	0.1672537	0.1434895	2.5065943	-4.126712	0.0003269	0.0148129	DOWN
ENSMUSG00	Ighv1-58	immunoglob	IG_V_gene	GO:0002376	immune syst	-	-	-	-	12:11531216	0	0	0	40	1	2	0	0	0	7.5474097	0.1728803	0.33689	0	2.6857267	#NAME?	0.0003346	0.0150402	DOWN
ENSMUSG00	Gm45727	predicted ge	unprocessed	-	-	-	-	-	-	8:93238653-	6	3	6	13	24	20	0.6369859	0.3223301	0.6326772	1.5052077	2.5454362	2.0668095	0.5306644	2.0391511	-1.942097	0.0003541	0.0155656	DOWN
ENSMUSG00	Ppp4r4	protein phos	protein codi	GO:0032502	developmen	-	-	-	-	12:10353228	79	67	87	126	185	155	0.9656652	0.8288729	1.0562567	1.6797185	2.2591475	1.8442553	0.9502649	1.9277071	-1.020484	0.000387	0.0166268	DOWN
ENSMUSG00	Ighv1-39	immunoglob	IG_V_gene	GO:0002376	immune syst	-	-	-	-	12:11491459	7	0	3	41	51	4	1.2110887	0	0.5155147	7.7362094	8.8148776	0.6736297	0.5755345	5.7415713	-3.318471	0.0003873	0.0166268	DOWN
ENSMUSG00	H2bc21	H2B clustere	protein codi	GO:0002376	immune syst	K11252	histone H2B	mmu05203	Viral carcino	3:96221119-	100	91	152	220	239	218	2.3178015	2.1347114	3.499256	5.5612348	5.5341414	4.9184267	2.6505896	5.3379343	-1.009968	0.0003939	0.0168595	DOWN
ENSMUSG00	Serpina3m	serine (or cys	protein codi	GO:0003824	catalytic acti	-	-	-	-	12:10433848	68	32	51	115	169	87	1.841011	0.8768454	1.3714378	3.3956023	4.5710142	2.2927667	1.3630981	3.4197944	-1.32702	0.0004123	0.0175651	DOWN
ENSMUSG00	Igkv1-117	immunoglob	IG_V_gene	GO:0002376	immune syst	-	-	-	-	6:68121046-	34	5	14	150	41	53	5.1107043	0.7606139	2.0901799	24.590076	6.1567579	7.7546614	2.6538327	12.833832	-2.273803	0.0004275	0.0181016	DOWN
ENSMUSG00	Hs3st3b1	heparan sulf	protein codi	GO:0003824	catalytic acti	K07809	[heparan sul	mmu00534	Glycosaminc	11:63885792	64	62	79	204	137	111	0.7913875	0.7759219	0.9702709	2.7511127	1.6924061	1.3360484	0.8458601	1.9265224	-1.187508	0.000437	0.018419	DOWN
ENSMUSG00	Lncbate1	brown adipo	lincRNA	-	-	-	-	-	-	8:108553252	56	42	131	211	168	200	4.1880245	3.1789903	9.7308264	17.209776	12.551852	14.559431	5.6992804	14.773686	-1.374178	0.00045	0.0188509	DOWN
ENSMUSG00	Gm38244	predicted ge	TEC	-	-	-	-	-	-	3:106478283	20	35	102	139	156	190	0.4696598	0.8318395	2.3790681	3.5598687	3.6597483	4.3430484	1.2268558	3.8542218	-1.651474	0.0004524	0.0189222	DOWN
ENSMUSG00	Gm44706	predicted ge	antisense	-	-	-	-	-	-	7:79740678-	16	16	15	44	43	34	1.1922168	1.2066038	1.1100962	3.5755669	3.2008493	2.4660029	1.1696389	3.0808064	-1.397245	0.0004596	0.0191194	DOWN
ENSMUSG00	Atp1a3	ATPase, Na+	protein codi	GO:0032502	developmen	K01539	sodium/pota	mmu04024	cAMP signal	7:24978167-	428	475	217	570	1159	613	3.2574321	3.6588308	1.6403833	4.7312438	8.8122908	4.5413242	2.8522154	6.0282863	-1.079665	0.000543	0.0219824	DOWN
ENSMUSG00	Krt36	keratin 36 [S	protein codi	GO:0032502	developmen	K07604	type I keratir	mmu04915	Estrogen sig	11:10010200	40	52	54	105	141	78	1.4536687	1.9126132	1.9491805	4.1616007	5.1191331	2.7592253	1.7718208	4.0133197	-1.179563	0.0005551	0.0223723	DOWN
ENSMUSG00	2010007H0	RIKEN cDNA	antisense	-	-	-	-	-	-	9:51278513-	46	40	55	68	124	122	0.5024166	0.4421628	0.5966504	0.8099949	1.3530091	1.297049	0.5137433	1.153351	-1.166712	0.0006048	0.0240262	DOWN
ENSMUSG00	Tafa5	TAFA chemo	protein codi	GO:0023052	signaling|res	-	-	-	-	15:87544299	40	72	92	99	193	189	0.4883555	0.8896641	1.115617	1.3181905	2.3539957	2.2460902	0.8312122	1.9727588	-1.246926	0.0006485	0.0253628	DOWN
ENSMUSG00	Cldn24	claudin 24 [S	protein codi	GO:0005198	structural mo	K06087	claudin[-]	mmu04514	Cell adhesio	8:47822143-	23	21	50	61	81	97	2.1066177	1.946712	4.5486889	6.0934355	7.4118228	8.6482805	2.8673396	7.384513	-1.36479	0.0006603	0.0256062	DOWN
ENSMUSG00	Styx	serine/threon	protein codi	GO:0032502	developmen	-	-	-	-	14:45351093	308	156	128	334	458	419	0.8028075	0.4115319	0.3313797	0.949462	1.1926164	1.0630786	0.5152397	1.0683857	-1.052117	0.0006683	0.0258802	DOWN
ENSMUSG00	Slc26a10	solute carrie	protein codi	GO:0009987	cellular proc	-	-	-	-	10:12717139	78	90	111	147	328	150	0.5925305	0.6919548	0.8375165	1.2178775	2.4892193	1.1091685	0.7073339	1.6054218	-1.182489	0.0007028	0.026841	DOWN
ENSMUSG00	Pcyt1a	phosphate c	protein codi	GO:0003824	catalytic acti	K00968	choline-pho	mmu01100	Metabolic pa	16:32430921	323	431	353	1218	1113	301	1.507318	2.0356225	1.6361827	6.1989645	5.1888567	1.3672874	1.7263744	4.2517029	-1.300295	0.0007353	0.0277001	DOWN
ENSMUSG00	Trpv1	[illegible]	[illegible]	[illegible]	[illegible]	[illegible]	[illegible]	[illegible]	[illegible]	[illegible]	[illegible]	[illegible]	[illegible]	[illegible]	[illegible]	[illegible]	[illegible]	[illegible]	[illegible]	[illegible]	[illegible]	[illegible]	[illegible]	[illegible]	[illegible]	[illegible]	[illegible]	[illegible]
ENSMUSG00	Zyg11a	[illegible]	[illegible]	[illegible]	[illegible]	[illegible]	[illegible]	[illegible]	[illegible]	[illegible]	[illegible]	[illegible]	[illegible]	[illegible]	[illegible]	[illegible]	[illegible]	[illegible]	[illegible]	[illegible]	[illegible]	[illegible]	[illegible]	[illegible]	[illegible]	[illegible]	[illegible]	[illegible]
ENSMUSG00	Gm11837	[illegible]	[illegible]	[illegible]	[illegible]	[illegible]	[illegible]	[illegible]	[illegible]	[illegible]	[illegible]	[illegible]	[illegible]	[illegible]	[illegible]	[illegible]	[illegible]	[illegible]	[illegible]	[illegible]	[illegible]	[illegible]	[illegible]	[illegible]	[illegible]	[illegible]	[illegible]	[illegible]
ENSMUSG00	Gm20403	[illegible]	[illegible]	[illegible]	[illegible]	[illegible]	[illegible]	[illegible]	[illegible]	[illegible]	[illegible]	[illegible]	[illegible]	[illegible]	[illegible]	[illegible]	[illegible]	[illegible]	[illegible]	[illegible]	[illegible]	[illegible]	[illegible]	[illegible]	[illegible]	[illegible]	[illegible]	[illegible]
ENSMUSG00	[illegible]	[illegible]	[illegible]	[illegible]	[illegible]	[illegible]	[illegible]	[illegible]	[illegible]	[illegible]	[illegible]	[illegible]	[illegible]	[illegible]	[illegible]	[illegible]	[illegible]	[illegible]	[illegible]	[illegible]	[illegible]	[illegible]	[illegible]	[illegible]	[illegible]	[illegible]	[illegible]	[illegible]
ENSMUSG00	Igkv5-17	[illegible]	[illegible]	[illegible]	[illegible]	[illegible]	[illegible]	[illegible]	[illegible]	[illegible]	[illegible]	[illegible]	[illegible]	[illegible]	[illegible]	[illegible]	[illegible]	[illegible]	[illegible]	[illegible]	[illegible]	[illegible]	[illegible]	[illegible]	[illegible]	[illegible]	[illegible]	[illegible]
ENSMUSG00	Ighg2b	[illegible]	[illegible]	[illegible]	[illegible]	[illegible]	[illegible]	[illegible]	[illegible]	[illegible]	[illegible]	[illegible]	[illegible]	[illegible]	[illegible]	[illegible]	[illegible]	[illegible]	[illegible]	[illegible]	[illegible]	[illegible]	[illegible]	[illegible]	[illegible]	[illegible]	[illegible]	[illegible]
ENSMUSG00	Gm5587	[illegible]	[illegible]	[illegible]	[illegible]	[illegible]	[illegible]	[illegible]	[illegible]	[illegible]	[illegible]	[illegible]	[illegible]	[illegible]	[illegible]	[illegible]	[illegible]	[illegible]	[illegible]	[illegible]	[illegible]	[illegible]	[illegible]	[illegible]	[illegible]	[illegible]	[illegible]	[illegible]
ENSMUSG00	C530005A16	[illegible]	[illegible]	[illegible]	[illegible]	[illegible]	[illegible]	[illegible]	[illegible]	[illegible]	[illegible]	[illegible]	[illegible]	[illegible]	[illegible]	[illegible]	[illegible]	[illegible]	[illegible]	[illegible]	[illegible]	[illegible]	[illegible]	[illegible]	[illegible]	[illegible]	[illegible]	[illegible]
ENSMUSG00	Gm20390	[illegible]	[illegible]	[illegible]	[illegible]	[illegible]	[illegible]	[illegible]	[illegible]	[illegible]	[illegible]	[illegible]	[illegible]	[illegible]	[illegible]	[illegible]	[illegible]	[illegible]	[illegible]	[illegible]	[illegible]	[illegible]	[illegible]	[illegible]	[illegible]	[illegible]	[illegible]	[illegible]
ENSMUSG00	Gm42248	[illegible]	[illegible]	[illegible]	[illegible]	[illegible]	[illegible]	[illegible]	[illegible]	[illegible]	[illegible]	[illegible]	[illegible]	[illegible]	[illegible]	[illegible]	[illegible]	[illegible]	[illegible]	[illegible]	[illegible]	[illegible]	[illegible]	[illegible]	[illegible]	[illegible]	[illegible]	[illegible]
ENSMUSG00	Iglc2	[illegible]	[illegible]	[illegible]	[illegible]	[illegible]	[illegible]	[illegible]	[illegible]	[illegible]	[illegible]	[illegible]	[illegible]	[illegible]	[illegible]	[illegible]	[illegible]	[illegible]	[illegible]	[illegible]	[illegible]	[illegible]	[illegible]	[illegible]	[illegible]	[illegible]	[illegible]	[illegible]
ENSMUSG00	Gm45199	[illegible]	[illegible]	[illegible]	[illegible]	[illegible]	[illegible]	[illegible]	[illegible]	[illegible]	[illegible]	[illegible]	[illegible]	[illegible]	[illegible]	[illegible]	[illegible]	[illegible]	[illegible]	[illegible]	[illegible]	[illegible]	[illegible]	[illegible]	[illegible]	[illegible]	[illegible]	[illegible]

Table S3-Down genes

	BAT	IngWAT
1	Acly	Acly
2	Acss2	Acaca
3	Cfd	Fasn
4	Acaca	Cyp2e1
5	Slc25a1	Tkt
6	Me1	Acss2
7	Fasn	Thrsp
8	Thrsp	Slc25a1
9	Elovl6	Me1
10	Tkt	Cfd
11	Mup22	Slc2a5
12	Hcar2	Mup22
13	Cyp2f2	Rassf6
14	Ptges	Cyp2f2
15	Slc2a5	Elovl6
16	Cldn24	Tst
17	Atp1a3	Orm3
18	Cyp2e1	Pth1r
19	Gsta3	Ptges
20	Gpx8	Oscp1
21	Mapk8ip1	Cldn24
22	Pth1r	Gsta3
23	Oscp1	Mapk8ip1
24	Orm3	Atp1a3
25	Rassf6	Gpx8
26	Aacs	Aacs
27	Tst	Hcar2
28	Gm28875	AA914427
29	AA914427	Gm5182
30	Gm454	Igkc
31	Gm7329	Gm7329
32	Gm5182	Gm7049
33	Gm7049	Gm454
34	Igkc	Gm28875

Table S4-Rosiglitazone-InterfaceInteractions

ResidueName	:GLU283	:GLU314	:ASP317	:GLU318	:LEU320	:GLN321	:LYS322	:CYS323	:GLN324	:GLU325	:LYS326	:GLY327	:PHE328	:PRO329	:VAL330	:ARG331	:CYS332	:CYS333	:ILE334	:SER351	:GLN352	:SER353	:PRO355	:LYS358	:ARG359	:SER360	:ASP363	:SER367	:TRP368	:ASN372	:ILE375	:GLU700	:THR731	:LYS736	:GLU740	:LYS743	:GLU751
D:LYS224	-	-	-	-	-	-	-	-	-	-	-	-	-	-	-	-	-	-	-	-	-	-	-	-	-	-	-	-	-	-	-	-	N	N	N	-	-
D:PRO227	-	-	-	-	-	-	-	-	-	-	-	-	-	-	-	-	-	-	-	-	-	-	-	-	-	-	-	-	-	-	-	N	-	-	-	-	-
D:LEU228	-	-	-	-	-	-	-	-	-	-	-	-	-	-	-	-	-	-	-	-	-	-	-	-	-	-	-	-	-	-	-	N	-	-	-	-	-
D:LEU255	-	-	-	-	-	-	-	-	N	-	-	-	-	-	-	H	-	-	-	-	-	-	-	-	-	-	-	-	-	-	-	-	-	-	-	-	-
D:MET256	-	-	-	-	-	-	-	-	-	-	-	-	-	-	-	H	-	-	-	-	-	-	-	-	-	-	-	-	-	-	-	-	-	-	-	-	-
D:GLY258	-	-	-	-	-	-	-	-	-	-	-	-	-	-	-	N	-	-	-	-	-	-	-	-	-	-	-	-	-	-	-	-	-	-	-	-	-
D:GLU259	-	-	-	-	-	-	-	-	-	-	-	-	-	-	-	N	-	-	-	-	-	-	-	-	-	-	-	-	-	-	-	-	-	-	-	-	-
D:GLU276	-	-	-	-	-	-	-	-	-	-	-	-	-	-	-	-	N	N	H	-	-	-	-	-	-	-	-	-	-	-	-	-	-	-	-	-	-
D:VAL277	-	-	-	-	-	-	-	-	N	-	-	-	-	-	-	-	-	-	-	-	-	-	-	-	-	-	-	-	-	-	-	-	-	-	-	-	-
D:ILE279	-	-	-	-	-	-	-	N	N	-	-	-	-	-	-	-	-	-	-	-	-	-	-	-	-	-	-	-	-	-	-	-	-	-	-	-	-
D:ARG280	-	-	-	-	-	-	-	N	H	N	-	-	-	-	-	N	N	-	-	-	-	-	-	-	-	-	-	-	-	-	-	-	-	-	-	-	-
D:ILE281	-	-	-	-	-	-	-	N	H	-	N	-	-	-	-	-	-	-	-	-	-	-	-	-	-	-	-	-	-	-	-	-	-	-	-	-	-
D:GLN283	-	-	-	-	N	N	N	H	N	N	-	-	-	-	-	-	-	-	-	-	-	-	-	-	-	-	-	-	-	-	-	-	-	-	-	-	-
D:GLY284	-	-	-	-	-	N	-	N	H	N	N	N	-	-	-	-	-	-	-	-	-	-	-	-	-	-	-	-	-	-	-	-	-	-	-	-	-
D:CYS285	-	-	-	-	-	-	-	-	N	N	N	-	-	-	-	-	-	-	-	-	-	-	-	-	-	-	-	-	-	-	-	-	-	-	-	-	-
D:GLN286	-	-	-	-	-	H	-	-	-	-	-	-	-	-	-	-	-	-	-	-	-	-	-	-	-	-	-	-	-	-	-	-	-	-	-	-	-
D:PHE287	N	-	-	N	-	N	Pi	-	-	Pi	-	-	N	-	N	-	-	-	-	-	-	-	-	-	-	-	-	-	-	-	-	-	-	-	-	-	-
D:ARG288	-	-	-	-	-	N	-	-	-	H	H	N	N	-	-	-	-	-	-	-	-	-	-	-	-	-	-	-	-	-	-	-	-	-	-	-	-
D:GLU291	-	-	-	-	-	-	-	-	-	-	-	-	N	-	-	-	-	-	-	-	-	-	-	-	-	-	-	-	-	-	-	-	-	-	-	-	-
D:GLN294	-	-	-	-	-	-	-	-	-	-	-	-	-	-	-	-	-	-	-	-	-	-	-	-	-	-	-	-	-	-	-	-	-	-	-	N	N
D:GLU298	-	-	-	-	-	-	-	-	-	-	-	-	-	-	-	-	-	-	-	-	-	-	-	-	-	-	-	-	-	-	-	-	-	N	-	-	-
D:LEU330	-	-	-	-	-	-	-	-	-	-	N	-	-	-	-	-	-	-	-	-	-	-	-	-	-	-	-	-	-	-	-	-	-	-	-	-	-
D:LEU340	-	-	-	-	-	-	-	-	-	-	N	-	-	-	-	-	-	-	-	-	-	-	-	-	-	-	-	-	-	-	-	-	-	-	-	-	-
D:ILE341	-	-	-	-	-	-	-	-	-	N	N	N	-	-	-	-	-	-	-	-	-	-	-	-	-	-	-	-	-	-	-	-	-	-	-	-	-
D:SER342	-	-	-	-	-	-	-	-	-	N	H	H	N	N	-	-	-	-	-	-	-	-	-	-	-	-	-	-	-	-	-	-	-	-	-	-	-
D:GLU343	-	-	-	-	-	-	-	-	-	-	N	N	Pi	-	-	-	-	-	-	-	-	-	-	-	-	-	-	-	-	-	-	N	-	-	-	-	-
D:MET364	-	-	-	-	-	-	-	-	-	-	N	-	-	-	-	-	-	-	-	-	-	-	-	-	-	-	-	-	-	-	-	-	-	-	-	-	-
D:LYS457	-	-	-	-	-	-	-	-	-	-	-	-	-	-	-	-	-	-	-	-	-	N	N	-	-	-	-	-	-	N	-	-	-	-	-	-	-
D:LYS458	-	-	-	-	-	-	-	-	-	-	-	-	-	-	-	-	-	-	-	-	-	-	-	H	-	-	-	-	-	-	-	-	-	-	-	-	-
D:GLU460	-	-	-	-	-	-	-	-	-	-	-	-	-	-	-	-	-	-	-	-	-	-	-	-	-	-	-	-	-	N	-	-	-	-	-	-	-
D:THR461	-	-	-	-	-	-	-	-	-	-	-	-	-	-	-	-	-	-	-	-	-	-	-	N	H	H	N	N	-	N	N	-	-	-	-	-	-
D:ASP462	-	-	-	-	-	-	-	-	-	-	-	-	-	-	-	-	-	-	-	-	-	-	-	-	N	-	-	N	-	N	N	-	-	-	-	-	-
D:MET463	-	-	-	-	-	-	-	-	-	-	-	-	-	-	-	-	-	-	-	-	N	N	-	-	-	-	-	N	N	N	N	-	-	-	-	-	-
D:SER464	-	-	N	N	N	N	-	-	-	-	-	-	-	-	-	-	-	-	-	-	-	-	-	-	-	-	-	H	N	-	-	-	-	-	-	-	-
D:LEU465	-	-	N	-	-	N	-	-	-	-	-	-	-	-	-	-	-	-	-	N	-	-	-	-	-	-	-	-	-	-	-	-	-	-	-	-	-
D:HIS466	-	-	N	N	-	N	-	-	-	-	-	-	-	-	-	-	-	-	-	-	-	-	-	-	-	-	-	-	-	-	-	-	-	-	-	-	-
D:PRO467	-	N	N	N	-	-	-	-	-	-	-	-	-	-	-	-	-	-	-	-	-	-	-	-	-	-	-	-	-	-	-	-	-	-	-	-	-
D:LEU469	-	-	-	-	-	N	-	-	-	-	-	-	-	-	-	-	-	-	-	-	-	-	-	-	-	-	-	-	-	-	-	-	-	-	-	-	-
D:GLN470	-	-	-	-	-	-	-	-	-	-	-	-	-	-	-	-	-	-	-	N	N	-	-	-	-	-	-	-	N	-	-	-	-	-	-	-	-

Table S5-GW9662-InterfaceInteractions

ResidueName	:LYS144	:TRP149	:LYS150	:THR151	:PRO152	:ASN183	:TYR186	:ASP190	:VAL193	:HIS194	:GLU195	:LYS196	:ASP200	:THR221	:HIS223	:GLN224	:LEU226	:VAL227	:CYS230	:GLN231	:ARG268	:GLN386	:GLU387	:ALA388	:GLY389	:ASP390	:GLU391	:CYS392	:GLU393	:PRO394	:GLU395	:TRP396
D:LYS216	N	Pi	N	-	-	-	N	-	-	-	-	-	-	-	-	-	-	-	-	-	-	-	-	-	-	-	-	-	-	-	-	-
D:HIS217	-	-	N	H	Pi	-	-	-	-	-	-	-	-	-	-	-	-	-	-	-	-	-	-	-	-	-	-	-	-	-	-	N
D:TYR219	-	N	-	-	-	-	N	-	-	N	H	-	-	-	-	-	-	-	-	-	-	-	-	-	-	-	-	-	-	-	-	-
D:ASP220	-	N	H	N	-	-	N	-	-	-	-	-	-	-	-	-	-	-	-	-	-	-	-	-	-	-	-	-	-	-	-	N
D:SER221	-	-	-	N	-	-	-	-	-	-	-	-	-	-	-	-	-	-	-	-	-	-	-	-	-	-	-	-	-	-	-	N
D:ILE223	-	-	-	-	-	-	Pi	N	-	Pi	-	-	-	-	-	-	-	-	-	-	N	-	-	-	-	-	-	-	N	H	-	-
D:LYS224	-	N	N	N	-	N	Pi	-	-	-	-	-	-	-	-	-	-	-	-	-	-	-	-	-	-	-	-	-	N	N	N	Pi
D:SER225	-	-	-	-	-	-	-	-	-	-	-	-	-	-	-	-	-	-	-	-	-	-	-	-	-	-	-	-	N	N	-	N
D:PHE226	-	-	-	-	-	-	-	-	-	-	-	-	-	-	-	-	-	-	-	-	-	-	-	-	-	-	-	N	N	N	-	-
D:PRO227	-	-	-	-	-	-	-	-	-	N	-	-	-	-	-	-	-	-	-	-	N	-	-	-	-	-	N	N	N	-	-	-
D:LEU228	-	-	-	-	-	-	-	-	-	-	-	-	-	-	-	-	-	-	-	-	-	-	-	-	-	N	N	N	-	-	-	-
D:THR229	-	-	-	-	-	-	-	-	-	H	-	-	-	-	-	-	-	-	-	-	-	-	-	-	-	-	-	-	-	-	-	-
D:ALA231	-	-	-	-	-	-	-	-	N	Pi	N	N	-	-	N	-	-	-	-	-	-	-	-	-	-	-	-	-	-	-	-	-
D:LYS232	-	-	-	-	-	-	-	N	N	Pi	-	-	-	-	Pi	-	N	-	N	-	N	-	-	-	-	N	N	N	-	-	-	-
D:ARG234	-	-	-	-	-	-	-	-	-	-	-	N	-	-	-	-	-	-	-	-	-	-	-	-	-	-	-	-	-	-	-	-
D:ALA235	-	-	-	-	-	-	-	-	-	-	-	N	-	-	Pi	-	-	-	-	-	-	-	-	-	-	-	-	-	-	-	-	-
D:ILE236	-	-	-	-	-	-	-	-	-	-	-	N	-	-	N	-	-	-	-	-	-	-	-	-	-	N	-	-	-	-	-	-
D:LYS240	-	-	-	-	-	-	-	-	-	-	-	N	N	N	N	-	-	-	-	-	-	-	-	-	-	-	-	-	-	-	-	-
D:THR241	-	-	-	-	-	-	-	-	-	-	-	N	-	-	H	N	-	-	-	-	-	-	-	-	-	-	-	-	-	-	-	-
D:THR242	-	-	-	-	-	-	-	-	-	-	-	-	-	N	N	N	-	-	-	-	-	-	-	-	-	-	-	-	-	-	-	-
D:ASP243	-	-	-	-	-	-	-	-	-	-	-	-	-	-	N	N	-	N	-	-	-	-	-	-	-	-	-	-	-	-	-	-
D:LYS244	-	-	-	-	-	-	-	-	-	-	-	-	-	-	-	-	-	-	-	N	-	N	-	-	-	-	-	-	-	-	-	-
D:ILE262	-	-	-	-	-	-	-	-	-	-	-	-	-	-	-	-	-	-	-	-	-	N	N	-	-	-	-	-	-	-	-	-
D:GLU295	-	-	-	-	-	-	-	-	-	-	-	-	-	-	-	-	-	-	-	-	-	-	-	-	-	-	-	-	N	-	-	-
D:SER302	-	-	-	-	-	-	-	-	-	-	-	-	-	-	-	-	-	-	-	-	-	-	-	-	-	-	-	-	-	-	-	N
D:GLU343	-	-	-	-	-	-	-	-	-	-	-	-	-	-	-	-	-	-	-	-	-	-	-	-	N	N	H	N	-	-	-	-
D:GLY344	-	-	-	-	-	-	-	-	-	-	-	-	-	-	-	-	-	-	-	-	-	-	-	-	-	N	N	-	-	-	-	-
D:GLN345	-	-	-	-	-	-	-	-	-	-	-	-	-	-	-	-	-	-	-	H	-	N	N	H	N	N	-	-	-	-	-	-
D:ASP381	-	-	-	-	-	-	-	-	-	H	-	-	-	-	-	-	-	-	-	-	-	-	-	-	-	-	-	-	-	-	-	-

Table S6 Primers sequence

Primers	Forward (5'to3')	Reverse (5'to3')
m18S	CGCGGTTCTATTTTGTTGGT	AGTCGGCATCGTTTATGGTC
mACSS2	GGGAGTTTTGGGGAAACATT	CCAGCACGTTGTAGCAGATG
mUCP1	ACTGCCACACCTCCAGTCATT	CTTTGCCTCACTCAGGATTGG
mPPARγ	GAAAGACAACGGACAAATCACC	GGGGGTGATATGTTTGAACTT

Table S7 Primers sequence

Primers	Forward (5'to3')	Reverse (5'to3')
VN173-ACSS2-pBiFC	ACGCGTCGACATGGGGCTTCCTGAGGAGCGGGTCC(SalI)	GCTCTAGACTGGATGGTCAGGCAGCGGTGGCTGAAG(XbaI)
VC155-PPAR-α-pBiFC	ACGCGTCGACATGGTGGACACGGAAAGCCCACTCTG(SalI)	GGGGTACCGTACATGTCCCTGTAGATCTCCTGC(KpnI)
VC155-PPAR-β-pBiFC	ACGCGTCGACATGGAGCAGCCACAGGAGGAAGCCCC(SalI)	GGGGTACC GTACATGTCCTTGTAGATCTCCTGG (KpnI)
VC155-PPAR-γ-pBiFC	ACGCGTCGACATGACCATGGTTGACACAGAGATGCC(SalI)	GGGGTACCGTACAAGTCCTTGTAGATCTCCTGCAGG(KpnI)
h-PPAR-γ insoform1-PCMV	ACGCCCCGGGTATGACCATGGTTGACACAGAGATG (SmaI)	CCGCTCGAGCTAGTACAAGTCCTTGTAGATCTCC (XhoI)
h-ACSS2 -insoform1-PCMV	CG GGATCC ATGGGGCTTCCTGAGGAGCG(BamHI)	GC TCTAGA TCACTGGATGGTCAGGCAGCGGTG(XbaI)
**Primers(Bacteria)**	**Forward (5'to3')**	**Reverse (5'to3')**
h-ACSS2 insoform1-PET28a	CG GGATCC ATGGGGCTTCCTGAGGAGCG(BamHI)	AAGGAAAAAAGCGGCCGC TCACTGGATGGATGGTCAGG(NotI)
h-PPAR-γ insoform1-PGEX	ACGCCCCGGGTATGACCATGGTTGACACAGAGATG (SmaI)	CCGCTCGAG CTAGTACAAGTCCTTGTAGATCTCC (XhoI)
h-PPAR-γ insoform2-PGEX	ACGCCCCGGGTATGGGTGAA ACTCTGGGAG ATTCTCC (SmaI)	CCGCTCGAGCTAGTACAAGTCCTTGTAGATCTCC (XhoI)
m-ACSS2 insoform1-PET28a	GGAATTCATGGGGCTTCCCGAGG AGCGGCGC(EcoRI)	AAGGAAAAAAGCGGCCGCTCACTGGGTG GTCAGGCAGCGGTGAC(NotI)
**Mutant primer**	**Forward (5'to3')**	**Reverse (5'to3')**
VC155-PPARγ-ΔLBD	CAGAGAGTCCTGAGCCACTGCCAACATTTC	ATTCATGTCA TAGATAACGA ATGGTGATTT
VC155-PPARγ-ΔDBD	AATGCCATCAGGTTTGGGCGGATGCCACAG	ACGACATTCAATTGCCATGAGGGAGTTGGA
VC155-PPARγ-K154Q	ACCAAAAAAGTAGAAATAAATGTCAGTACT	GGATCCGACAGTTAAGATCACATCTGTCAT
VC155-PPARγ-K154R	ACAGAAAAAGTAGAAATAAATGTCAGTACT	GGATCCGACAGTTAAGATCACATCTGTCAT
VC155-PPARγ-K155Q	AACAAAGTAGAAATAAATGTCAGTACTGTC	TGTGGATCCG ACAGTTAAGATCACATCTGT
VC155-PPARγ-K155R	AAAGAAGTAGAAATAAATGTCAGTACTGTC	TGTGGATCCG ACAGTTAAGATCACATCTGT
VC155-PPARγ-K154/155Q	ACCAACAAAGTAGAAATAAATGTCAGTACT	GGATCCGACA GTTAAGATCA CATCTGTCAT
VC155-PPARγ-K154/155R	ACAGAAGAAGTAGAAATAAATGTCAGTACT	GGATCCGACA GTTAAGATCA CATCTGTCAT
VC155-PPARγ-K238Q	GGACAGACAACAGACAAATCACCATTCGTT	TGTCAAGATCGCCCTCGCCTTTGCTTTGGT
VC155-PPARγ-K238R	GGAAGGACAACAGACAAATCACCATTCGTT	TGTCAAGATCGCCCTCGCCTTTGCTTTGGT
VC155-PPARγ-K263Q	GTTCCAACACATCACCCCCCTGCAGGAGCA	TTGATTTTATCTTCTCCCATCATTAAGGAA
VC155-PPARγ-K263R	GTTCAGACACATCACCCCCCTGCAGGAGCA	TTGATTTTATCTTCTCCCATCATTAAGGAA
VN173-ACSS2-HAN(1-107aa)	CGGGATCCATGGGGCTTCCTGAGGAGCGGGTCCGGAGC	AAGGAAAAAAGCGGCCGCTCATACATTGTAGCAGATGTTGGT AGTTGC
VN173-ACSS2-HAC(108-701aa)	CGGGATCCATGCTGGATCGAAATGTCCATGAG	AAGGAAAAAAGCGGCCGCTCACTGGATGGTCAGGCAGCAGC GGTGGCTG
VN173-ACSS2-E239R	CAGAGGAAGGGTTTCCCAGTAAGATGCTGC	ACACTTCTGCAGGGCCTCGTCAGCCAGCTC
VN173-ACSS2-S267A	CAGGCCCCCCCAATTAAGAGGTCATGCC	GCTGGTGGAGTCACCCATGCCGAGCTCTGC
VN173-ACSS2-S273A	CAGGCCCCCCCAATTAAGAGGTCATGCC	GCTGGTGGAGTCACCCATGCCGAGCTCTGC
VN173-ACSS2-S280A	CAGGCCCCCCCAATTAAGAGGTCATGCC	GCTGGTGGAGTCACCCATGCCGAGCTCTGC
VN173-ACSS2-E596A	CAGGCCCCCCCAATTAAGAGGTCATGCC	GCTGGTGGAGTCACCCATGCCGAGCTCTGC
VN173-ACSS2-S659A	GCGCAGGGAAAATCATGAGGCGAGTGCTTC	GGGTTTTAGGCAAGCCAGGTGCATTCTGGA
VN173-ACSS2-T363K	CAAGGGTCATTCCTACGTCACCTATGGGCC	ATCCAACCAATGTCTGCCGTGCACCAGAAC
VN173-ACSS2-S30A	GGGCTCCGCCGCCCGAGGTCAGCCGCTCCG	AACTCCGCGCCCGGCCTCCGGCTCCAGCTT
VN173-ACSS2-S30E	GGGAACCGCCGCCCGAGGTCAGCCGCTCCG	AACTCCGCGCCCGGCCTCCGGCTCCAGCTT
